# Chemical proteomic profiling reveals protein interactors of the alarmones diadenosine triphosphate and tetraphosphate

**DOI:** 10.1038/s41467-021-26075-4

**Published:** 2021-10-04

**Authors:** Lena Krüger, Christoph J. Albrecht, Hannah K. Schammann, Florian M. Stumpf, Marie L. Niedermeier, Yizhi Yuan, Katrin Stuber, Josua Wimmer, Florian Stengel, Martin Scheffner, Andreas Marx

**Affiliations:** 1 Department of Chemistry, University of Konstanz, Konstanz, Germany; 2 Konstanz Research School-Chemical Biology, University of Konstanz, Konstanz, Germany; 3 Department of Biology, University of Konstanz, Konstanz, Germany

**Keywords:** Nucleotide-binding proteins, Proteomics, Chemical tools, Target identification

## Abstract

The nucleotides diadenosine triphosphate (Ap_3_A) and diadenosine tetraphosphate (Ap_4_A) are formed in prokaryotic and eukaryotic cells. Since their concentrations increase significantly upon cellular stress, they are considered to be alarmones triggering stress adaptive processes. However, their cellular roles remain elusive. To elucidate the proteome-wide interactome of Ap_3_A and Ap_4_A and thereby gain insights into their cellular roles, we herein report the development of photoaffinity-labeling probes and their employment in chemical proteomics. We demonstrate that the identified Ap_*n*_A interactors are involved in many fundamental cellular processes including carboxylic acid and nucleotide metabolism, gene expression, various regulatory processes and cellular response mechanisms and only around half of them are known nucleotide interactors. Our results highlight common functions of these Ap_*n*_As across the domains of life, but also identify those that are different for Ap_3_A or Ap_4_A. This study provides a rich source for further functional studies of these nucleotides and depicts useful tools for characterization of their regulatory mechanisms in cells.

## Abstract (web summary)

Diadenosine polyphosphates (ApAs) are involved in cellular stress signaling but only a few molecular targets have been characterized so far. Here, the authors develop Ap_*n*_A-based photoaffinity-labeling probes and use them to identify Ap_3_A and Ap_4_A binding proteins in human cell lysates.

## Introduction

Dinucleoside polyphosphates (Np_*n*_Ns) are a family of nucleotides which were discovered in bacteria and eukaryotic cells already in the 1960s^1^, but their cellular roles and functions remain mostly elusive. The most widely studied family members are diadenosine polyphosphates (Ap_*n*_As), like diadenosine triphosphate Ap_3_A and diadenosine tetraphosphate Ap_4_A^2,3^. As their cellular concentrations increase in response to extracellular cues such as pH, temperature and oxidative stress from a nanomolar to a lower micromolar range, Ap_*n*_As have been considered to be alarmones that signal cellular stress^2,4,5^. However, the underlying mechanisms and pathways remain to be elucidated.

It is assumed that the main source of intracellular Ap_n_As is a side reaction of aminoacyl-tRNA synthetases (aaRSs)^6^, since several studies have shown that across species aaRSs can produce Ap_n_As. Recently, it was reported that activation of the posttranslational modifiers ubiquitin and ubiquitin-like proteins (i.e., SUMO, NEDD8) is accompanied by the formation of Ap_4_A and Ap_3_A linking one of the most prevalent eukaryotic protein modification systems to Ap_n_A formation^7^.

In higher eukaryotes, two proteins have been identified that are responsible for the degradation of Ap_3_A and Ap_4_A. In humans, Ap_3_A is cleaved into AMP and ADP by the tumor suppressor protein fragile histidine triad (FHIT)^8^, whereas Nudix (nucleoside diphosphate linked to X) type motif 2 (NUDT2) hydrolyzes Ap_4_A to AMP and ATP^9^. The malfunction of these enzymes results in severe consequences for the affected cells or organisms. Dysfunction or absence of FHIT are frequently observed in various types of cancer^10^. NUDT2 has been proposed as a prognostic marker for breast and lung carcinoma, due to its enhanced activity in these carcinogenic tissues^11^. The absence of NUDT2, on the other hand, causes downregulation of the immune response^12^.

**Fig. 1:**
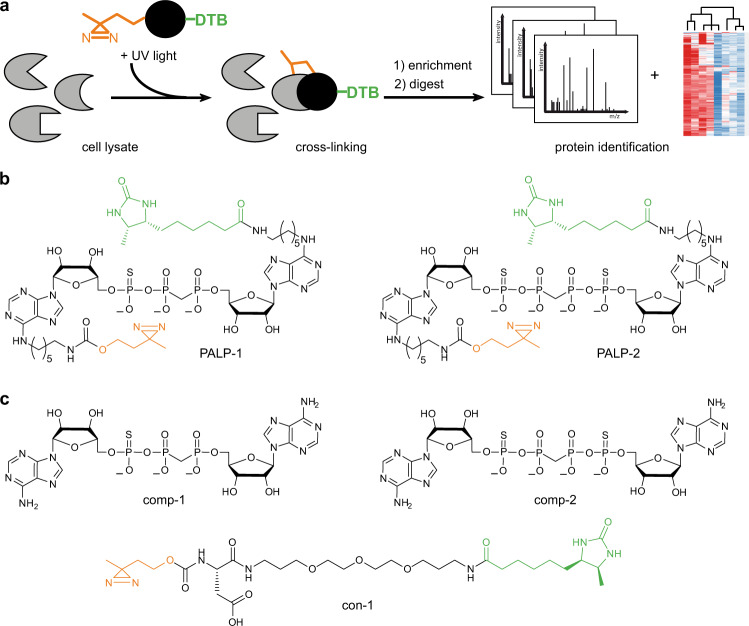
Overview of photoaffinity-labeling (PAL) approach for identifying Ap_n_A interactors. **a** Schematic workflow of PAL experiments. Probes were incubated with cell lysates and irradiated with UV light to initiate photo-crosslinking. Labeled proteins were affinity-purified using desthiobiotin (DTB) as affinity tag. Eluted fractions were digested and analyzed by LC-MS/MS and label-free quantification (LFQ). **b** Functionalized, non-hydrolysable Ap_3_A and Ap_4_A derivatives used as PAL probes (PALP). **c** Unlabeled, non-hydrolysable Ap_3_A and Ap_4_A derivatives and a control substance (con-1) lacking the Ap_n_A scaffold served as controls in PAL experiments.

In this work, we report the development and chemoproteomic evaluation of Ap_*n*_A-based photoaffinity-labeling probes (PALPs) (Fig. 1) to elucidate the interactome of Ap_3_A and Ap_4_A and eventually gain insights into their intracellular roles. These PALPs are equipped with diazirine (DA) as photoreactive group, enabling the formation of a covalent bond between the probe and an interacting protein upon UV irradiation, and desthiobiotin (DTB) as affinity tag. By applying these PALPs in photoaffinity enrichment assays followed by LC-MS/MS (Fig. 1a), we identified 61 significantly enriched proteins for Ap_3_A and 26 for Ap_4_A from human embryonic kidney (HEK293T) cell lysates. Interestingly, while nine proteins were identified with both Ap_*n*_A baits, 52 proteins were exclusively found for Ap_3_A and 17 for Ap_4_A, respectively. Gene ontology (GO) analysis reveals the involvement of these potential Ap_n_A interactors in different fundamental cellular processes including carboxylic acid and nucleotide metabolism, gene expression, and various regulatory processes. In addition, we used our PALPs to probe for interactors in *E. coli* cells and identified overall 20 significantly enriched proteins. These proteins are also mainly associated with metabolism highlighting a common role of these Ap_n_As across the domains of life. Moreover, only about half of the identified proteins are known nucleotide binders which indicates roles of Ap_3_A and Ap_4_A in cellular pathways that are distinguishable from those of other nucleotides. Taken together, this study demonstrates the power of our chemical proteomics approach based on the synthesized PALPs and uncovers proteome-wide interaction maps for Ap_3_A and Ap_4_A. This provides a rich source for further functional studies directed towards a deeper understanding of the physiological and patho-physiological roles of these nucleotides.

## Results

### Design and synthesis of PALPs

To unravel the interactome of Ap_3_A and Ap_4_A we designed and employed photoaffinity-labeling probes that contain surrogates of the phosphoanhydride bonds in order to confer hydrolytic stability, as we observed that natural Ap_*n*_As are hydrolyzed to different extent in cell lysates (Supplementary Fig. 1). Previous studies showed that the stability of Ap_*n*_As in cell lysates is increased by exchanging the P_α_ oxygen for sulfur and replacing the bridging oxygen with a methylene group^13^. Therefore, we synthesized the Ap_3_A-analog PALP-1 and Ap_4_A-analog PALP-2 (Fig. 1b) that contain thiomonophosphate at the α-phosphate positions^14^ and methylene bisphosphonate^15^ linkages.

Due to the proficiency of several enzymes to use *N*6-modified ATP and *N*6-modified Ap_3_A as substrates, the *N*6 positions of the adenosine moieties were chosen for the attachment of the photo-crosslinking diazirine (DA) and the affinity tag desthiobiotin (DTB) in our probes^16–18^. The used DA represents a small functional group that undergoes carbene formation under UV light that rapidly inserts into nearby C–H, N–H, and O–H bonds, rendering DAs well-established tools for photoaffinity labeling^19,20^. DTB as affinity tag allows enrichment of tagged proteins by exploiting the affinity of DTB to (strept)avidin, and its specific elution with biotin for subsequent analysis by high-resolution mass spectrometry (MS)^21,22^. The synthesis of both probes was straightforward and is described in detail in Supplementary Figs. 2 and 3. To further increase the specificity of our approach, two non-photoreactive derivatives comp-1 and comp-2 (Fig. 1c) were synthesized following described routes^23,24^ and employed in competition experiments. Similarly, to exclude proteins from the interactomes that bind to the diazirine or desthiobiotin groups of our Ap_3_A and Ap_4_A probes, molecule con-1 was synthesized (Fig. 1c) and applied as an additional control.

### Ap_3_A and Ap_4_A are present in HEK293T cell lysates

**Fig. 2:**
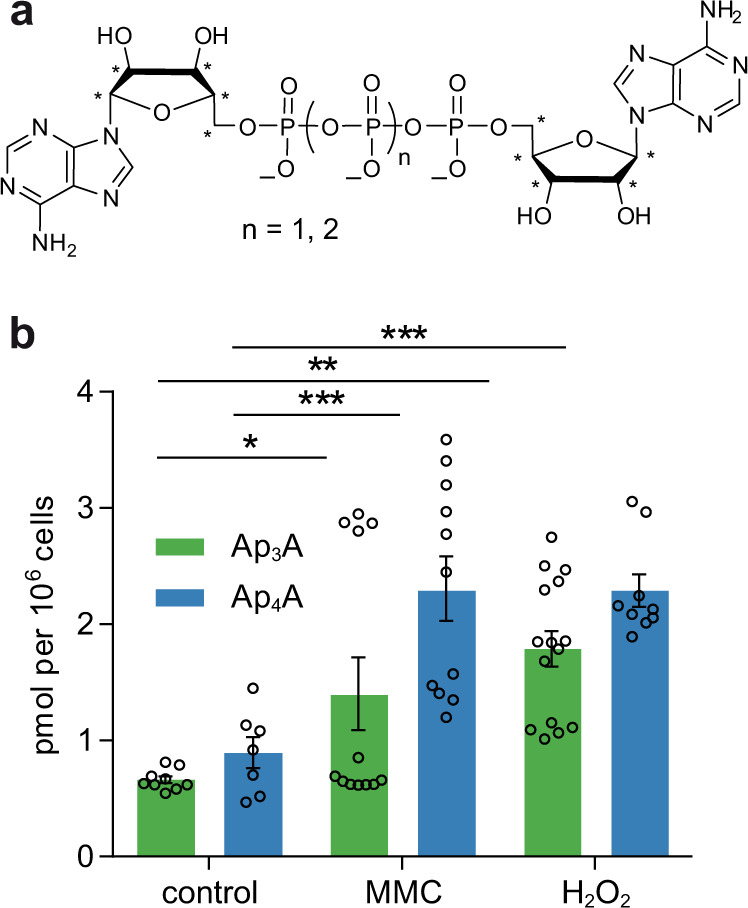
Determination of Ap_3_A and Ap_4_A levels in HEK293T cell lysates. **a** Chemical structures of ^13^C-labeled ^13^C_10_-Ap_3_A (*n* = 1) and ^13^C_10_-Ap_4_A (*n* = 2) used as internal standards. **b** Obtained Ap_3_A (green) and Ap_4_A (blue) levels of stressed and unstressed (control) HEK293T cells. For each condition 15 biological replicates were performed. Samples were measured in technical duplicates and averaged. Measurements with a signal to noise ratio below 7 were excluded. Data presented are mean ± standard error of the mean (SEM); *n* (control, Ap_3_A) = 9, *n* (control, Ap_4_A) = 7, *n* (MMC, Ap_3_A) = 12, *n* (MMC, Ap_4_A) = 11, *n* (H_2_O_2_, Ap_3_A) = 15, *n* (H_2_O_2_, Ap_4_A) = 9. Significant differences were determined by one-way ANOVA in combination with the Dunnett test (*p ≤ 0.05; **p ≤ 0.01; ***p ≤ 0.001). Source data are provided as a Source Data file.

As HEK293 cells are commonly used in interactome analyses and already have been employed to study the correlation between intracellular Ap_3_A concentrations and apoptosis^25^, we chose the HEK293T cell line for our experiments. To obtain further insights into the responsiveness of HEK293T cells towards external stress, we determined the cellular levels of Ap_3_A and Ap_4_A and their changes upon exposure to different stress conditions such as mitomycin C (MMC)-induced DNA damage and oxidative stress by treatment with H_2_O_2_. To do so, we developed a method to quantify Ap_3_A and Ap_4_A levels in cell lysates via high-resolution electrospray ionization mass spectrometry (HR-ESI-MS) employing synthesized ^13^C-labeled compounds ^13^C_10_-Ap_3_A and ^13^C_10_-Ap_4_A as internal standards (Fig. 2a). By this, we determined the level of Ap_3_A to be 0.7 ± 0.1 pmol per 10^6^ cells and of Ap_4_A level at 0.9 ± 0.4 pmol per 10^6^ cells under unstressed conditions, while their levels significantly increased upon treatment of cells with MMC or H_2_O_2_ (Fig. 2b). These results confirm that Ap_3_A and Ap_4_A are present in HEK293T cells and their concentration increases upon exposure to stressors.

### Proteomic profiling of the Ap_3_A and Ap_4_A protein interactome

**Fig. 3:**
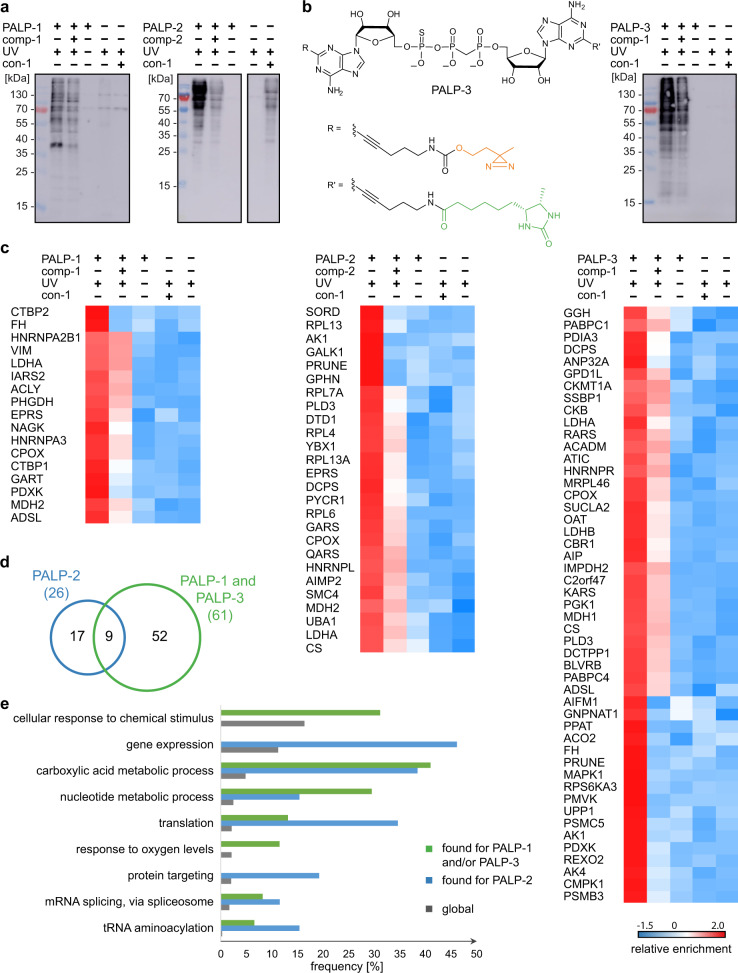
Overview of the proteomic analysis of the PAL experiments using HEK293T cell lysates. **a** Western blot analysis with ExtrAvidin®-Peroxidase showing the enrichment of labeled proteins under optimized conditions using PALP-1 (left) and PALP-2 (right) as indicated after irradiation with UV light (365 nm) for 5 min. **b** As for **a** but usage of PALP-3. Structure of PALP-3 is shown. **c** Heat map representation (*Z*-scores) of proteins that were quantified in the respective experiments in at least four out of six measurements and that passed a one-way ANOVA-based multiple-sample test for statistically significant enrichment, with a permutation-based false discovery rate (FDR) below or equal to 0.01 and *S*_0_ value set to 0.2, followed up by two-sided *post hoc* Tukey’s HSD test (FDR ≤ 0.05) to filter for significant enrichment against all performed control experiments. **d** Venn diagram showing the distribution of identified proteins for Ap_3_A probes (data obtained with PALP-1 and PALP-3, green) and the Ap_4_A probe (data obtained with PALP-2, blue). **e** Extract of biological processes that are significantly overrepresented in proteins found with Ap_3_A-based probes (PALP-1 and PALP-3, green) and the Ap_4_A-based probe (PALP-2, blue) compared to the global human proteome (gray) according to GO analysis using a one-sided hypergeometric test performed with the Cytoscape BiNGO app (Supplementary Data 2). Global frequency represents the number of genes annotated to a GO term in the entire human proteome, while sample frequency represents the number of genes annotated to that GO term in the protein list obtained by the presented PAL approaches. Source data are provided as a Source Data file.

Next, we tested the synthesized probes for PAL of proteins with HEK293T cell lysates. To this end, the Ap_*n*_A derivatives PALP-1 and PALP-2 were first incubated with HEK293T cell lysates. The samples were then irradiated with UV light (365 nm) to initiate the photo-crosslinking reaction and the labeled proteins were affinity-enriched via streptavidin-coated Sepharose^TM^ beads. After washing the beads thoroughly, the bound proteins were eluted with biotin. The eluted proteins were resolved by SDS-PAGE and subsequently analyzed by western blotting using ExtrAvidin®-Peroxidase. Aiming for high photo-crosslinking yields and selectivity at the same time, we optimized the experimental conditions with regard to the incubation and irradiation times as well as the concentrations of bait and competitor molecules (Fig. 3a and Supplementary Fig. 4).

For LC-MS/MS analysis of bound proteins, triplicates of PAL experiments with PALP-1 and PALP-2 were performed using HEK293T cell lysates. We conducted several control experiments including (i) experiments without UV irradiation, (ii) PAL experiments using con-1 as control substance, and (iii) experiments without addition of any PALPs to the cell lysates. After data processing (see below), only proteins that were enriched compared to these negative controls were included in our lists (Supplementary Data 1). In order to further limit the number of false positive hits, we conducted competition experiments using PALP-1 and PALP-2 in the presence of the respective unlabeled Ap_n_A analogs comp-1 and comp-2. For the final lists, only proteins that were enriched with PALP-1 and PALP-2 in the absence of comp-1 and comp-2, but not in their presence, were considered (Fig. 3c).

After affinity enrichment, proteins in the eluted fractions were resolved by SDS-PAGE and digested for LC-MS/MS analysis. Three replicates of this process were analyzed and measured as technical duplicates by LC-MS/MS to result in a total of six measurements per applied condition. PA-labeled proteins were identified by MaxQuant and quantified by LFQ^26,27^. The data was further analyzed by Perseus^28^ resulting in 732 identified proteins for PALP-1 and 802 identified proteins for PALP-2. For these proteins, missing value imputation (downshift 1.8, width 0.3) in the total matrix mode and multiple-sample tests (FDR ≤ 0.01, *S*_0_ = 0.2) were performed. Under these conditions, 56 proteins were identified as significant by ANOVA statistics for the PALP-1 experiment, 53 of which were significantly enriched after pairwise comparison to Tukey’s honest significant difference (THSD, FDR ≤ 0.05) and subsequent filtering against the three negative control experiments. These proteins were further filtered for significance against proteins enriched by the competitor control, resulting in 17 potential interaction partners for Ap_3_A (Fig. 3c). Usage of the Ap_4_A analog PALP-2 revealed 86 significantly enriched proteins by ANOVA of which 81 showed significant enrichment in PALP-2 towards the three negative control experiments by the *post hoc* THSD test (FDR ≤ 0.05). Of these proteins 26 showed significant enrichment against the competitor control (Fig. 3c).

Due to the low number of proteins identified with PALP-1, we decided to develop an additional PALP for Ap_3_A. Since *C*2-modifications in adenosines have been shown to be accepted by several nucleotide processing enzymes^20,29,30^, we synthesized (Supplementary Fig. 5) and applied PALP-3 (Fig. 3) as described above. With PALP-3, 538 proteins were identified, of which 341 were enriched against the negative controls, applying the same workflow as for PALP-1 and PALP-2. Comparison with the competitor experiments led to 49 significantly enriched proteins for PALP-3.

Proteins that were significantly enriched against all four controls after the ANOVA analysis (*S*_0_ = 0.2, FDR ≤ 0.01) followed by a Tukey’s *post hoc* THSD test were then visualized in heatmaps (Fig. 3; for heatmaps including proteins enriched by the control experiments see Supplementary Figs. 6 and 7). The color scale represents the median *Z*-score normalized LFQ value as calculated by Perseus. Several proteins identified by PALP-1 were also found with PALP-2 or PALP-3 (Supplementary Fig. 8).

When inspecting the distribution of identified proteins for the respective probes (Fig. 3d), it becomes evident that in addition to nine shared hits, our approach also revealed exclusive hits for the Ap_3_A probes (PALP-1 and PALP-3) and the Ap_4_A probe (PALP-2). Among those, we identified five aaRSs, which are known to play a major role in the synthesis of Ap_n_As^31^. Furthermore, inosine-5′-monophosphate dehydrogenase 2 (IMPDH2) was significantly enriched with PALP-3. In previous studies different variants of IMPDH have been shown to interact with Ap_n_As in binding-based and activity-based assays, indicating that this interaction plays a similar role in different organisms^32,33^.

The potential interaction partners for either Ap_3_A or Ap_4_A were further analyzed using bioinformatic tools. With the program Cytoscape/Biological Network Gene Ontology (BiNGO)^34^, enriched gene ontology (GO) terms were compared between the cohorts and the global proteome (Supplementary Data 2). We found for the proteins, which were enriched for Ap_3_A by applying PALP-1 and PALP-3, that 31% are involved in cellular response to chemical stimuli and 11% in response to oxygen levels (Fig. 3e). Both terms were not found to be enriched for the proteins identified with the Ap_4_A analog PALP-2. Regarding Ap_4_A, a total of 46% of the proteins that were identified with PALP-2 are involved in gene expression and 19 % in protein targeting (Fig. 3e). Both terms were not enriched in the proteins identified with PALP-1 or PALP-3. GO terms that were prevalent for both Ap_3_A-based and Ap_4_A-based probes, even though to a varying extent, include translation (13% in Ap_3_A-based and 35% in Ap_4_A-based probes) and tRNA aminoacylation (7% in Ap_3_A-based and 15% in Ap_4_A-based probes).

Notably, the majority of the identified proteins appear to be involved in metabolic processes ranging from those of carboxylic acids to nucleotides and carbohydrates (Fig. 3e and Supplementary Data 2). Exemplarily, proteins contributing to the metabolism of carboxylic acids were enriched in all our samples (41% for PALP-1 and PALP-3 and 38% for PALP-2). Another process that seems to be connected to both Ap_3_A and Ap_4_A is RNA processing. Taken together, 11 proteins (14%) were identified with the three PALPs that are associated with RNA processing, of which 7 (9%) are involved in mRNA splicing. In addition, several molecular functions were found to be enriched in our data sets (Supplementary Data 2). Interestingly, out of the 78 overall identified proteins, only 36 (46%) are known nucleotide binders whereof 25 (32%) are ATP binders. This indicates that Ap_3_A and Ap_4_A are not just ATP surrogates^2,32^ but have roles in cellular pathways that are distinguishable from those of other nucleotides.

### Ap_n_As are substrates for DcpS

**Fig. 4:**
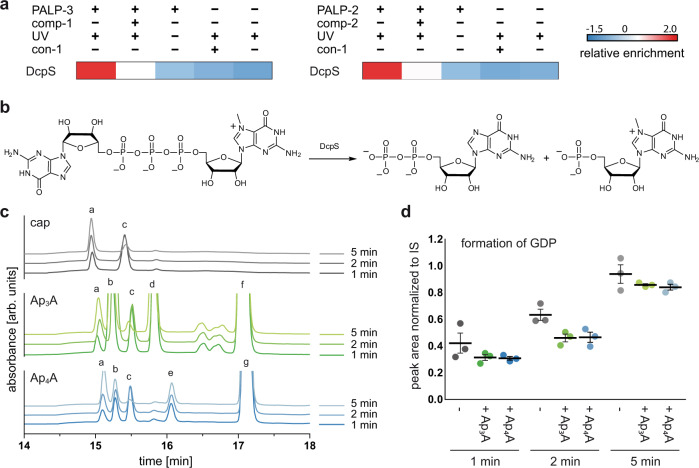
Investigation of the interaction of Ap_n_As with DcpS. **a** Extract of ANOVA-heatmap (*Z*-scores) for DcpS under different PAL conditions. **b** DcpS catalyzed cleavage of m^7^Gp_3_G. **c** Excerpt of the HPLC profiles for the hydrolysis of m^7^Gp_3_G (peak c) catalyzed by DcpS. The initial substrate concentration was 20 µM and the reactions were carried out in the absence (gray) or presence of Ap_3_A (green) and Ap_4_A (blue) (200 µM) at an enzyme concentration of 100 nM. Absorbance was measured at 260 nm (arb. units, arbitrary units). Formation of GDP (peak a) can be observed for all reactions. For Ap_3_A (peak f), ADP (peak b) and AMP (peak d) were identified as hydrolysis products. By analogy, AMP (peak d) and ATP (peak e) were identified as the cleavage products of Ap_4_A (peak g). The chromatographic peaks were identified by comparison with the retention times of reference samples and by subsequent MS analysis. **d** Quantification of the cleavage product GDP at different time points, applying adenosine as internal standard (IS). Data presented are mean ± SEM, *n* = 3 biologically independent experiments. Source data are provided as a Source Data file.

As mentioned above, 11 proteins involved in RNA processing were identified, including several ribonucleoproteins (HNRNPL, HNRNPA3, HNRNPA2B1 and HNRNPR) which were identified with the Ap_4_A-based PALP-2 and the decapping scavenger protein (DcpS, Fig. 4a) found with PALP-2 and PALP-3. DcpS belongs to the HIT family and is responsible for the very last step of mRNA degradation. Its main substrates are 5′-5′ linked dinucleoside triphosphates (m^7^Gp_3_N) that form the protective cap structure of eukaryotic mRNA^35,36^. The cap structure is selectively cleaved at the phosphate in proximity to m^7^G, yielding m^7^GMP and NDP (Fig. 4b).

To investigate the cleavage reaction of the cap analog m^7^Gp_3_G in the presence of Ap_3_A or Ap_4_A, an HPLC-based assay was developed. To this end, m^7^Gp_3_G was incubated with DcpS in the presence and absence of Ap_3_A or Ap_4_A. The progress of the reaction was monitored by HPLC (Fig. 4c and Supplementary Fig. 9). In addition to the expected cleavage products, ATP, ADP, and AMP were found in the reactions with Ap_n_A derivatives, demonstrating that both Ap_3_A and Ap_4_A can be cleaved by DcpS. Quantification of GDP at different time points showed that cleavage of m^7^Gp_3_G was to some extent impaired when incubated with either Ap_3_A or Ap_4_A (Fig. 4d).

### UBA1 accepts Ap_4_A as ATP surrogate but not Ap_3_A

**Fig. 5:**
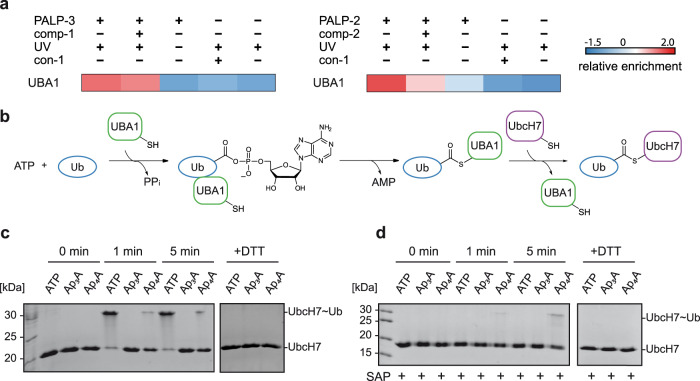
Investigation of the interaction of Ap_n_As with UBA1. **a** Extract of ANOVA-heatmap (*Z*-scores) for UBA1 under different PAL conditions. **b** ATP-dependent Ub activation and transthioesterification reactions catalyzed by UBA1. **c** Detection of Ub transfer from UBA1 to UbcH7 in the presence of Ap_3_A, Ap_4_A or ATP, analyzed by non-reducing SDS-PAGE (15%) followed by Coomassie blue staining. **d** The nucleotides were incubated with SAP before performing the transthioesterification reaction to remove possible contaminants of ATP in the Ap_3_A and Ap_4_A samples. The experiments were performed twice with matching results. Source data are provided as a Source Data file.

Ubiquitin-like modifier-activating enzyme 1 (UBA1) was among the most significantly enriched hits for Ap_4_A (PALP-2), whereas it was less enriched with Ap_3_A (PALP-1 and PALP-3, Fig. 5a). UBA1 catalyzes the first reaction of the ubiquitin (Ub) conjugation cascade, namely the activation of Ub^37^. In this reaction, Ub is first adenylated at its carboxyl terminus in an ATP-dependent manner and then transferred to a conserved cysteine residue of UBA1 via thioester bond formation. Finally, Ub is transferred to an E2 enzyme by a transthioesterification reaction (Fig. 5b).

As it has already been reported that UBA1 is able to synthesize Ap_4_A in vitro^7^, we investigated if Ap_3_A and Ap_4_A can substitute for ATP in the Ub cascade. Therefore, the UBA1-catalyzed activation of Ub and the consecutive transfer to the E2 enzyme UbcH7 was investigated as a model system. By incubating the enzyme mix with Ap_4_A in the absence of ATP, the formation of a UbcH7~Ub thioester complex was observed after 1 and 5 min by non-reducing SDS-PAGE analysis, whereas this was not observed for Ap_3_A (Fig. 5c). To confirm that the effect derives from Ap_4_A itself and not from potential contamination with ATP, the different nucleotides were pre-treated with shrimp-alkaline phosphatase (SAP, Fig. 5d). As expected, Ub activation and, thus, UbcH7~Ub thioester complex formation was not observed for ATP, while it was detected when Ap_4_A was employed.

### LDHA and PGK1 bind to a fluorescent labeled Ap_3_A analog

**Fig. 6:**
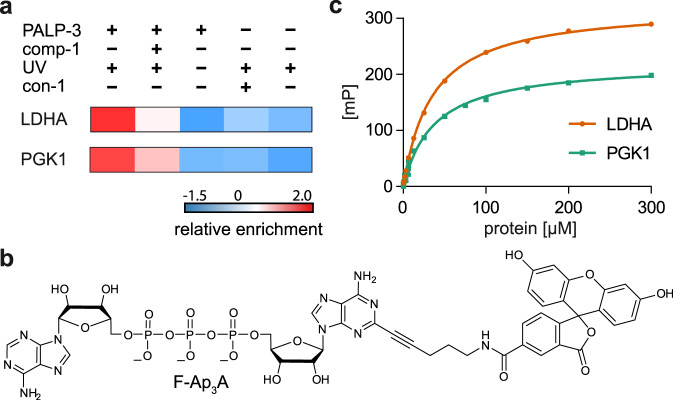
Investigation of the binding affinity of LDHA and PGK1 to the fluorescent labeled Ap_3_A analog F-Ap_3_A via fluorescence polarization (FP). **a** Extract of ANOVA-heatmap (*Z*-scores) for LDHA and PGK1 under different PAL conditions. **b** Chemical structure of F-Ap_3_A. **c** FP-based quantification of binding between LDHA (orange) or PGK1 (green) to F-Ap_3_A. Increasing concentrations of protein were incubated with F-Ap_3_A (25 nM) for 15 min on ice and 30 min at 30 °C before measurement. FP values were determined and plotted against the protein concentrations. Data presented are mean ± SD, *n* = 3 technical replicates. Source data are provided as a Source Data file.

As mentioned before, the majority of the proteins enriched with the used PALPs are involved in metabolic processes. Among the most prominent hits were LDHA (found for all PALPs) and PGK1 (found with PALP-3), which showed strong enrichment in our PAL experiments compared to the controls (Fig. 6a). Additionally, recent reports document that besides their functions as (post-)glycolytic enzymes, both proteins are reported to have non-metabolic functions including involvement in tumorigenesis and stress response^38,39^. To investigate the interactions between these proteins and Ap_*n*_As, we developed a method to measure binding affinities via fluorescence polarization (FP). Based on the principle that the tumbling rate of a small fluorescently labeled molecule decreases upon binding to a larger interaction partner, which is mirrored in an increase in FP, FP assays present a well-established method to quantify such interactions^40,41^. First, we synthesized a fluorescein-labeled Ap_3_A analog (F-Ap_3_A) that similar to PALP-3, bears a 5-carboxyfluorescein dye linked to the *C*2 position of one adenine moiety (Fig. 6b).

F-Ap_3_A was then used to measure binding affinities with LDHA and PGK1. We incubated increasing concentrations of these proteins with F-Ap_3_A and plotted the obtained FP values against the protein concentration (Fig. 6c). This revealed that LDHA and PGK1 bind to F-Ap_3_A with *K*_D_ values of 35.27 ± 0.69 µM and 37.39 ± 1.62 µM, respectively (for further details, see Supplementary Table 1).

### Proteomic profiling of the Ap_3_A and Ap_4_A protein interactome in *E. coli*

**Fig. 7:**
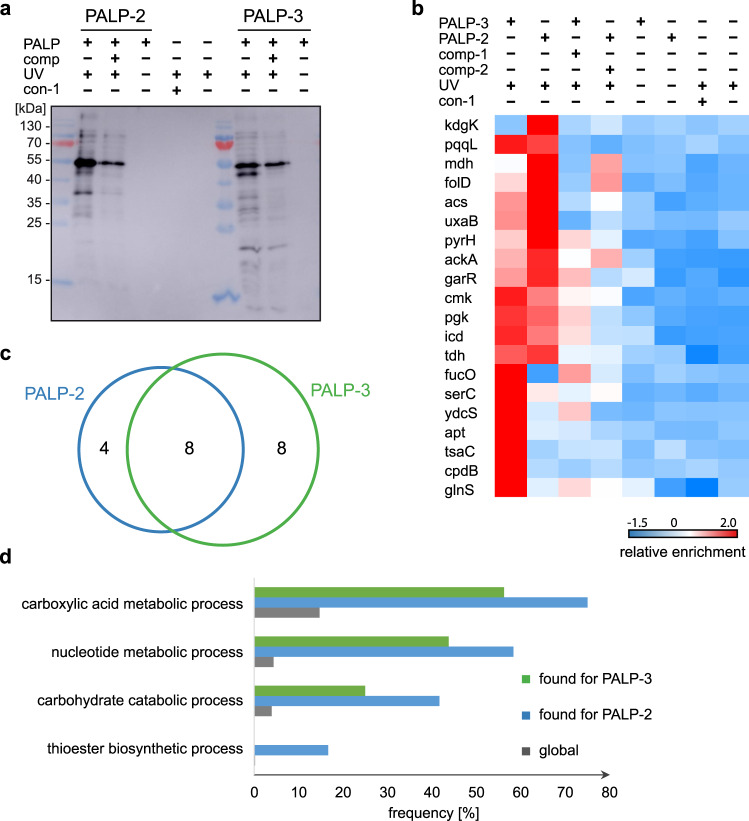
Proteomic analysis of PAL experiments using *E. coli* cell lysates. **a** Western blot analysis with ExtrAvidin®-Peroxidase showing enrichment of labeled proteins under optimized conditions using PALP-2 and PALP-3 as indicated after irradiation with UV light (365 nm) for 5 min. **b** Heatmap representation (*Z*-scores) of proteins that were quantified in the respective experiments in at least four out of six measurements and that passed a one-way ANOVA-based multiple-sample test for statistically significant enrichment, with a permutation-based false discovery rate (FDR) below or equal to 0.01 and *S*_0_ value set to 0.2, followed up by two-sided *post hoc* Tukey’s HSD test (FDR ≤ 0.05) to determine proteins enriched against all performed control experiments. **c** Venn diagram illustrating the overlap of identified proteins with PALP-3 (green) and PALP-2 (blue). **d** Frequencies of the enriched GO terms are compared between PALP-3 (green) and PALP-2 (blue) and the global *E. coli* proteome (gray) according to GO analysis using a one-sided hypergeometric test performed with the Cytoscape BiNGO resource (Supplementary Data 4). Global frequency represents the number of genes annotated to a GO term in the entire *E. coli* proteome, while sample frequency represents the number of genes annotated to that GO term in the protein list obtained by PAL. Source data are provided as a Source Data file.

Next, we investigated the Ap_n_A interactome of the *E. coli* K12 strain by applying the PAL approach using PALP-2 and PALP-3 (Fig. 7a). The data obtained by LC-MS/MS was analyzed as described above resulting in the identification of a total of 46 proteins significantly enriched against the three negative control experiments, of which 20 were significantly enriched against the respective competitor controls. Significance was determined as described before by ANOVA statistics (FDR ≤ 0.01, *S*_0_ = 0.2) and subsequent analysis of the hits by THSD test (FDR ≤ 0.05). The proteins identified and their *Z*-score normalized median LFQ values measured in the different conditions were displayed in a combined heatmap (Fig. 7b, Supplementary Data 3 and Supplementary Fig. 10). Interestingly, the distribution of the hits retrieved with the Ap_3_A-based and Ap_4_A-based probes with *E. coli* K12 lysates was similar to the one obtained with eukaryotic HEK293T cells. GO analysis showed significant enrichment for various metabolic pathways, including carboxylic acid metabolism (more than 50% in both cases), nucleotide metabolism (more than 40% in both cases), and carbohydrate catabolism (more than 20% in both cases) (Fig. 7d and Supplementary Data 4). Also, out of the 20 identified hits, 11 (55%) are known nucleotide binders and 8 (40%) are known ATP binders. Noteworthy, 40% of the hits are overlapping for PALP-2 and PALP-3 probes (Fig. 7c), which is significantly higher than for the proteins identified from HEK293T cell lysates, where only 12% were overlapping.

We finally investigated the human orthologues of the bacterial hits using the PANTHER (Protein ANalysis THrough Evolutionary Relationship) bioinformatics resource^42^. Two of the most prominent hits, enriched in PAL experiments using HEK293T and *E. coli* lysates, were the phosphoglycerate kinase, encoded by the *pgk* gene in bacteria and by *PGK1* in humans, and the malate dehydrogenase, encoded by *mdh* in bacteria and *MDH1* and *MDH2* in humans. Interestingly, phosphoglycerate kinase and malate dehydrogenase are both involved in carbohydrate metabolism, as well as LDHA and GALK1 that we identified in HEK293T cell lysates, highlighting potential common roles of Ap_n_As in bacteria and eukarya. Furthermore, the bacterial orthologue of the aaRS QARS (*glnS*) was found, showing further similarities in our results.

## Discussion

Even though Ap_3_A and Ap_4_A were discovered in the 1960s^1^, their cellular roles and functions remain enigmatic. Here, we report proteome-wide interaction maps of Ap_3_A and Ap_4_A by employing probes for PAL in combination with MS-based proteomics. In comparison to previous affinity enrichment studies^32,43,44^ that relied on non-covalent interactions between Ap_n_As and potential binders, our study is based on covalently connecting Ap_3_A-based and Ap_4_A-based probes to their target proteins allowing for both trapping transient interactions and thorough washing steps in order to minimize false positive hits. Given the relatively small size of the chemical modifications in the PALPs in comparison to the dimensions of an average protein also renders covalent “trapping” of indirect interaction partners of Ap_n_A unlikely, even though it cannot fully be excluded.

The earlier studies^32,43,44^ exclusively investigated Ap_4_A and mostly *E. coli* cells. Albeit less rigorous investigations and analysis were conducted (i.e., fewer repetitions and less rigorous statistical analysis), only 6–13 proteins were identified. Furthermore, there is little overlap in identified proteins between these studies and compared to our studies.

Overall, we identified 78 proteins from HEK293T cell lysates that potentially interact with Ap_3_A and Ap_4_A after rigorous statistical analysis. Only about half of these are known nucleotide binding proteins, indicating a broader scope of the roles of Ap_3_A and Ap_4_A in cellular processes beyond nucleotide-processing and speaking against the hypothesis that Ap_n_As merely act as ATP-surrogates^32^. In fact, our chemical proteomics study uncovered interactions that are specific for either Ap_3_A or Ap_4_A and interactions that are shared by both Ap_n_As, again pointing towards broader and specific roles of the respective nucleotides.

The majority of the proteins found to interact with Ap_3_A and Ap_4_A probes appear to be involved in metabolic processes ranging from those of carboxylic acid to nucleotides and carbohydrates (Fig. 3e and Supplementary Data 2). Besides these common interactions, Ap_3_A appears to play a role in cellular response to e.g., chemical stimuli and changing oxygen levels, which may correlate to the observed changes in Ap_3_A levels in HEK293T cell lysates upon stress induced by mitomycin C and H_2_O_2_. For Ap_4_A we identified a variety of proteins involved in gene expression, translation and protein targeting, as well as UBA1, an essential enzyme of the Ub system, whose interaction with Ap_4_A we could demonstrate in a case study (Fig. 5)^37^. Taken together, our results support the hypothesis that Ap_3_A and Ap_4_A are alarmones that signal various stress conditions^2,45–47^.

Our PAL studies also discovered the involvement of Ap_3_A and Ap_4_A in RNA-associated processes. Proteins involved in tRNA aminoacylation and mRNA splicing are overrepresented among the proteins found with either probe. We also identified several proteins that are connected to regulation of translation. This is particularly interesting since recent studies report on the involvement of dinucleoside polyphosphates in RNA cap formation in bacteria. Luciano et al. showed that Np_4_As can be used as precursors for RNA cap structures that serve as primers for the *E. coli* RNA polymerase^48^. Furthermore, Hudeček et al. reported that both Ap_3_A and Ap_4_A can be incorporated into bacterial RNA caps^49^. Likewise, our PAL study identified the decapping scavenger enzyme DcpS as Ap_n_A-binder. Using additional follow-up assays, we demonstrated that Ap_3_A and Ap_4_A can act as alternative substrates for the latter (Fig. 4), and thereby slow down the cleavage of cap analogs. All these results point at a potential role of these Ap_*n*_As in the regulation of RNA stability, metabolism, and/or transport.

Investigations of *E. coli* K12 cells revealed a total of 20 target proteins for Ap_3_A and/or Ap_4_A. Again, only about half of the identified proteins were known nucleotide binders, comparable to the results obtained for HEK293T cells. Moreover, similar to the results obtained with HEK293T cells, proteins involved in various metabolic pathways, including carboxylic acid metabolism, nucleotide metabolism, and carbohydrate catabolism were found to be enriched using *E. coli* extracts indicating potentially conserved functions of these Ap_n_As in bacterial and eukaryotic cells. Two metabolic enzymes identified from *E. coli* lysates were the phosphoglycerate kinase and the malate dehydrogenase, proteins whose human orthologues we also found in HEK293T. Interestingly, both enzymes are involved in carbohydrate metabolism, which is the case for several proteins we identified in *E. coli* and HEK293T, such as pgk, mdh, PGK1, MDH2, LDHA, and GALK1. Recent reports showed that various metabolic enzymes including PGK1, LDHA, and MDH2 have non-metabolic functions in processes including tumorigenesis and stress response, making them interesting candidates for follow-up experiments^35,36^. To further investigate the interaction between Ap_n_As and these proteins, we measured binding affinities between PGK1 and LDHA and a fluorescein-labeled Ap_3_A probe and obtained *K*_D_ values in the 10^−5^ M range.

Overall, our approach based on PAL experiments with cell extracts combined with proteomics provides proteome-wide interaction maps for Ap_3_A and Ap_4_A in eukaryotic and bacterial cells. Still the proteins we identified as potential interaction partners of Ap_3_A and Ap_4_A likely represent only a subset of their full interaction networks. One piece of evidence supporting this assumption is that the use of the differently modified Ap_3_A-based probes in PALP-1 and PALP-3 resulted in overlapping but also distinct hits, indicating a substrate bias that is inherent in the use of such chemical probes. This might also be the reason why several known interactors such as NUDT2 and FHIT were not identified. However, the applied control experiments and the rigorous statistics that we used for evaluation make it likely that significantly enriched proteins are also true positive interactors. Nonetheless, already at the present state, these maps represent a rich source that will hopefully spur and guide further investigations aimed at the elucidation of the hitherto enigmatic roles and functions of these nucleotides. Moreover, the Ap_*n*_A-PAL approach presented here opens up an avenue to discover the function and roles of these Ap_*n*_As e.g., by applying these PALPs and eventually new PALP scaffolds to different cell types exposed to external cues.

## Methods

Chemical synthesis and characterization of compounds are described in the Supplementary Methods.

### Cell culture

#### Growth conditions for human cells

HEK293T cells (ATCC) were grown in Dulbecco’s modified Eagle medium (DMEM) supplemented with 10% (v/v) fetal calf serum (FCS) and 100 units/mL penicillin/streptomycin at 37 °C and 5% CO_2_. Cells were passaged every 2–3 days. For PAL experiments only passages up to 20 were used.

#### Growth conditions for *E. coli* K12 BW25113

Bacterial cells were grown in LB medium at 37 °C and 200 rpm overnight. For the main culture, LB medium was inoculated to a calculated OD_600_ of 0.01 with the overnight culture and grown for 5 h at 37 °C to reach the late exponential growth phase. Cells were harvested by centrifugation (3220×*g*) for 5 min.

### Quantification of intracellular Ap_3_A and Ap_4_A in HEK293T cells under stress conditions

#### No external stress

At 90% confluence, the cell dish was directly put on ice. The medium was removed and the cells were washed twice with 2 mL of ice-cold isotonic NaCl solution. One milliliter of −80 °C lysing solution (90/9/1 H_2_O/MeOH/CHCl_3_ (v/v/v)), as well as the internal standard solution (20 µl of 5 µM ^13^C_10_-Ap_3_A and ^13^C_10_-Ap_4_A in water) was added to the cells and distributed evenly. After 15 min on ice, the lysed cells were scraped down and collected. The plate and the scraper were washed with 1 mL of lysing solution and the suspensions were combined. After centrifugation (21,882×*g*, 4 °C, 30 min) the supernatant was stored on ice and the pellet was ultrasonicated in 500 µL lysing solution (5 min) and centrifuged again (21,882×*g*, 4 °C, 30 min). The combined supernatants were evaporated in a SpeedVac Vacuum Concentrator (Thermo Fisher Scientific) and the residue was stored at −20 °C until further purification.

#### DNA damage via mitomycin C (MMC)

At 90% confluence of the cells, MMC in 1× PBS was added to the dish to result in a final concentration of 100 nM MMC in 10 mL medium. The cells were incubated for 18 h at 37 °C. After the treatment, the cells were treated in the same way as the unstressed samples.

#### Oxidative damage via hydrogen peroxide

At 90% confluence, the medium was removed, the cells were washed two times with 1× PBS and 5 mL of a 500 µM H_2_O_2_ solution in 1× PBS was added. The cells were incubated for 20 min at 37 °C. The resulting cell suspension was transferred to a 15 mL falcon, the plate was washed with 4 mL 1× PBS and the suspensions were combined and centrifuged (500×*g*, 4 °C, 3 min). The cell pellet was resuspended in 1 mL of −80 °C lysing solution and after 20 min of incubation on ice, the suspension was centrifuged (3220×*g*, 4 °C, 5 min). The supernatant was transferred in an Eppendorf tube and the pellet was resuspended and ultrasonicated in 1 mL lysing solution. After centrifugation (3220×*g*, 4 °C, 5 min) the supernatants were combined and evaporated in a SpeedVac Vacuum Concentrator (Thermo Fisher Scientific) and the residue was stored at −20 °C until further purification.

#### Purification via tC18 columns

The SepPak® tC18 column (Waters) was washed with 1 mL MeCN and equilibrated with 2 mL 5% MeCN in water. The cell extract was dissolved in 500 µL 5% MeCN in water and applied to the column. The eluted solution was collected, the column was washed with 1 mL 5% MeCN in water and the combined extracts were freeze-dried. The resulting residue was dissolved in 50 µL water and directly measured via LC-HR-ESI-MS.

#### Quantification of intracellular Ap3A and Ap4A

The purified cell extract was analyzed and quantified via LC-HR-ESI-MS (Bruker MicrOTOFII). A Hypercarb Porous Graphitic Carbon LC column (5 µm, 100 × 2.1 mm) was used in combination with a Hypercarb drop-in guard precolumn (5 µm, 10 × 2.1 mm). As mobile phase, 10 mM NH_4_OAc + 0.1% diethylamine (pH 10) (A) and MeCN (B) was used (gradient shown in Supplementary Table 2). For the calibration curve, different volumes of known concentrations of Ap_3_A and Ap_4_A were injected and linearly fitted. In every measured calibration sample, the same amount of internal standard (^13^C_10_-Ap_3_A and ^13^C_10_-Ap_4_A) was present. The calibration curve was renewed for every experiment. To measure technical duplicates, every sample was injected twice (20 µL each). For the calculation of the concentrations, the EICs of Ap_3_A and Ap_4_A (*m*/*z* = 755.0747 ± 0.02 and 835.0410 ± 0.02) were integrated and quantified. For the internal standard, the EICs of ^13^C_10_-Ap_3_A and ^13^C_10_-Ap_4_A (*m*/*z* = 765.1082 ± 0.02 and 845.0740 ± 0.02) were integrated and quantified. The resulting calculated concentrations of the technical duplicates were averaged. Exclusively measurements with a *S*/*N* bigger than *S*/*N* = 7 were considered for the calculation of concentrations. In case of insufficient spectrum quality or *S*/*N* ratio of one of the duplicates only the duplicate of sufficient quality was considered for the calculation. The quantification of the samples and the standardization of the concentrations with the aid of the internal standards was done via Bruker Compass DataAnalysis (version 4.1) and Bruker Compass QuantAnalysis (version 2.1). Statistical tests were done using a one-way ANOVA in combination with the Dunnett test in GraphPad PRISM (version 6.01).

### Cell lysate stability assay

The stability of Ap_3_A and Ap_4_A in HEK293T cell lysate was measured by RP-HPLC. To this end, 200 μM nucleotides were incubated with or without 2 mg/mL HEK293T cell lysates in 20 mM HEPES (pH 7.4), 100 mM NaCl, 5 mM MgCl_2_, and 1 mM DTT at 37 °C for 1 h in a volume of 45 μL. 15 μL aliquots were taken at 0 and 60 min followed by the removal of proteins and salts by U-C4 ZipTips (Merck Millipore). The resulting mixture was diluted with 100 μL water. 110 μL sample was injected and analyzed by analytical RP-HPLC with a 250/4 mm NUCLEODUR C18 HTec column. The run was performed with 50 mM aqueous triethylammonium acetate buffer (TEAA, pH 7.0) and MeCN as eluents (gradient shown in Supplementary Table 3).

### PAL experiments

#### Preparation of HEK293T cell lysate

Cells were grown to 90% confluence. Before lysis, cells were centrifuged at 500×*g* at 4 °C for 10 min. Cell pellets were washed with cold 1× PBS (3 × 2 mL), resuspended in cold lysis buffer (25 mM Tris∙HCl, pH 7.5, 150 mM NaCl, 1 mM DTT, 1 mM EDTA, 1% NP40, 5% glycerol, 0.1 M Pefabloc, 1 µg/mL aprotinin/leupeptin) and sonicated on ice at an amplitude of 10% for 10 s, three times. Lysates were centrifuged at 4 °C and 19,064×*g* for 30 min. The protein content of the supernatant was determined by BCA assay (Kit, Thermo Fisher Scientific). The supernatant was stored on ice until further use.

#### Preparation of *E. coli* K12 cell lysate

Pellets were stored on ice and washed with cold 1× PBS (10 mL). After repeating steps of centrifugation and washing in total of three times, the pellet was resuspended in cold lysis buffer (25 mm Tris∙HCl, pH 7.5, 150 mM NaCl, 1 mM DTT, 1 mM EDTA, 1% NP40, 5% glycerol, 0.1 M Pefabloc, 1 µg/mL aprotinin/leupeptin). The cell mixture was sonicated on ice at an amplitude of 20% for 30 s, five times. The mixture was centrifuged at 4 °C and 19,064×*g* for 30 min. The protein concentration in the supernatant was determined by BCA assay (Kit, Thermo Fisher Scientific). The supernatant was stored on ice until further use.

#### Photoaffinity labeling

In a total volume of 100 µL the photoaffinity-labeling probe (PALP-1, PALP-2, or PALP-3, 20 µM) was incubated with lysate (3.0 mg/mL) in HEPES buffer (50 mM, 5 mM MgCl_2_, pH 6.8) for 2 h on ice in a 96-well plate (Sarstedt) under protection from light. Afterwards, the well plate was positioned on ice under an UV lamp (365 nm, distance approx. 6 cm, 8 mW/cm^2^) for 5 min. The mixture was incubated with streptavidin Sepharose^TM^ high performance (GE Healthcare) beads (35 µL, 300 nmol/mL) for 60 min at room temperature. The streptavidin beads were washed with 1× PBS (3 × 200 µL) and centrifuged (room temperature, 272×*g*, 3 min) before use. After incubation, the samples were centrifuged (room temperature, 272×*g*, 3 min) and the supernatant was removed. The beads were washed with 1× PBS containing 0.4% SDS (3 × 100 µL), aqueous urea solution (6 M, 2 × 100 µL) and 1× PBS (3 × 100 µL) and centrifuged (room temperature, 272×*g*, 2 min). After removing the supernatant, elution was performed with an aqueous biotin solution (0.4 mM, 2 × 40 µL) and water (1 × 40 µL) at 37 °C for 10 min. Elution fractions were collected, freeze-dried, diluted in water and loading dye (6 ×, 225 mM Tris∙HCl (pH 6.8), 50% glycerol, 12.5% β-mercaptoethanol, 4.5% SDS, 0.05% bromophenol blue), heated for 5 min to 95 °C and subjected to SDS-PAGE and western blot analysis using ExtrAvidin®-Peroxidase (Sigma-Aldrich).

#### Control experiments

The control samples were treated as described above. In case of the UV control, the sample was handled in a black Eppendorf tube to exclude UV irradiation. In the beads control water was added instead of the respective PALPs. The control substance (con-1) was applied in the same concentration as the PALPs (20 µM). For the competition experiment a 100-fold excess of the competitor (comp-1 or comp-2, respectively, 2 mM) derivative was incubated with cell lysate for 1 h on ice before adding the corresponding PALPs.

#### In-gel digestion

For in-gel digestion each gel lane was cut into small pieces and destained repetitively in acetonitrile/water (3:2, v/v, 50 µL) for 30 min and NH_4_HCO_3_ (25 mM, 50 µL) for 15 min. Then, the gel pieces were incubated with NH_4_HCO_3_ (20 mM, 50 µL) for 15 min. After washing, proteins were reduced with DTT (10 mM in NH_4_HCO_3_, 20 mM, 100 µL) for 60 min at 56 °C, followed by alkylation with iodoacetamide (50 mM in NH_4_HCO_3_, 20 mM, 100 µL) at room temperature for 60 min under protection from UV light. After washing in NH_4_HCO_3_ (20 mM, 100 µL) for 15 min and in NH_4_HCO_3_/acetonitrile (20 mM, 1:1, v/v, 100 µL) for 10 min and dehydration in pure acetonitrile (100 µL) for 10 min, proteins were digested overnight at 37 °C with trypsin (1:50, w/w) (Promega V5111). Peptides were extracted twice from the gel with acetonitrile/TFA in water (0.1%, 1:1) for 60 min. After desalting using ZipTips (Merck Millipore) the peptides were subjected to mass spectrometric analysis with nano-LC-MS/MS (Proteomics facility, University of Konstanz).

#### Mass spectrometry

The digests were analysed on a QExactive HF mass spectrometer (Thermo Fisher Scientific, Bremen, Germany) operated with Tune (version 2.9) and interfaced with an Easy-nLC 1200 nanoflow liquid chromatography system (Thermo Fisher Scientific, Bremen, Germany). The peptide digests were reconstituted in 0.1% formic acid and loaded onto the analytical column (50 μm × 15 cm). Peptides were resolved at a flow rate of 300 nL/min using a linear gradient of 6–40% solvent B (0.1% formic acid in 80% acetonitrile) over 45 or 60 min. Data-dependent acquisition with full scans in a 350−1200 m/z range was carried out at a mass resolution of 120,000. The 20 most intense precursor ions were selected for fragmentation. Peptides with charge states 2–7 were selected and dynamic exclusion was set to 30 s. MS2 scans were carried out at a mass resolution of 15,000. Precursor ions were fragmented using higher-energy collision dissociation (HCD) set to 28%. Each of the three independent biological replicates was measured as technical duplicates.

#### Data analysis and quantification

Raw files from LC-MS/MS measurements were analyzed using MaxQuant (version 1.6.2.6) with the andromeda search engine with default settings and match between runs and label-free quantification (LFQ) (minimum ratio count 2) enabled^26,27^. For protein identification, the human and the *E. coli* reference proteome downloaded from the UniProt database (https://www.uniprot.org/proteomes/) (download date: 02.04.2019 and 01.10.2019) and an integrated database of common contaminants were used. Oxidation, *N*-acetylation, and carbamidomethylation were selected as modifications. Identified proteins were filtered for reverse hits, common contaminants and hits only identified by site at a false discovery rate (FDR) of equal or below 0.01. Further data processing was performed using Perseus software (version 1.6.14.0)^28^. LFQ intensities were log_2_ transformed. The proteins were filtered to be detected in at least 60% of the replicate experiments (*n* = 6) and missing values were imputed from a normal distribution (width = 0.3 and shift = 1.8) in the total matrix mode, based on the assumption that these proteins were below the detection limit. Enriched proteins were identified by a one-way ANOVA-based multiple sample test with *S*_0_ adjusted to 0.2, the number of randomizations set to 250 and the permutation-based FDR accepted equal or below 0.01 with technical replicates grouped for randomization. A two-sided Tukey’s *post hoc* test with an accepted FDR value equal or below 0.05 was performed on all ANOVA significant hits. The Tukey’s Honest Significant Difference (THSD) performs a pairwise comparison of all conditions and calculates the mean difference between each condition pair. If the difference is greater than or equal to the corresponding THSD, which depends on the number of treatments, the degrees of freedom, the mean squared error and the number of data points in each group, the difference is considered significant. For further analysis only proteins enriched in a PAL experiment against all four corresponding controls were considered. *Z*-scoring of the median LFQ intensities was performed without grouping and used for unsupervised hierarchical clustering of all significant hits, the distance was set to Euclidian, the linkage to average, and the maximal numbers of clusters to 300.

#### GO term and abundance analysis

The obtained protein hits were analyzed using Cytoscape (version 3.8.2) with the BiNGO app (version 3.0.3) to compare the abundance of GO terms in the identified proteins to their frequency in the whole proteome. A one-sided hypergeometric test was performed with a significance level of 0.05 and Benjamini Hochberg False Discovery Rate (FDR) (looks at the frequency with which that GO term appears, the *p*-value consequently falls as the frequency increases)^50^. Global frequency is the number of genes annotated to a GO term in the entire background set, while sample frequency is the number of genes annotated to that GO term in the input list. (cf. http://geneontology.org/docs/go-enrichment-analysis/). Human and *E. coli* gene association (.gaf) and ontology files (.obo) were downloaded from geneontology.org (http://geneontology.org) (27.01.2020). For the full output of the performed analysis, see Supplementary Data 2 and 4. Human orthologues of the bacterial hits were investigated using PANTHER (version 16.0)^42^ bioinformatics resource.

### Biochemical validation experiments

#### Expression and purification of human DcpS^51^

The protein expression plasmid pET28b containing the sequence for His-tagged DcpS was kindly provided by A. Rentmeister (Institute of Biochemistry, WWU Münster). After transformation into BL21 (DE3) *E. coli* cells, the cells were grown in LB medium containing 100 mg/L kanamycin at 37 °C overnight. The pre-culture was diluted with medium containing 100 mg/L kanamycin to an OD_600_ value of 0.1. Cells were grown at 37 °C until they reached an OD_600_ of approximately 1.1. Protein expression was induced by addition of 1 mM IPTG at 25 °C for 4 h. Then, cells were pelleted, resuspended in lysis buffer (50 mM Tris∙HCl (pH 8.0), 350 mM NaCl, 20% sucrose, 20 mM imidazole, 1 mM β-mercaptoethanol) and lysed by sonication at an amplitude of 20% for 45 s, five times. The lysate was clarified by high speed centrifugation and purified by immobilized metal ion affinity chromatography (IMAC) using a HisTrap^TM^ FF crude column on an ÄKTA system. The protein was eluted in lysis buffer, with linear increasing concentrations of imidazole (12.5–250 mM). The pooled fractions were further purified by size exclusion chromatography (Sephadex G-25) using 10 mM Tris∙HCl (pH 8.0), 25 mM NaCl, 1 mM DTT as mobile phase. Protein purity was analyzed by 12.5% SDS-PAGE followed by Coomassie blue staining, and the concentration was determined by UV absorption (Nanodrop ND-1000, Peqlab). The protein was stored at −80 °C until use.

#### Investigation of m^7^Gp_3_G cleavage by DcpS in the presence of Ap_3_A and Ap_4_A

Ap_3_A and Ap_4_A (Jena Bioscience) were diluted in DcpS assay buffer (10 mM Tris∙HCl (pH 7.5), 200 mM KCl, 0.5 mM EDTA, 1.0 mM DTT)^52^ to a final concentration of 200 µM in 150 µL. DcpS was added and the mixture was incubated at 30 °C for 15 min. The cap analog was added and the mixture was incubated for up to 1 h. Aliquots were taken at the indicated time points and the enzymatic reaction was stopped by denaturing DcpS for 2.5 min at 97 °C. 7 µL of 10% TFA were added, as well as 1 µL of a 1 mM adenosine solution as internal standard. The protein was removed with U-C18 ZipTips (Merck Millipore), the mixture was freeze dried and then dissolved in water for analysis by RP-HPLC using a Pyramid column. Samples without Ap_3_A or Ap_4_A were treated the same way. All experiments were performed three times as biological replicates. Peak areas were determined using LabSolutions Shimadzu (version 5.71) and normalized by the adenosine peak. Peak areas for the cleavage product (GDP) were compared using GraphPad PRIMS (version 6.01) and all new peaks were collected and further characterized by ESI-MS.

#### E2 charging assay

Human N-terminal His-tagged ubiquitin-like modifier activating enzyme 1 (UBA1) was expressed in *E. coli* BL21 RIL and purified via IMAC and size exclusion chromatography^53^. The C-terminally His-tagged E2 enzyme UbcH7 was expressed in *E. coli* BL21 (DE3) and purified via IMAC^54^. Ub was expressed in *E. coli* BL21 (DE3) and purified via IMAC and cation exchange chromatography^7^.

The E2 charging assay was performed with 250 nM UBA1, 4 µM UbcH7, 15 µM Ub, 25 mM Tris·HCl (pH 7.5), 50 mM NaCl, 0.1 mM DTT, 10 mM MgCl_2_, and started with 0.2 mM of the indicated nucleotide (ATP, Ap_3_A, or Ap_4_A). For the SAP control, the nucleotides were pre-incubated with 0.5 units of shrimp alkaline phosphatase (rSAP) (New England Biolabs) at 37 °C for 30 min, followed by inactivation of the rSAP at 65 °C for 15 min. The reaction mixtures were incubated at 20 °C and enzymatic reactions were stopped at the indicated time points by the addition of 6× non-reducing SDS loading dye. To the DTT controls 1 µL of 1 M DTT was added before stopping the reaction. Samples were separated by 15% SDS-PAGE gels and stained with Coomassie blue.

#### Expression and purification of human LDHA

The codon optimized cDNA of human LDHA (Thermo Fisher Scientific) was cloned into the pET21a vector using NdeI and XhoI restriction sites to generate pET21a-LDHA. The plasmid was transformed into BL21 (DE3) *E. coli* cells, which were then grown in LB medium containing 100 mg/L carbenicillin at 37 °C overnight. The pre-culture was diluted with LB medium containing 100 mg/L carbenicillin to an OD_600_ value of 0.1. Cells were grown at 37 °C until they reached an OD_600_ of approximately 0.6. Protein expression was induced by addition of 1 mM IPTG at 25 °C for 4 h. Then, cells were pelleted, resuspended in lysis buffer (20 mM HEPES (pH 7.5), 200 mM NaCl, 1 mM β-mercaptoethanol, 1 % Triton™ X-100, 0.1 M Pefabloc, 1 µg/mL aprotinin/leupeptin) and lysed by sonication at an amplitude of 20% for 30 s, three times. The lysate was clarified by high speed centrifugation and purified by immobilized metal ion affinity chromatography (IMAC) using a HisTrap^TM^ FF crude column (1 mL, GE Healthcare) on an ÄKTA system. The protein was eluted in 20 mM HEPES (pH 7.5), 200 mM NaCl, 1 mM β-mercaptoethanol, with linear increasing concentrations of imidazole (30–500 mM). Fractions containing pure LDHA protein were identified by 12.5% SDS-PAGE followed by Coomassie blue staining and pooled. The elution buffer was replaced by LDHA storage buffer (20 mM HEPES (pH 7.5), 200 mM NaCl, 10% glycerol) via dialysis overnight. The protein concentration was determined by BCA assay (Kit, Thermo Fisher Scientific). The protein was stored at −20 °C until use.

#### Expression and purification of human PGK1

The codon optimized cDNA of human PGK1 (Thermo Fisher Scientific) was cloned into the pET21a vector using NdeI and XhoI restriction sites to generate pET21a-PGK1. The plasmid was transformed into BL21 (DE3) *E. coli* cells, which were then grown in LB medium containing 100 mg/L carbenicillin at 37 °C overnight. The pre-culture was diluted with LB medium containing 100 mg/L carbenicillin to an OD_600_ value of 0.1. Cells were grown at 37 °C until they reached an OD_600_ of approximately 0.6. Protein expression was induced by addition of 1 mM IPTG at 25 °C for 5 h. Then, cells were pelleted, resuspended in lysis buffer (50 mm Tris∙HCl (pH 8.0), 150 mM NaCl, 1 mM DTT, 1% Triton™ X-100, 0.1 M Pefabloc, 1 µg/mL aprotinin/leupeptin) and lysed by sonication at an amplitude of 20% for 30 s, three times. The lysate was clarified by high speed centrifugation and purified by immobilized metal ion affinity chromatography (IMAC) using a HisTrap^TM^-FF column (1 mL, GE Healthcare) on an ÄKTA system. The protein was eluted in 50 mM Tris∙HCl (pH 8.0) and 150 mM NaCl, with linear increasing concentrations of imidazole (30–500 mm). Fractions containing pure PGK1 protein were identified by 12.5% SDS-PAGE followed by Coomassie blue staining and pooled. The elution buffer was replaced by PGK1 storage buffer (50 mm Tris∙HCl (pH 8.0), 150 mM NaCl, 1 mM DTT, 10% glycerol) via dialysis overnight. The protein concentration was determined by BCA assay (Kit, Thermo Fisher Scientific). The protein was stored at −80 °C until use.

#### Determination of binding affinities by fluorescence polarization

Prior to the experiments the protein storage buffers were replaced by the respective fluorescence polarization assay buffers for LDHA (20 mM HEPES (pH 7.5), 200 mM NaCl, 0.01% Triton™ X-100) and PGK1 (20 mM Tris∙HCl (pH 7.5), 1 mM DTT, 5 mM MgCl_2_, 0.01% Triton™ X-100. The protein concentrations were determined by BCA assays and protein dilution series using the same buffers were prepared. *C*2-(5-FAM)-Ap_3_A (F-Ap_3_A) was added to the protein samples, yielding reaction mixtures containing 25 nM F-Ap_3_A and varying protein concentrations (as noted) in an overall volume of 110 µL. For each reaction mixture 30 µL were transferred to black 384-well non-binding microplates no. 781900 (Greiner Bio-One) in three separate wells. Samples were centrifuged (200×*g*, 2 min, 4 °C) and incubated on ice for 15 min followed by 30 min incubation at 30 °C. FP measurements were subsequently recorded on a Tecan infinite 500 plate reader. Emission was measured at 535 nm with two filters transmissive for polarized light, either parallel or perpendicular to the plane of excitation. The FP values were directly provided as millipolarization (mP). To correct for instrument intrinsic differences of the sensitivity towards detecting parallelly and perpendicularly emitted polarized light, the G-factor was determined and used for subsequent measurements. For this calibration, the FP value of a 1 nM 5-FAM solution in 0.01 M NaOH was measured and set to 20 mP. The mean and the standard deviation were calculated for each sample. The binding curves were corrected by subtracting the background FP value. Data was analyzed with GraphPad PRISM (version 6.01) and curve fittings were calculated by using the equation for one site specific binding (*K*_D_) with the maximum specific binding *B*_max_ and the equilibrium constant *K*_D_. The obtained binding parameters are shown in Supplementary Table 1.

### Statistic and reproducibility

The stability of Ap_3_A and Ap_4_A in human cell lysates was monitored twice with comparable results. Determination of Ap_3_A and Ap_4_A levels were performed in 15 biological replicates for each condition. Samples were measured in technical duplicates and averaged. Measurements with a signal to noise ratio below 7 were excluded. All PAL experiments were performed in biological triplicates. Western blot analysis of isolated proteins was performed for each replicate with comparable results. After digestion of the proteins, nano-LC-MS/MS was measured for each replicate in technical duplicates. The cleavage reaction of m^7^Gp_3_G by DcpS was performed in biological triplicates. The transthioesterification was performed twice as biological replicates with matching results. All FP measurements were performed in technical triplicates. For details of statistical analysis see respective methods section and figure legends.

## Supplementary information

Supplementary Information

Description of Additional Supplementary Files

Supplementary Dataset 1

Supplementary Dataset 2

Supplementary Dataset 3

Supplementary Dataset 4

## Data Availability

The data that support this study are available from the corresponding authors upon reasonable request. The mass spectrometry proteomics data generated in this study have been deposited in the ProteomeXchange Consortium via the PRIDE partner repository^55^ under accession code PXD020740. Figures with associated raw data: Figs. 2–7 and Supplementary Figs. 1, 6, 7, 9 and 10. Source data are provided with this paper. For protein identification, the human and the *E. coli* reference proteome downloaded from the UniProt database (https://www.uniprot.org/proteomes/) (download date: 02.04.2019 and 01.10.2019) and an integrated database of common contaminants were used. For GO term analysis Human and *E. coli* gene association (.gaf) and ontology files (.obo) were downloaded from geneontology.org (http://geneontology.org) (27.01.2020). Source data are provided with this paper.

## Source data

Source Data
