# Supplementary material for: Chemical proteomic profiling reveals protein interactors of the alarmones diadenosine triphosphate and tetraphosphate
Source: Nat Commun. 2021 Oct 4;12:5808. doi: 10.1038/s41467-021-26075-4 (PMC8490401; doi:10.1038/s41467-021-26075-4)
Supplement: Supplementary file 1 — Supplementary Information [file 41467_2021_26075_MOESM1_ESM.pdf]

Chemical proteomic profiling of the alarmones diadenosine triphosphate and tetraphosphate reveals protein interactors

Lena Krüger[1,2,+], Christoph J. Albrecht[1,2,+], Hannah K. Schammann[1], Florian M. Stumpf[1,2], Marie L. Niedermeier[2,3], Yizhi Yuan[1,2], Katrin Stuber[1,2,3], Josua Wimmer[1], Florian Stengel[2,3], Martin Scheffner[2,3], Andreas Marx[1,2*]

[1]Department of Chemistry, University of Konstanz, Konstanz, Germany

[2]Konstanz Research School-Chemical Biology, University of Konstanz, Konstanz, Germany

[3]Department of Biology, University of Konstanz, Konstanz, Germany

*Correspondence: A.M. (andreas.marx@uni-konstanz.de)

[+]These authors contributed equally to this work.

Supplementary Information

Supplementary figures

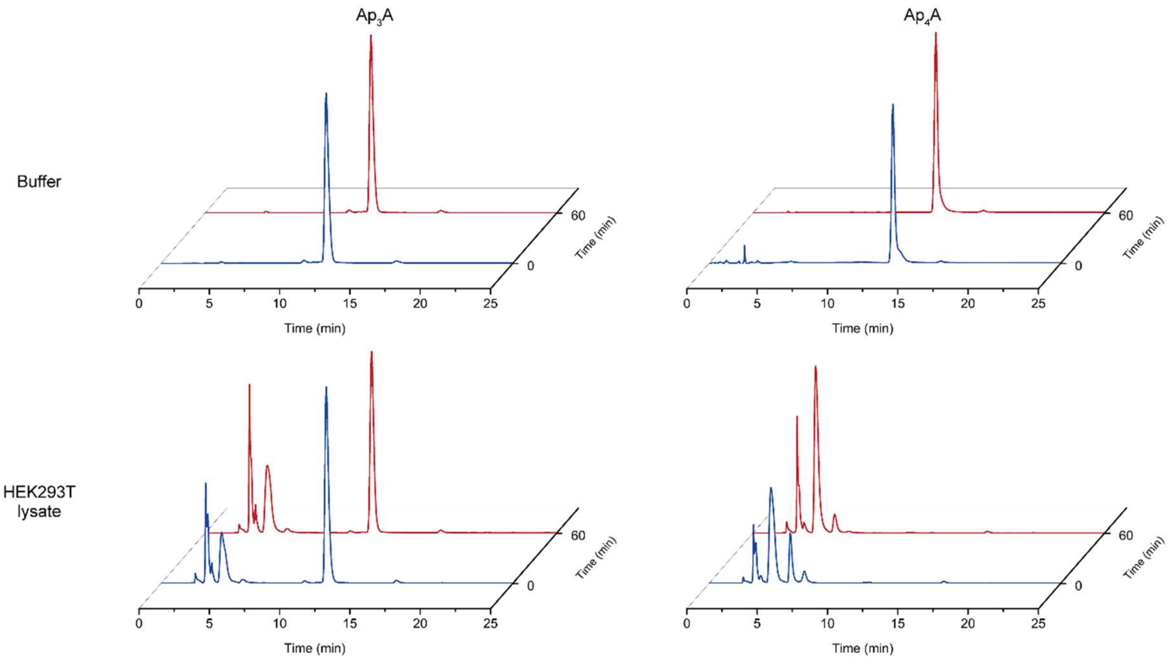

Supplementary Fig. 1: HPLC monitoring of the stability of natural Ap_3A and Ap_4A.
200 µM Ap_nAs were incubated in 20 mM HEPES (pH 7.4), 100 mM NaCl, 5 mM $MgCl_2$ and 1 mM DTT in the presence or absence of 2 mg/mL HEK293T cell lysates at 37 °C for 1 h. Samples incubated for 0 and 60 min were taken and analyzed via RP-HPLC. Comparable experiments were performed using different human cell lysates with similar results.

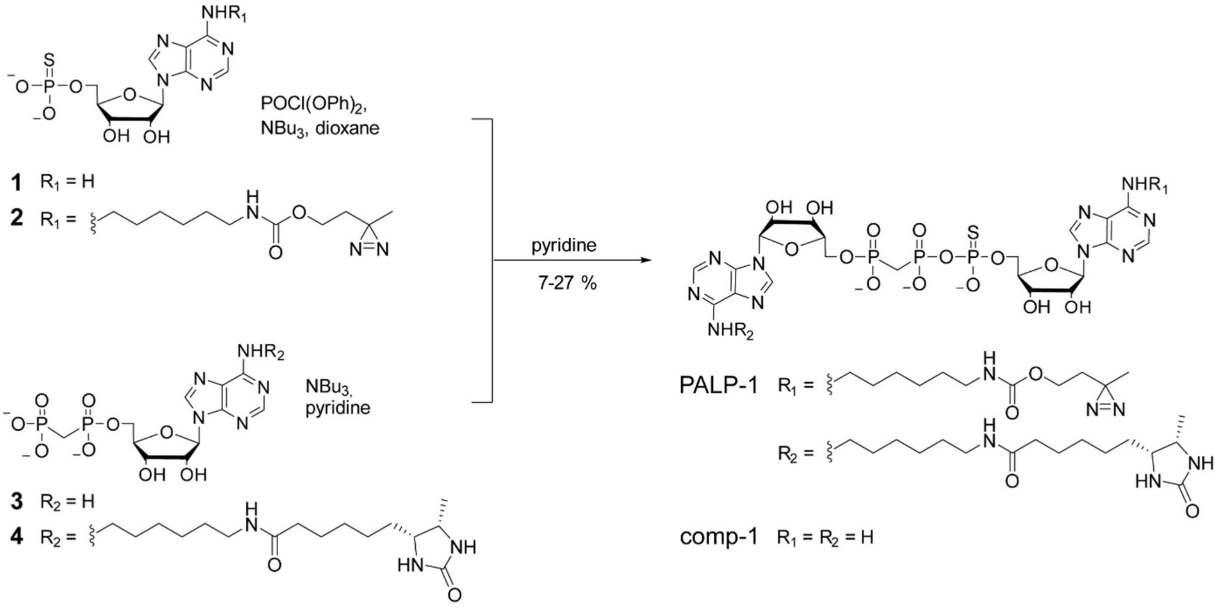

Supplementary Fig. 2: General approach for the synthesis of non-hydrolysable Ap_3A analogs.
Thiomonophosphate derivatives (**1** and **2**) are activated with $POCl(OPh)_2$. Subsequent coupling to the bisphosphonates (**3** and **4**) results in the formation of Ap_3A derivatives (PALP-1 and comp-1).[1]

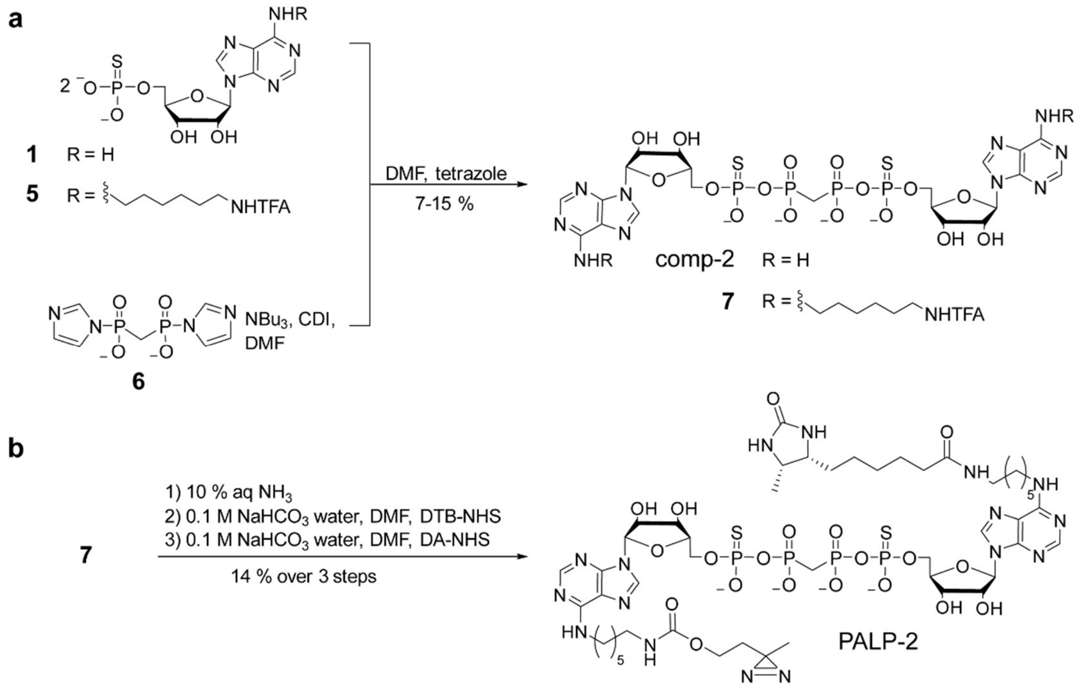

Supplementary Fig. 3: General approach for the synthesis of non-hydrolysable Ap$_4$A analogs.
a Bisphosphonic acid is activated with CDI to form the bis-imidazolide (**6**).[2] Tetrazole catalyzed reaction with the monophosphates results in the formation of symmetric Ap$_4$A analogs (**7** and comp-2). **b** Subsequent deprotection under basic conditions and consecutive NHS ester coupling of DTB and DA gives PALP-2.[3]

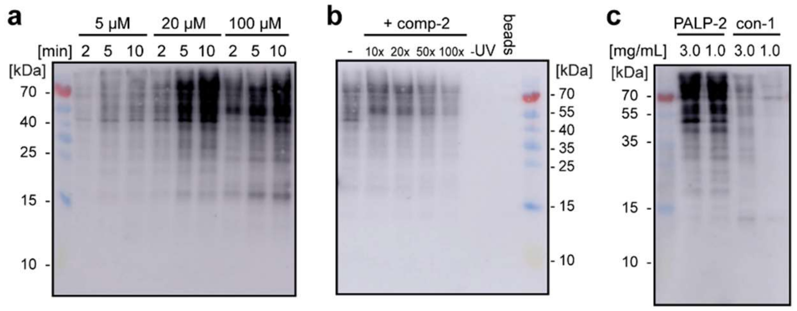

Supplementary Fig. 4: Optimization of PAL experiments with PALP-2 analyzed by western blot using ExtrAvidin®-Peroxidase (Sigma-Aldrich).
a PALP-2 concentration and irradiation time dependent photoaffinity-labeling efficiency. **b** Effect of preincubation with 10 to 100-times excess of comp-2 to 20 µM PALP-2. UV-control was performed without irradiation and beads control without probe. **c** Comparison between affinity enriched proteins for PALP-2 (20 µM) and con-1 (20 µM), depending on lysate concentration (1.0 and 3.0 mg/mL). Source data are provided as a Source Data file. Comparable experiments were performed using PALP-3 with similar results.

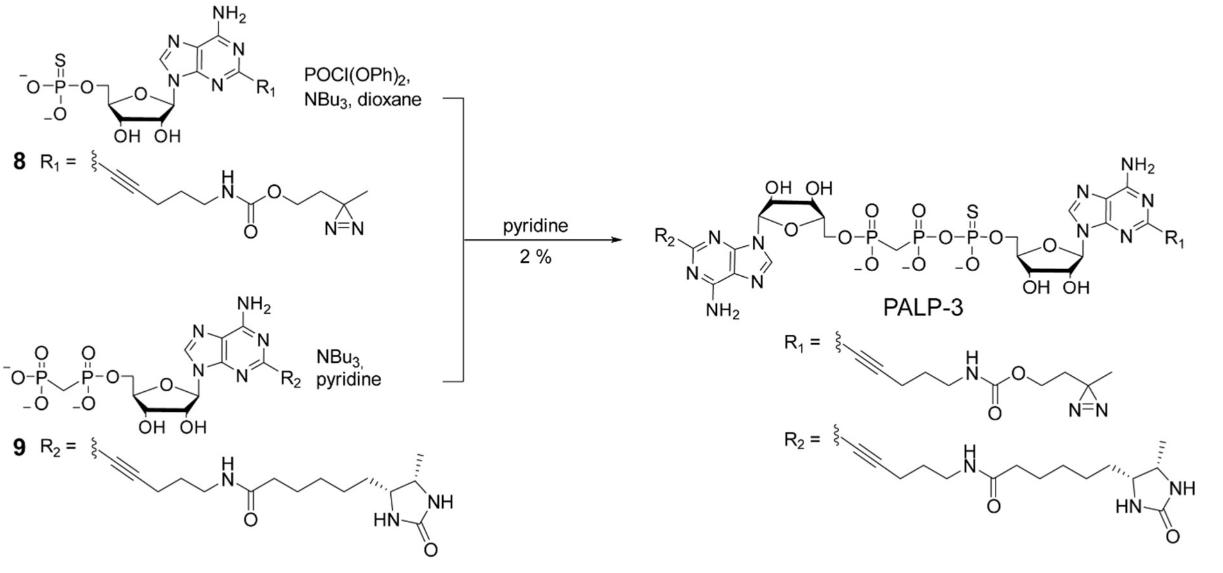

Supplementary Fig. 5: Synthesis of C2-modified non-hydrolysable Ap$_3$A analog (PALP-3).
Thiomonophosphate derivative **8** is activated with POCl(OPh)$_2$. Subsequent coupling to the bisphosphonate **9** results in the formation of PALP-3.[1]

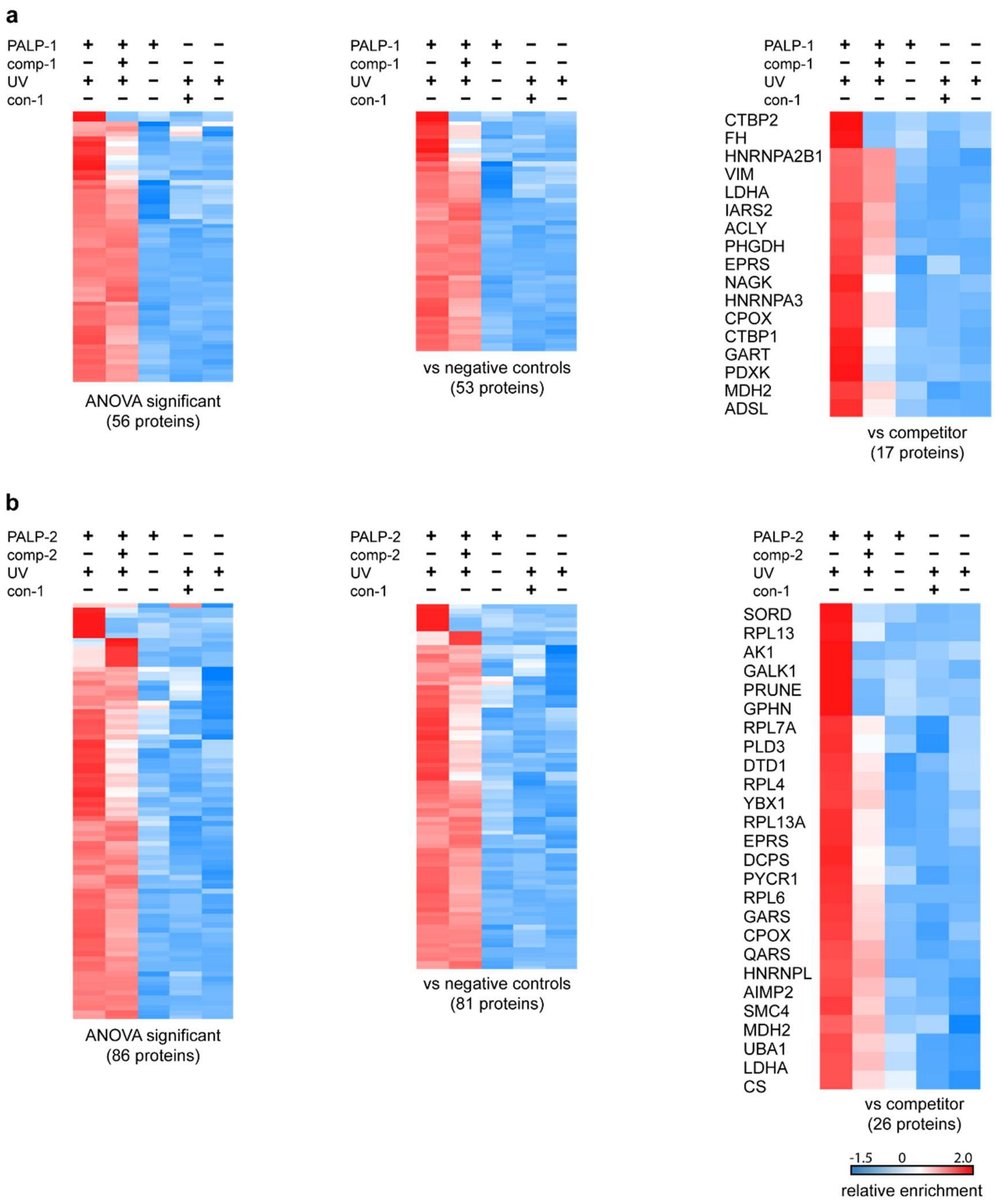

Supplementary Fig. 6: Heat map representation (Z-scores) of proteins significantly enriched in PAL experiments with HEK293T lysates using PALP-1 or PALP-2 after the consecutively performed statistical filtration steps (n = 3).
Proteins identified applying PALP-1 **a** and PALP-2 **b** that were deemed significant by one-way ANOVA after multiple-sample test (S_0 = 0.2, FDR ≤ 0.01) (left); significant pairs with negative controls after two-sided Post hoc Tukey's HSD test (FDR ≤ 0.05) (middle); significant pairs with competitor control after two-sided Post hoc Tukey's HSD test (FDR ≤ 0.05) (right).

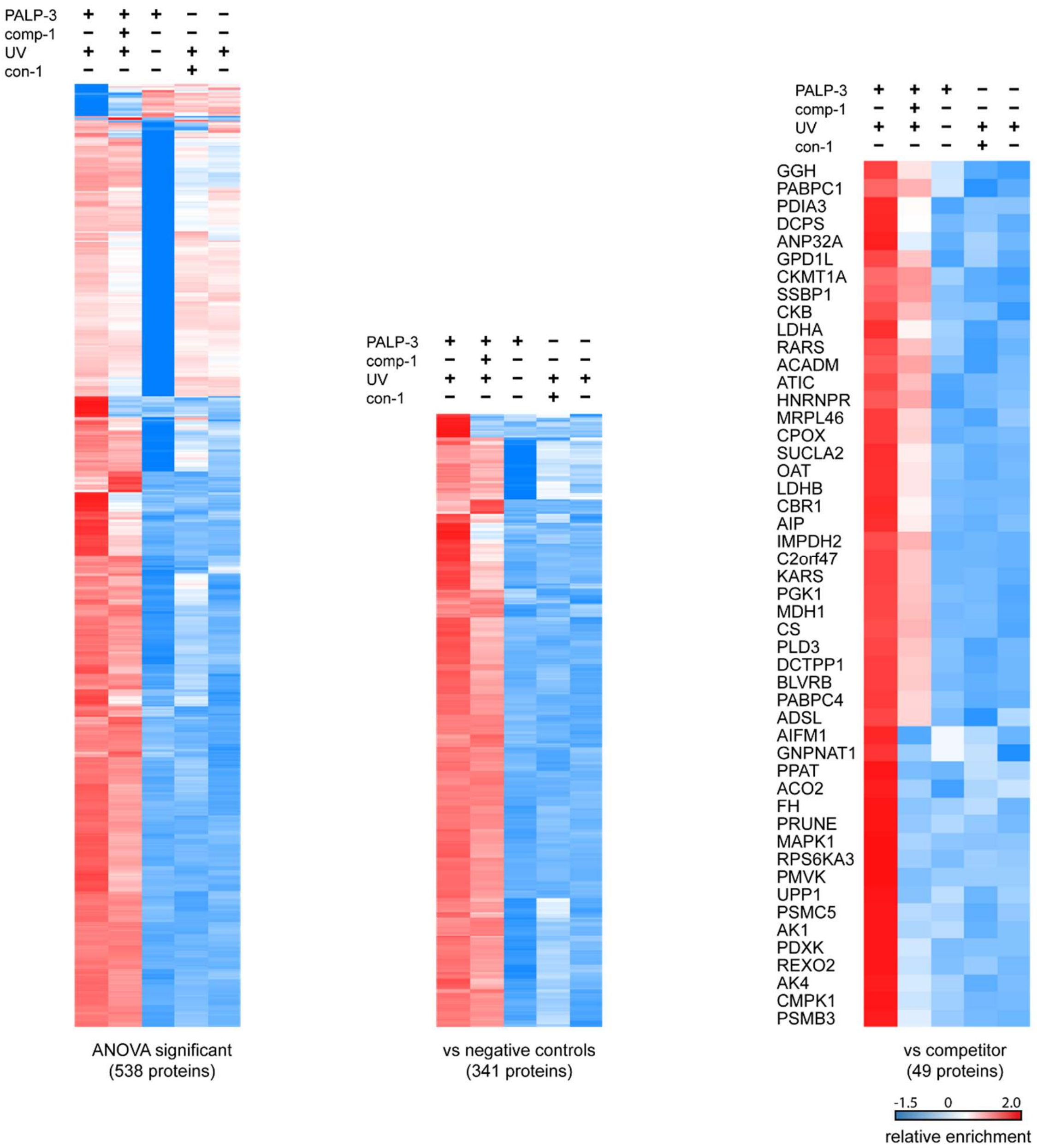

Supplementary Fig. 7: Heat map representation (Z-scores) of proteins significantly enriched in PAL experiments with HEK293T lysates using PALP-3 after the consecutively performed statistical filtration steps (n = 3).

Proteins identified applying PALP-3 that were deemed significant by one-way ANOVA after multiple-sample test (S_0 = 0.2, FDR ≤ 0.01) (left); significant pairs with negative controls after two-sided Post hoc Tukey's HSD test (FDR ≤ 0.05) (middle); significant pairs with competitor control after two-sided Post hoc Tukey's HSD test (FDR ≤ 0.05) (right).

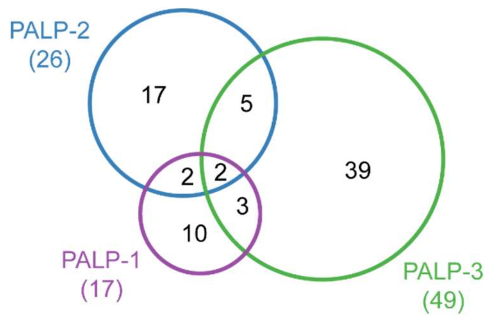

Supplementary Fig. 8: Venn diagram showing the distribution of identified proteins for PALP-1 (purple), PALP-2 (blue) and PALP-3 (green).

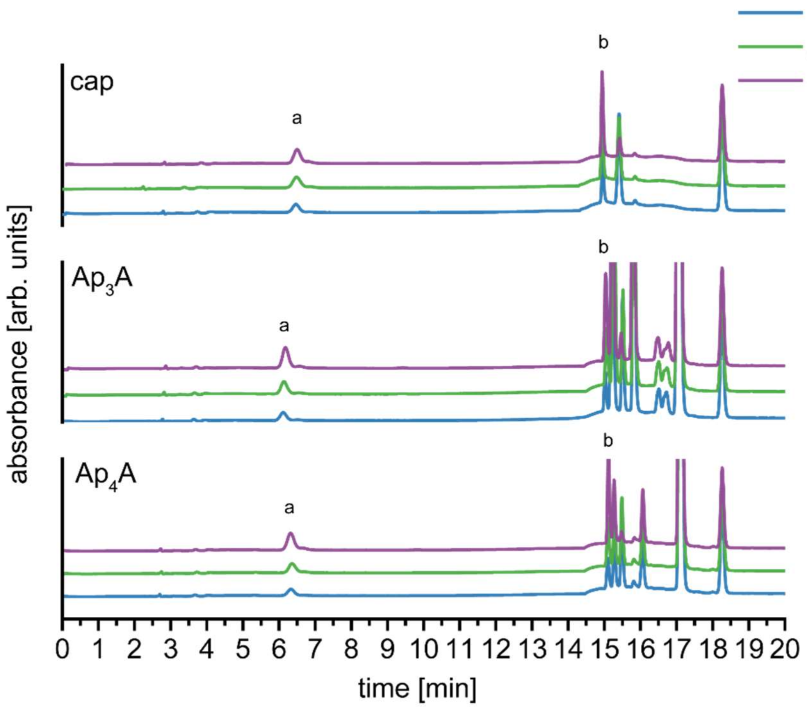

Supplementary Fig. 9: HPLC profiles for the hydrolysis of m^{7}GpppG catalyzed by DcpS.
The initial substrate concentration was 20 µM and the reactions were carried out in the absence or presence of Ap_3A and Ap_4A (200 µM) at an enzyme concentration of 100 nM. The reactions were stopped after 1 (blue), 2 (green) or 5 minutes (blue) and loaded to an analytical HPLC. Absorbance was measured at 260 nm (arb. units, arbitrary units). Formation of m^{7}GMP (peak a) and GDP (peak b) was observed for all reactions. The chromatographic peaks were identified by comparison with the retention times of reference samples and by subsequent MS analysis. The experiments were performed three times as biological replicates with matching results.

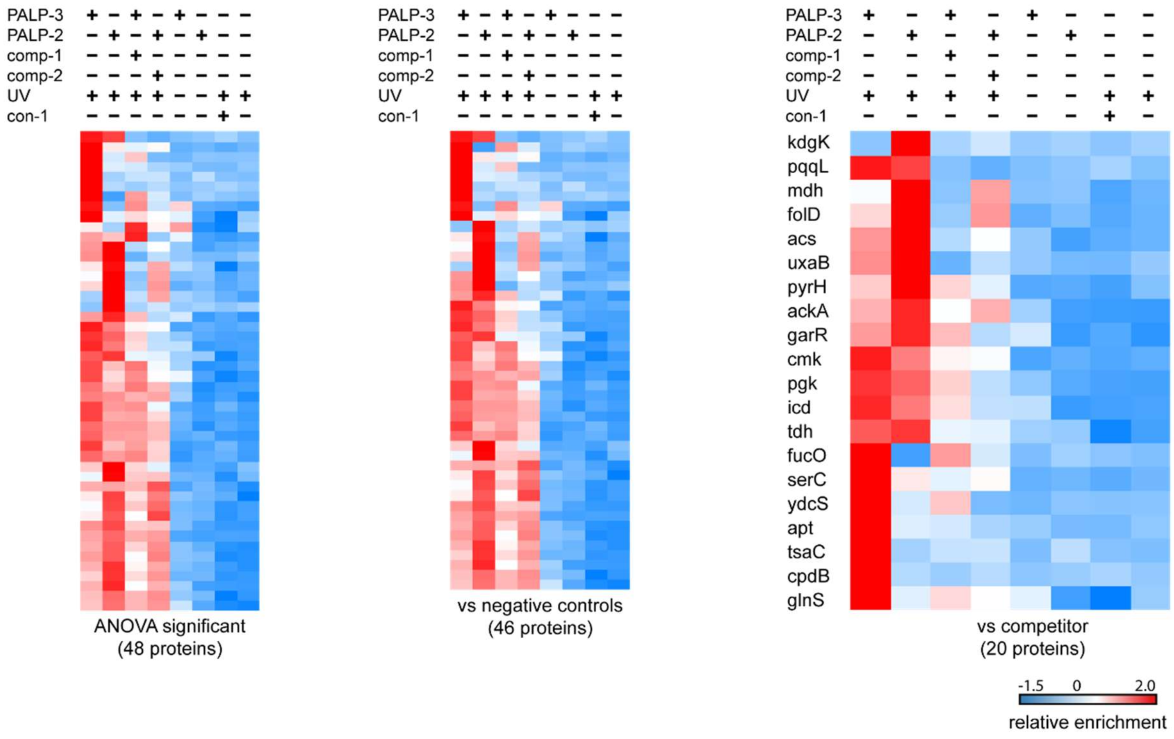

Supplementary Fig. 10: Heat map representation (Z-scores) of proteins significantly enriched in PAL experiments with *E. coli* K12 lysates after the consecutively performed statistical filtration steps (n = 3). Proteins identified applying PALP-2 and PALP-3 that were deemed significant by one-way ANOVA after multiple-sample test (S_0 = 0.2, FDR ≤ 0.01) (left); significant pairs with negative controls after two-sided Post hoc Tukey's HSD test (FDR ≤ 0.05) (middle); significant pairs with competitor control after two-sided Post hoc Tukey's HSD test (FDR ≤ 0.05) (right).

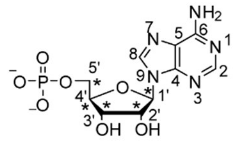

^{1}**H NMR** (600 MHz, D_2O) of **10**

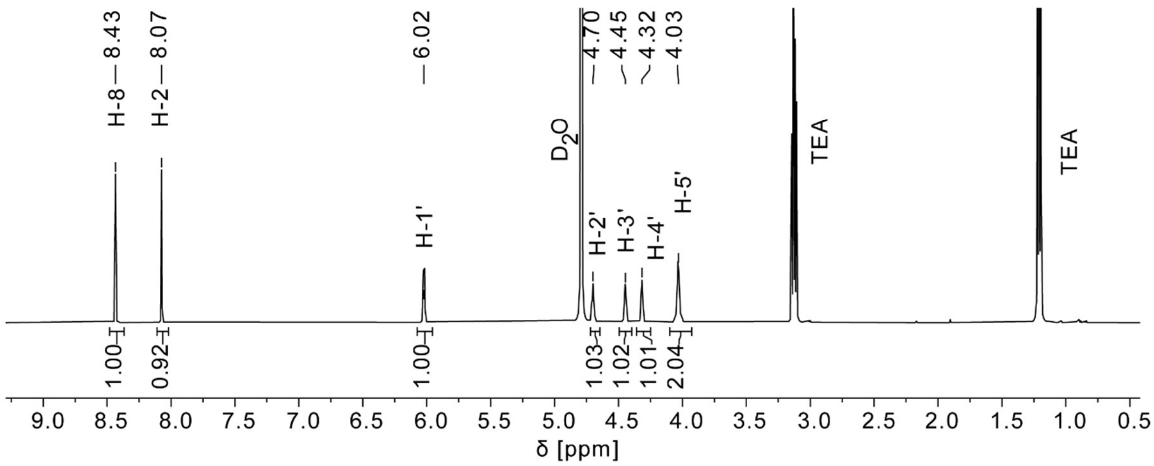

^{13}**C NMR** (151 MHz, D_2O) of **10**

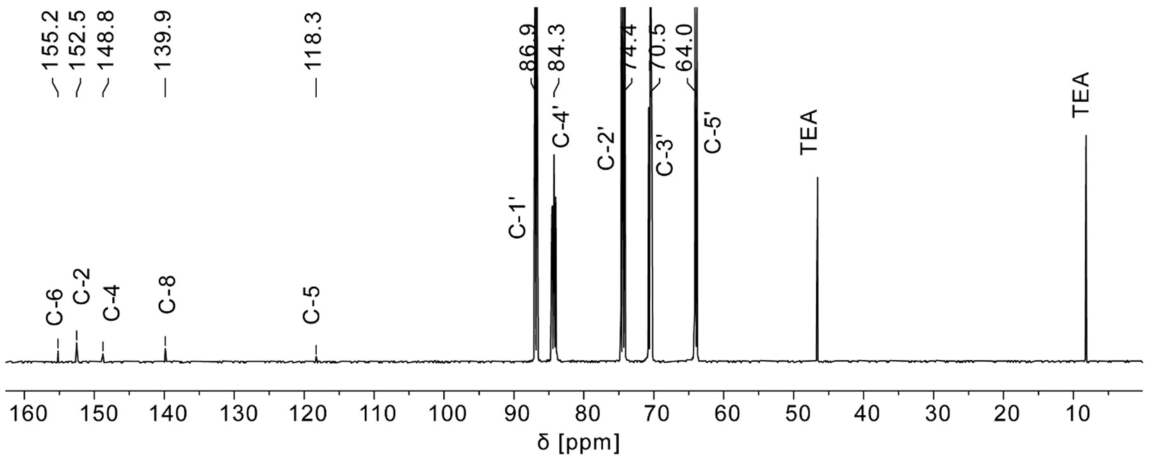

^{31}**P NMR** (162 MHz, D_2O) of **10**

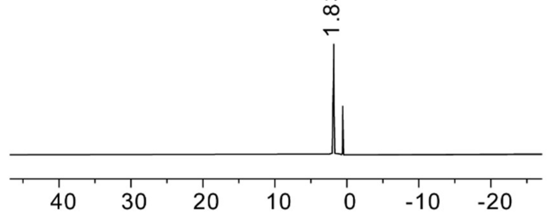

Supplementary Fig. 11: Structural characterization of $^{13}C_5$-adenosine-5'-monophosphate (10) by nuclear magnetic resonance spectroscopy (NMR).

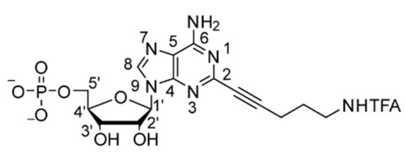

^{1}H NMR (400 MHz, D₂O) of **11**

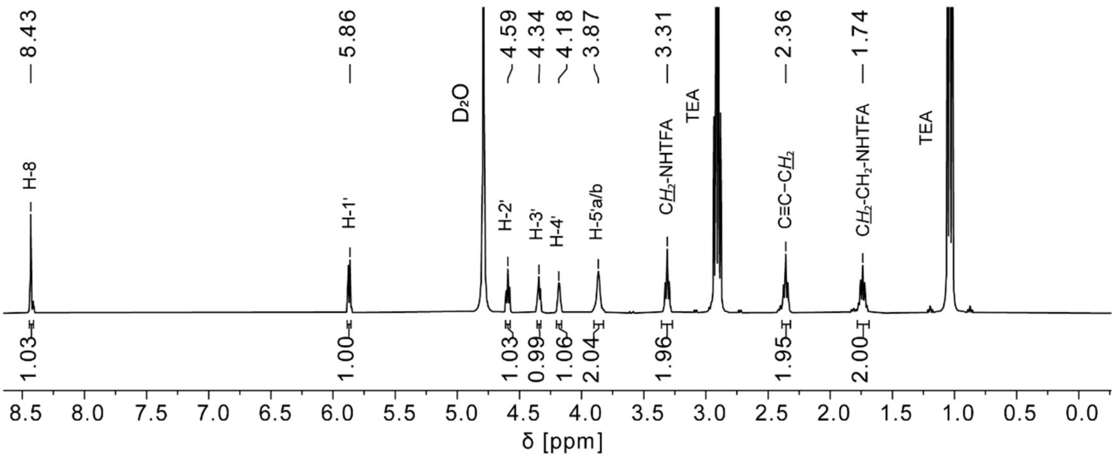

^{13}C NMR (126 MHz, D₂O) of **11**

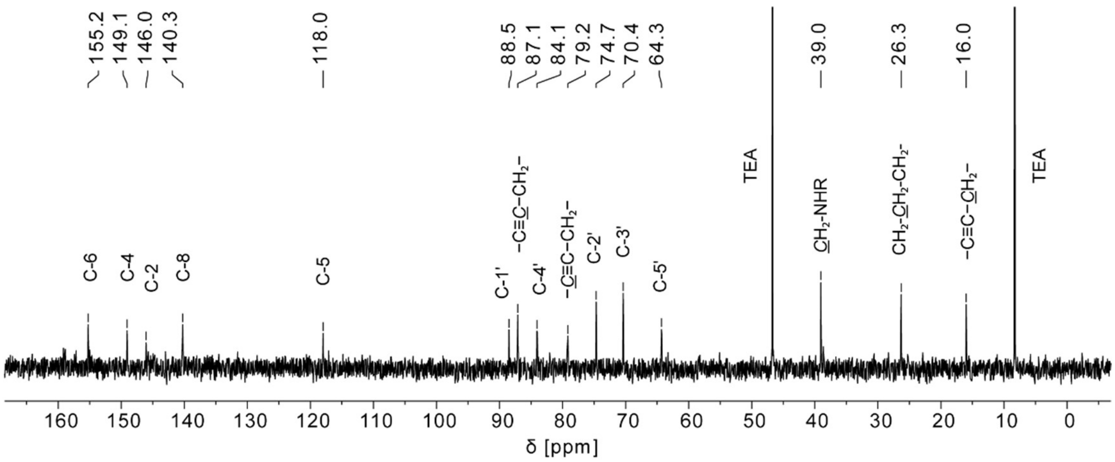

^{31}P NMR (162 MHz, D₂O) of **11**

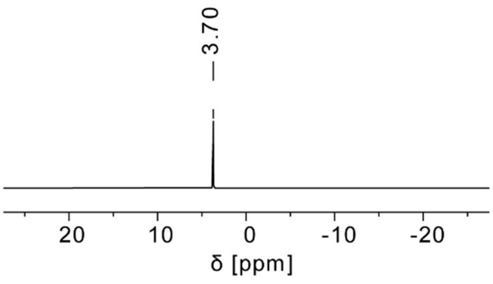

^{19}F NMR (376 MHz, D₂O) of **11**

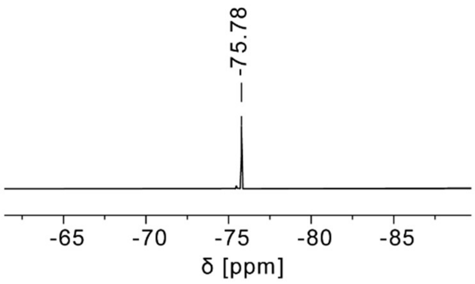

Supplementary Fig. 12: Structural characterization of *C*2-(5-trifluoroacetamidopent-1-yn-1-yl)-adenosine 5'-monophosphate (11) by nuclear magnetic resonance spectroscopy (NMR).

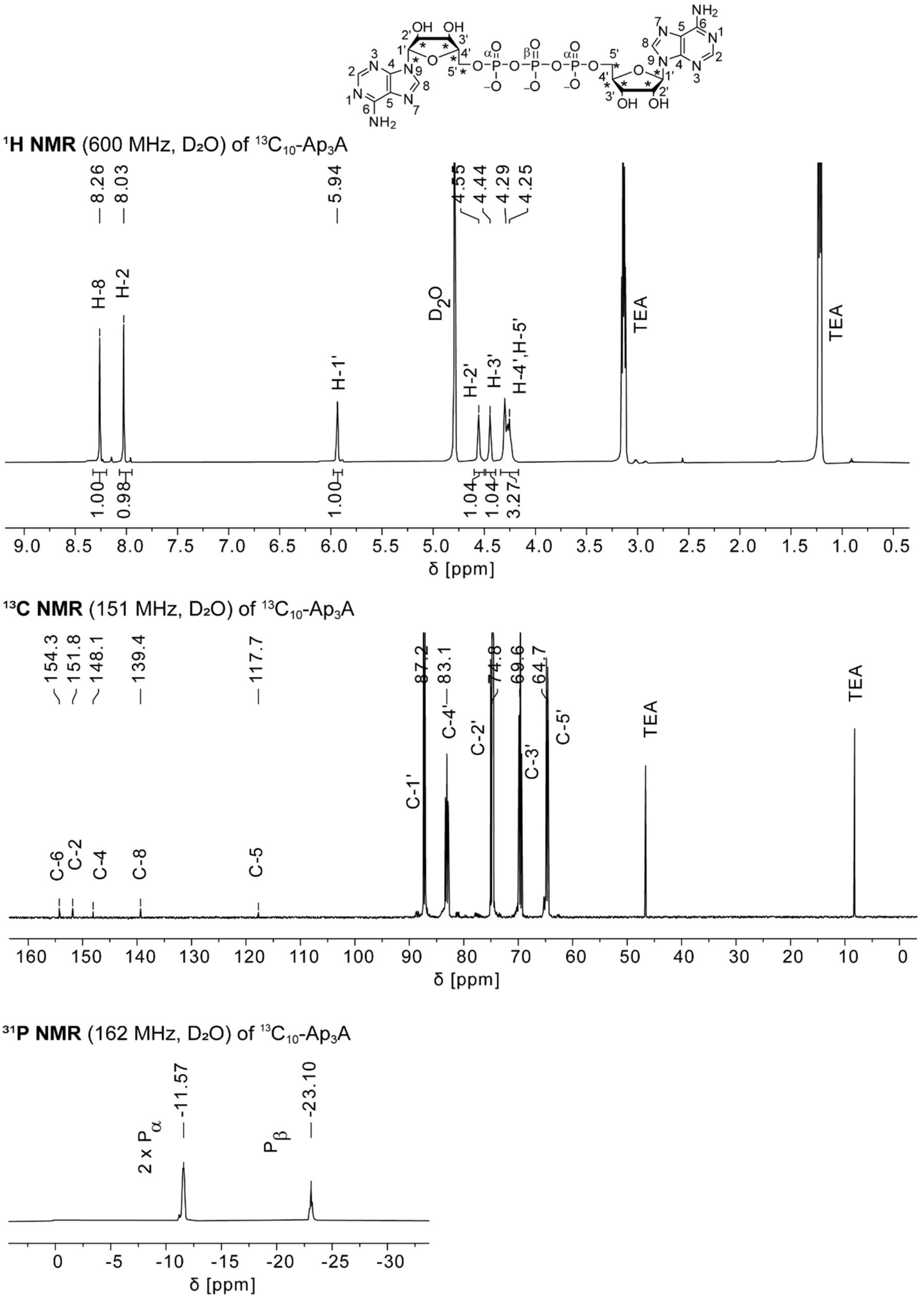

Supplementary Fig. 13: Structural characterization of $^{13}C_{10}$-*P*[1],*P*[3]-diadenosine-5'-triphosphate ($^{13}C_{10}$-Ap$_3$A) by nuclear magnetic resonance spectroscopy (NMR).

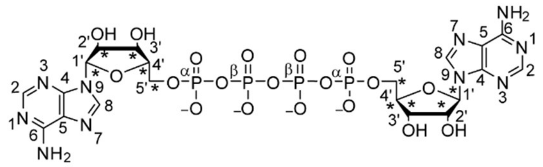

^{1}**H NMR** (600 MHz, D_2O) of $^{13}C_{10}$-Ap_4A

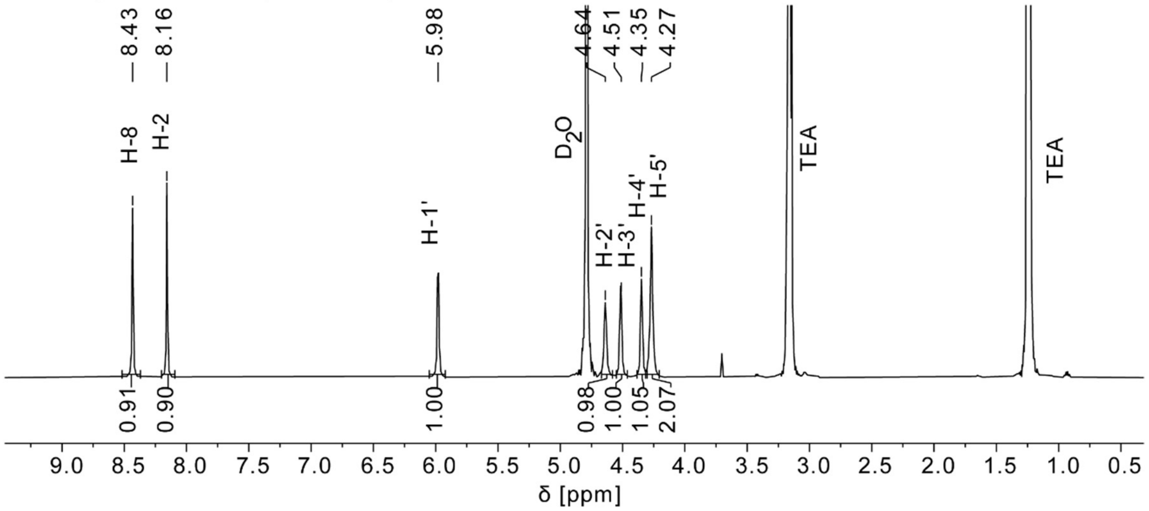

^{13}**C NMR** (151 MHz, D_2O) of $^{13}C_{10}$-Ap_4A

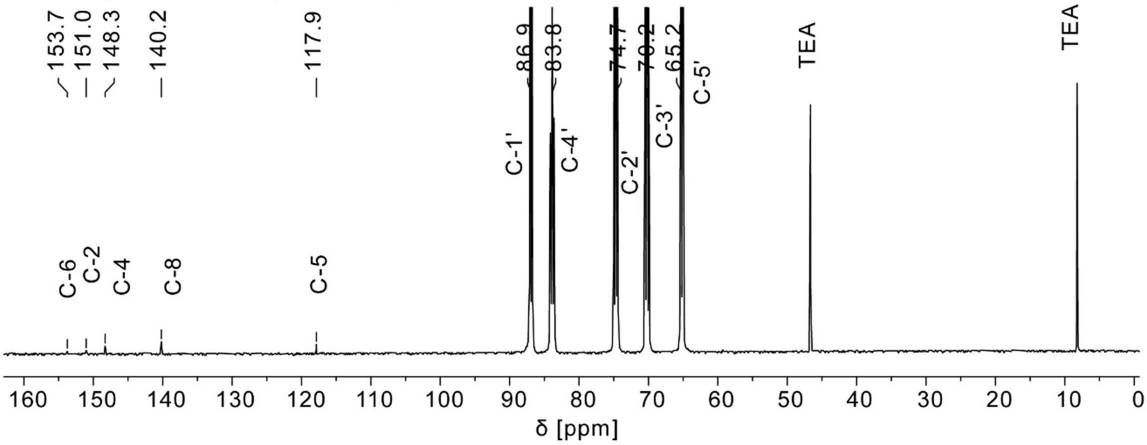

^{31}**P NMR** (162 MHz, D_2O) of $^{13}C_{10}$-Ap_4A

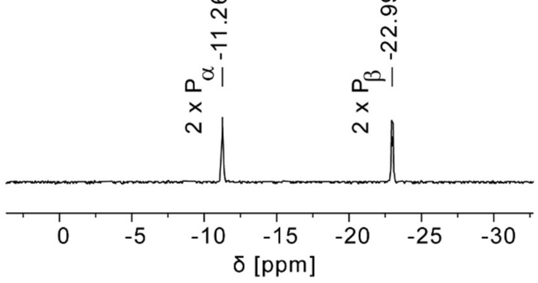

Supplementary Fig. 14: Structural characterization of $^{13}C_{10}$-*P*^{1},*P*^{4}-diadenosine-5'-tetraphosphate ($^{13}C_{10}$-Ap_4A) by nuclear magnetic resonance spectroscopy (NMR).

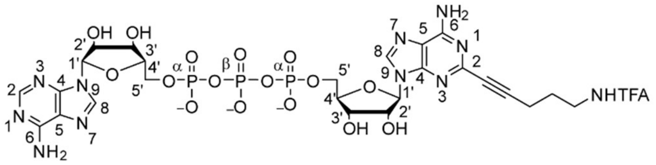

^{1}H NMR (400 MHz, D_2O) of **12**

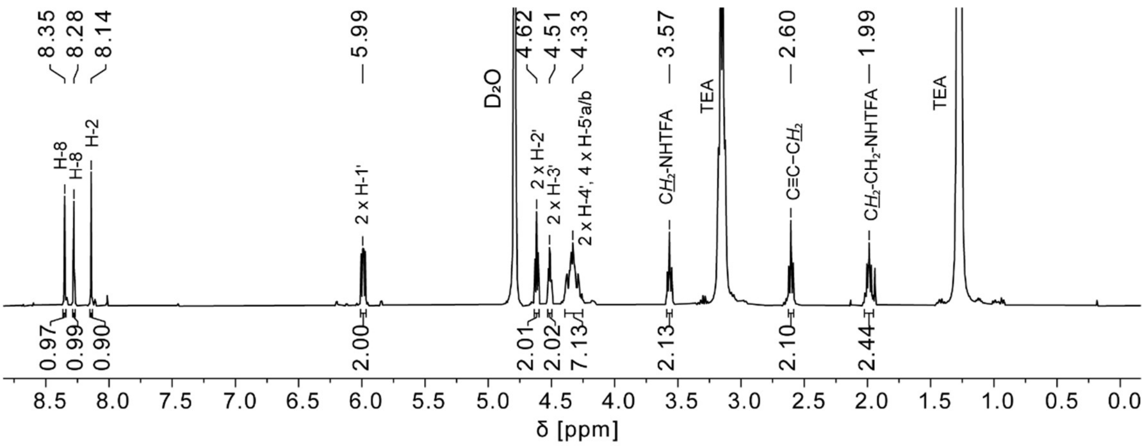

^{31}P NMR (162 MHz, D_2O) of **12**

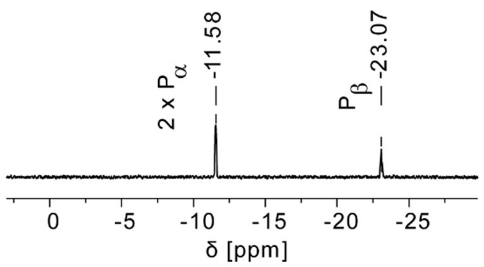

^{19}F NMR (376 MHz, D_2O) of **12**

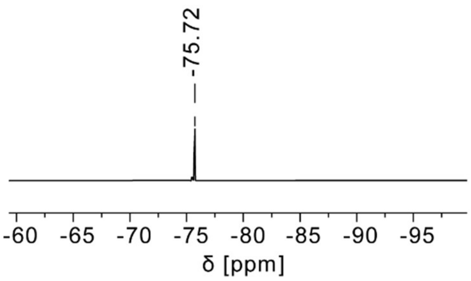

Supplementary Fig. 15: Structural characterization of *C*2-(5-trifluoroacetamido-pent-1-yn-1-yl)-adenosine-5'-(adenosine-5')-triphosphate (12) by nuclear magnetic resonance spectroscopy (NMR).

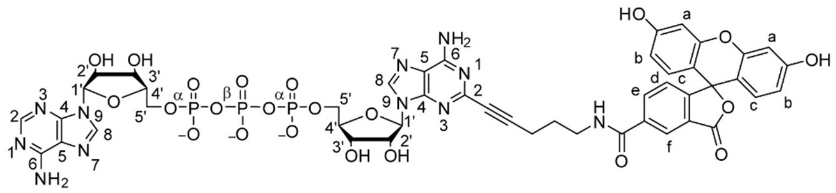

^{1}H NMR (500 MHz, D_2O) of F-Ap_3A

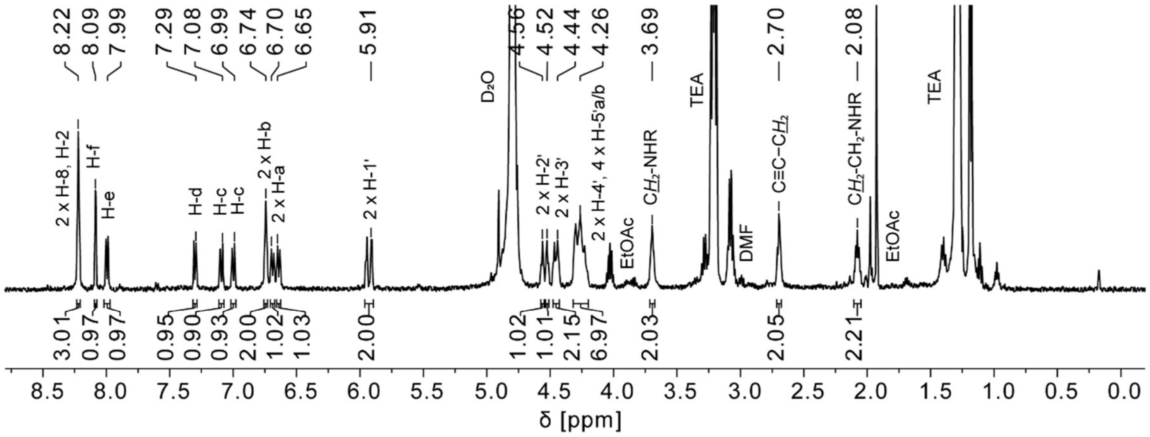

^{31}P NMR (162 MHz, D_2O) of F-Ap_3A

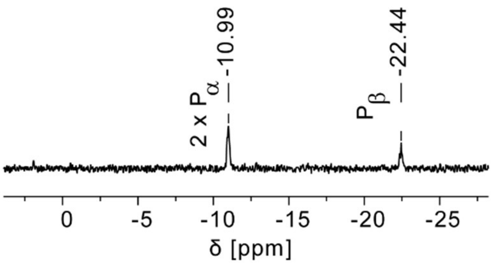

Supplementary Fig. 16: Structural characterization of *C*2-(5-FAM)-Ap_3A (F-Ap_3A) by nuclear magnetic resonance spectroscopy (NMR).

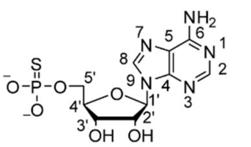

¹H NMR (400 MHz, D_2O) of **1**

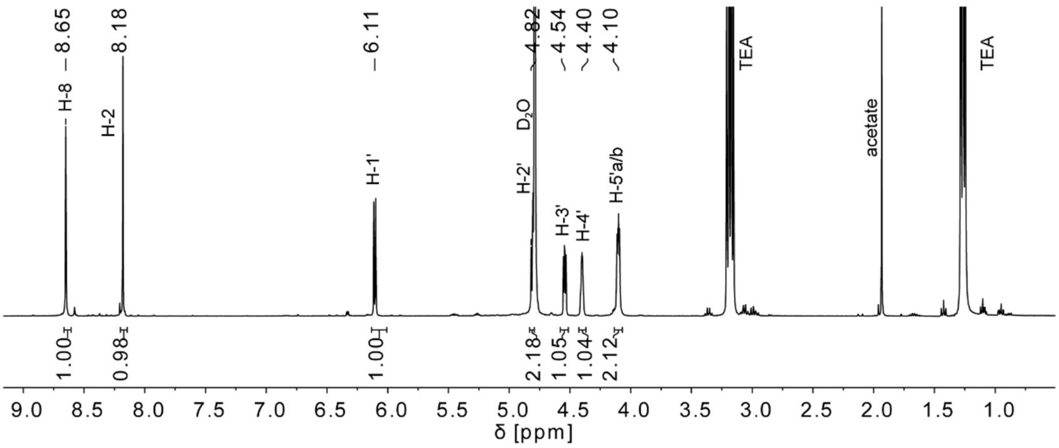

¹³C NMR (151 MHz, D_2O) of **1**

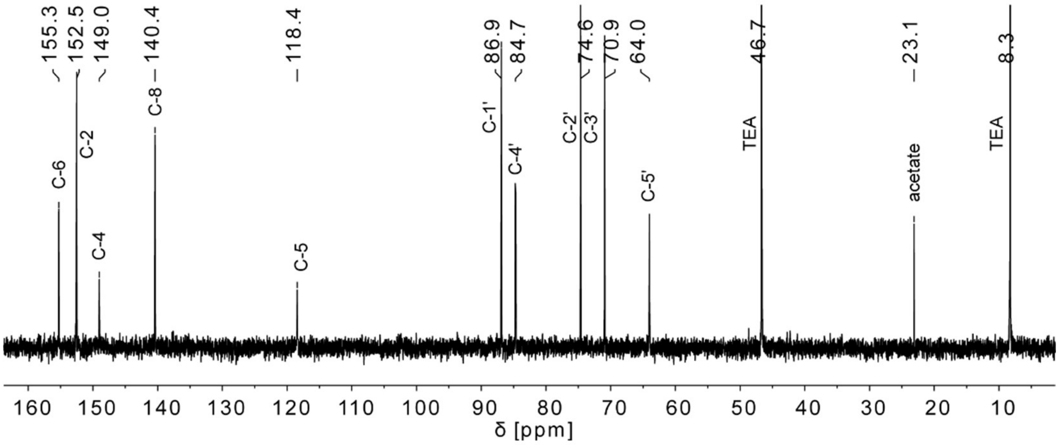

³¹P NMR (162 MHz, D_2O) of **1**

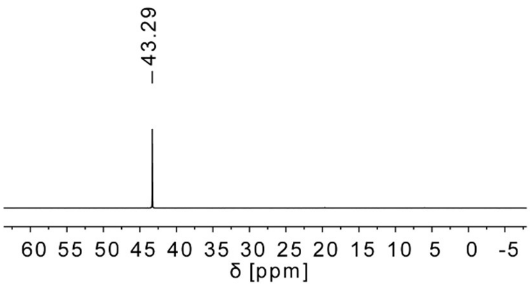

Supplementary Fig. 17: Structural characterization of adenosine 5'-thiomonophosphate (1) by nuclear magnetic resonance spectroscopy (NMR).

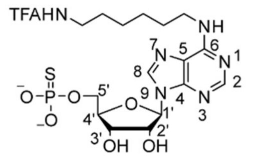

^{1}**H NMR** (400 MHz, D_2O) of **5**

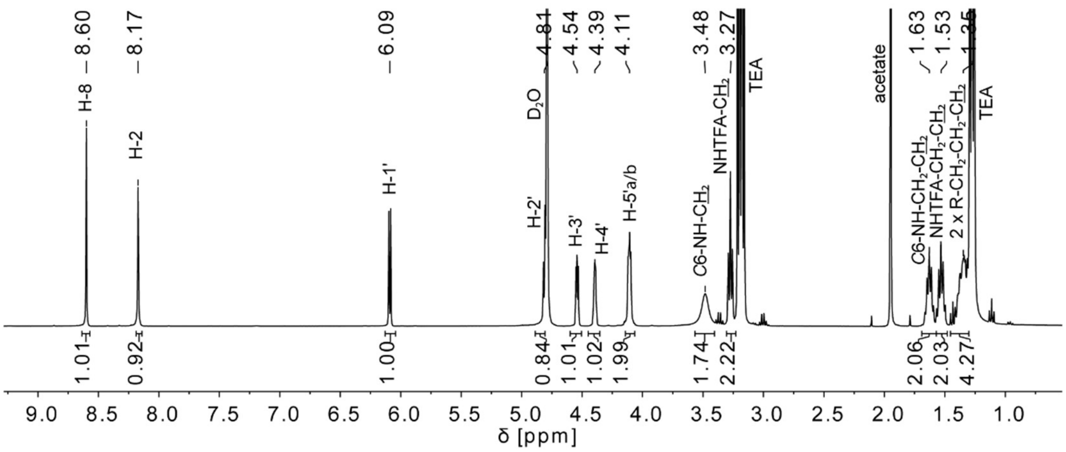

^{13}**C NMR** (151 MHz, D_2O) of **5**

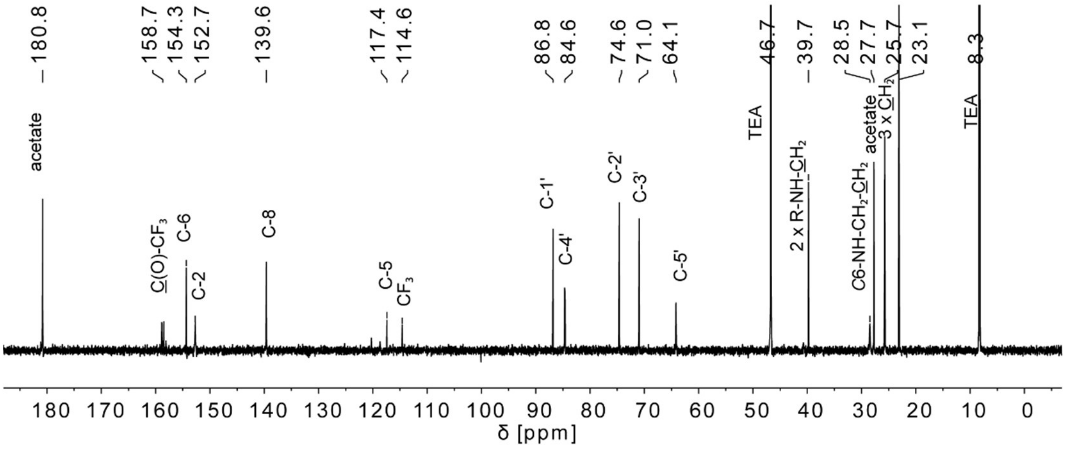

^{31}**P NMR** (162 MHz, D_2O) of **5**

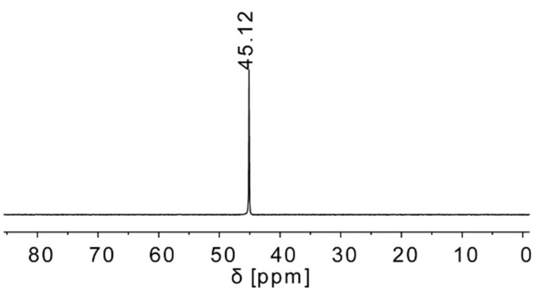

^{19}**F NMR** (376 MHz, D_2O) of **5**

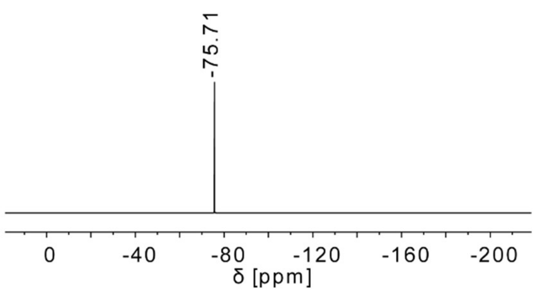

Supplementary Fig. 18: Structural characterization of *N*6-(6-trifluoroacetamidohexyl)-adenosine 5'-thiomonophosphate (5) by nuclear magnetic resonance spectroscopy (NMR).

Supplementary Fig. 19: Structural characterization of *C*2-(5-trifluoroacetamidopent-1-yn-1-yl)-adenosine 5'-thiomonophosphate (13) by nuclear magnetic resonance spectroscopy (NMR).

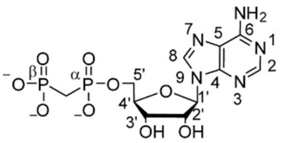

^{1}**H NMR** (400 MHz, D_2O) of **3**

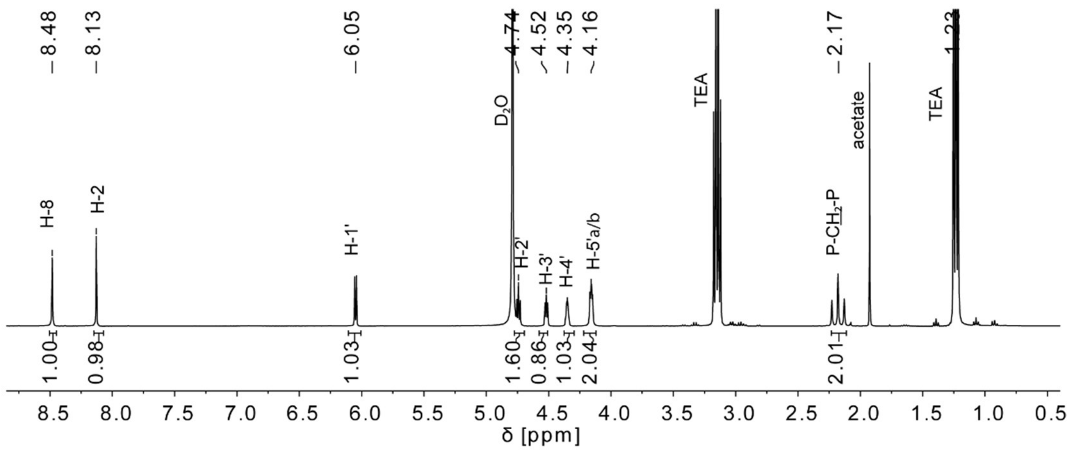

^{13}**C NMR** (151 MHz, D_2O) of **3**

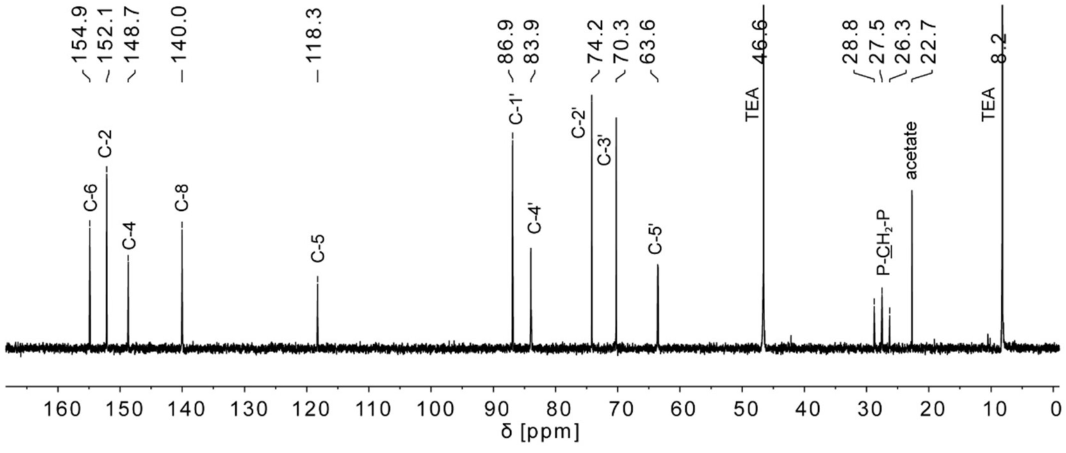

^{31}**P NMR** (162 MHz, D_2O) of **3**

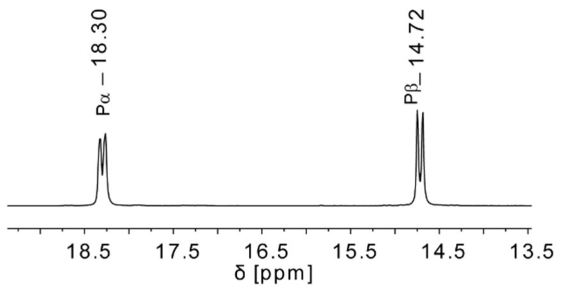

Supplementary Fig. 20: Structural characterization of adenosine 5'-methylene bisphosphonate (3) by nuclear magnetic resonance spectroscopy (NMR).

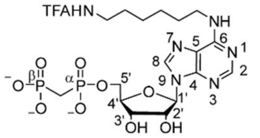

^{1}**H NMR** (400 MHz, D$_2$O) of **14**

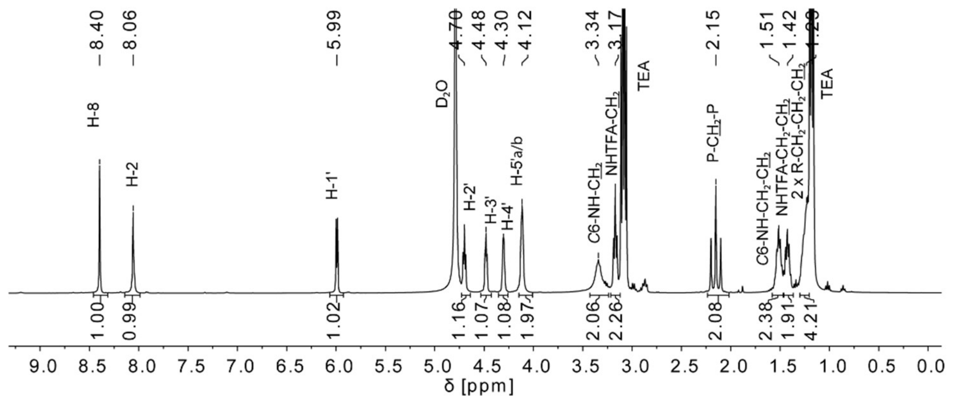

^{13}**C NMR** (151 MHz, D$_2$O) of **14**

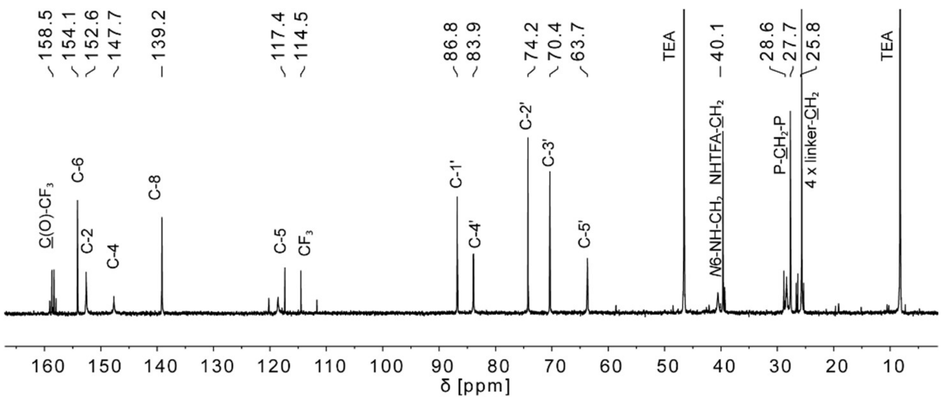

^{31}**P NMR** (162 MHz, D$_2$O) of **14**

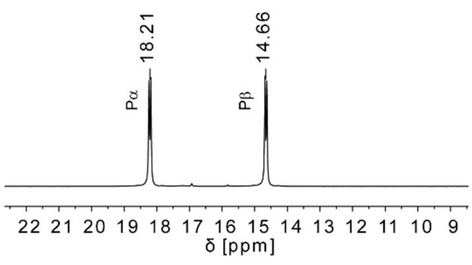

^{19}**F NMR** (376 MHz, D$_2$O) of **14**

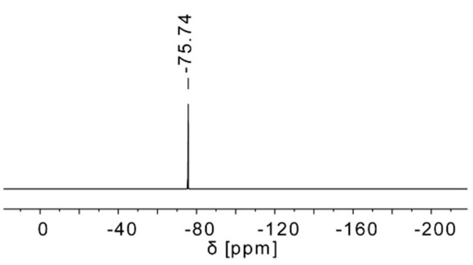

Supplementary Fig. 21: Structural characterization of *N*6-(6-trifluoroacetamidohexyl)-adenosine 5'-methylene bisphosphonate (14) by nuclear magnetic resonance spectroscopy (NMR).

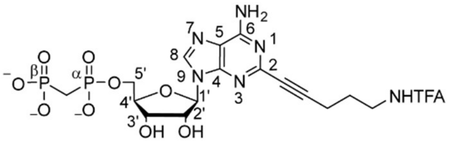

^{1}**H NMR** (400 MHz, D_2O) of **15**

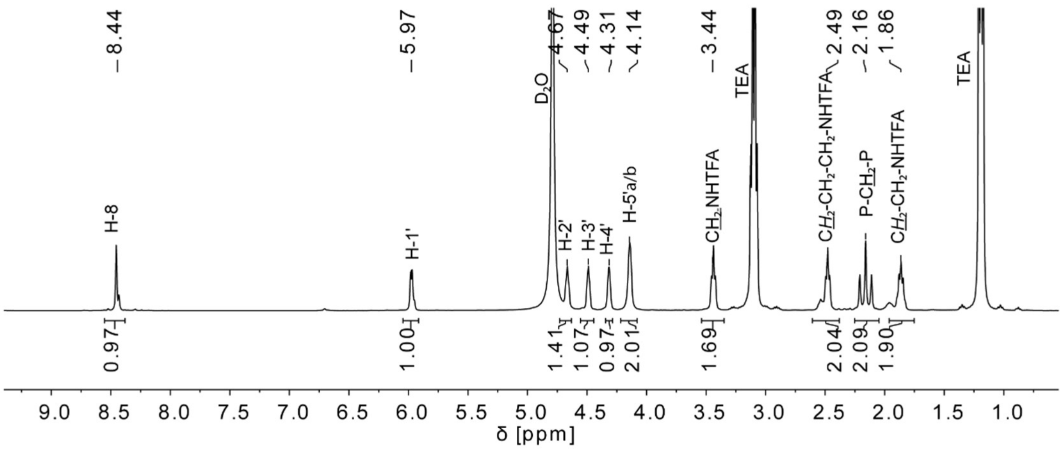

^{13}**C NMR** (151 MHz, D_2O) of **15**

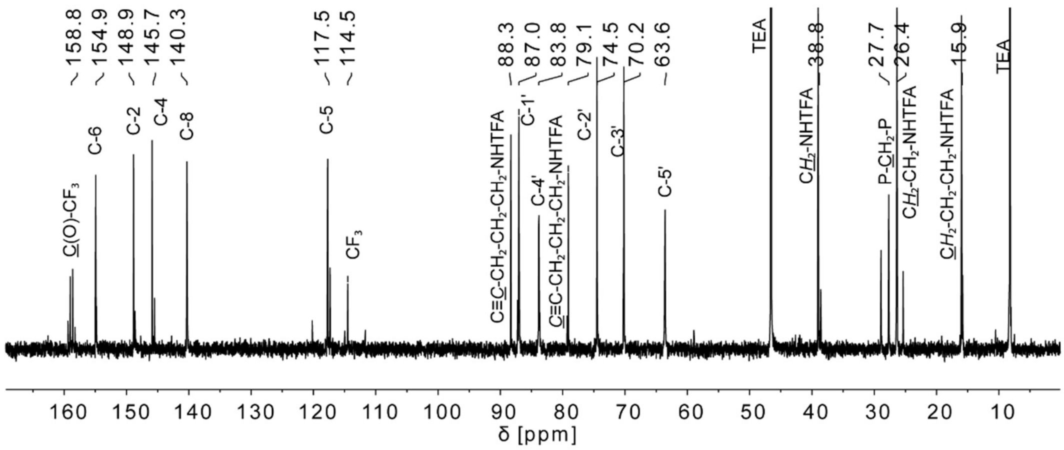

^{31}**P NMR** (162 MHz, D_2O) of **15**

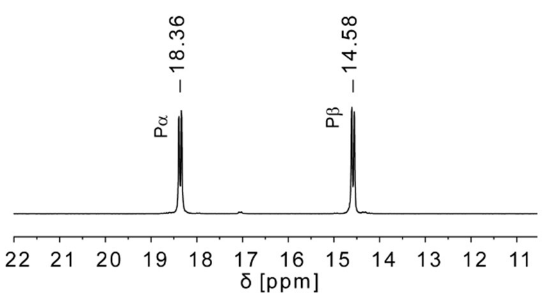

^{19}**F NMR** (376 MHz, D_2O) of **15**

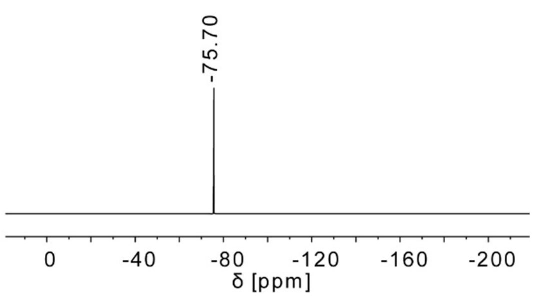

Supplementary Fig. 22: Structural characterization of *C*2-(5-trifluoroacetamidopent-1-yn-1-yl)-adenosine 5'-methylene bisphosphonate (15) by nuclear magnetic resonance spectroscopy (NMR).

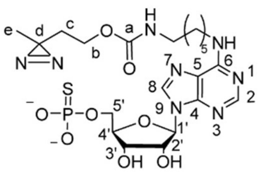

^{1}**H NMR** (400 MHz, MeOD) of **2**

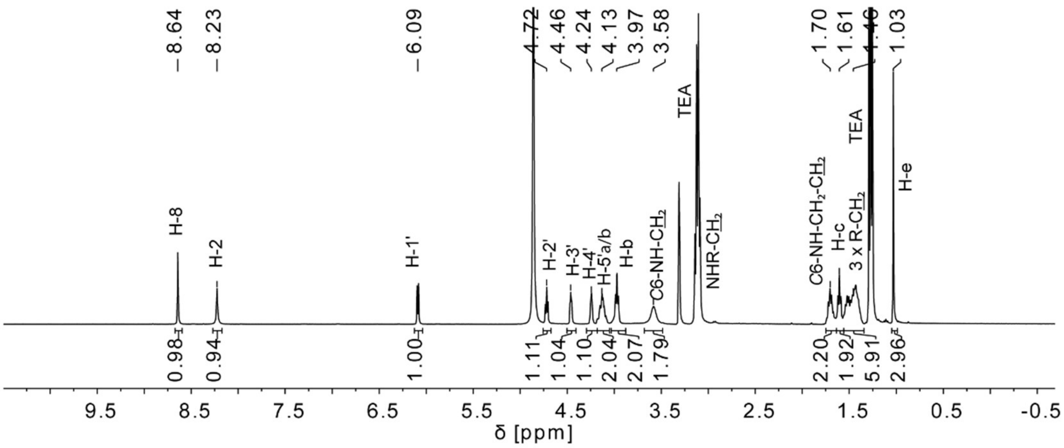

^{13}**C NMR** (126 MHz, MeOD) of **2**

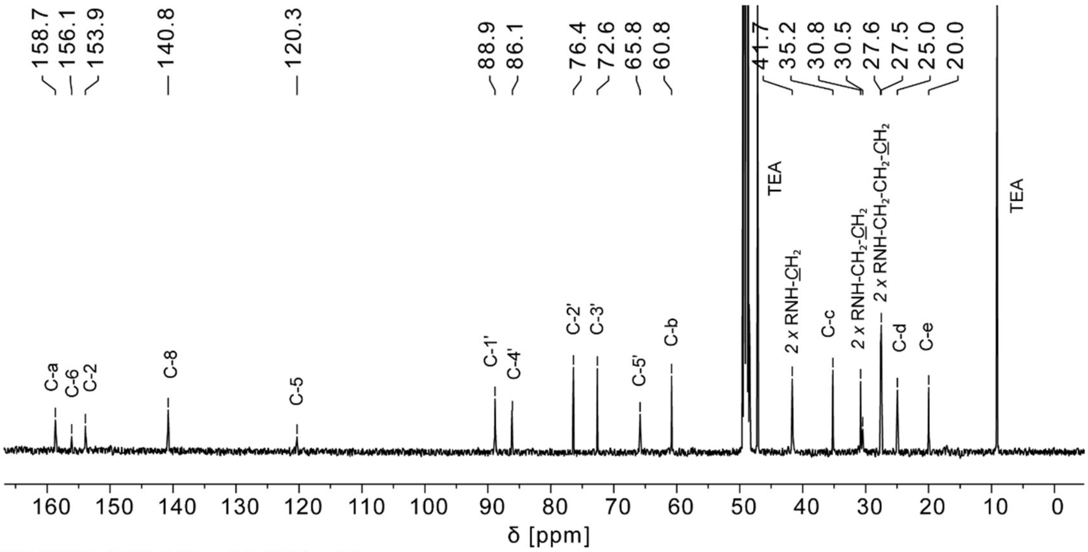

^{31}**P NMR** (162 MHz, MeOD) of **2**

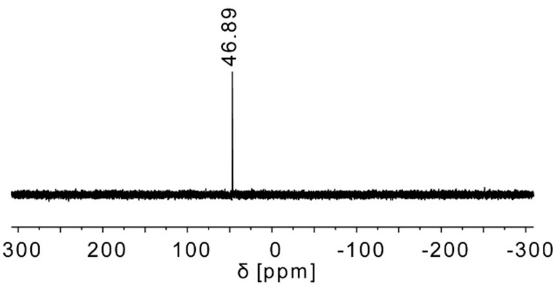

Supplementary Fig. 23: Structural characterization of *N*6-diazirine-adenosine 5'-thiomonophosphate (2) by nuclear magnetic resonance spectroscopy (NMR).

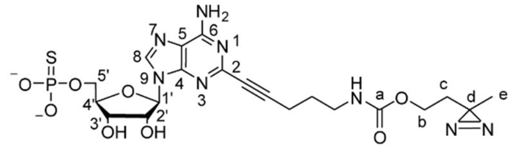

^{1}**H NMR** (400 MHz, MeOD) of **8**

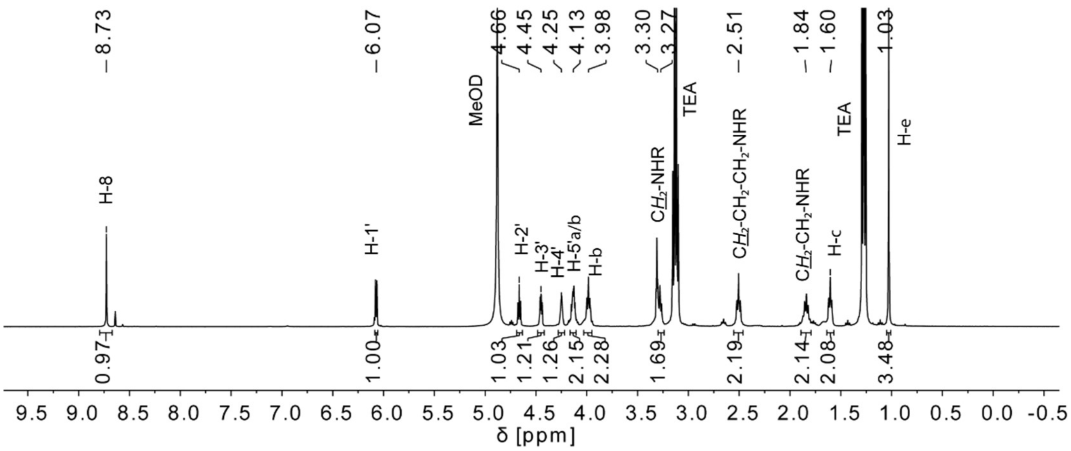

^{13}C **NMR** (101 MHz, MeOD) of **8**

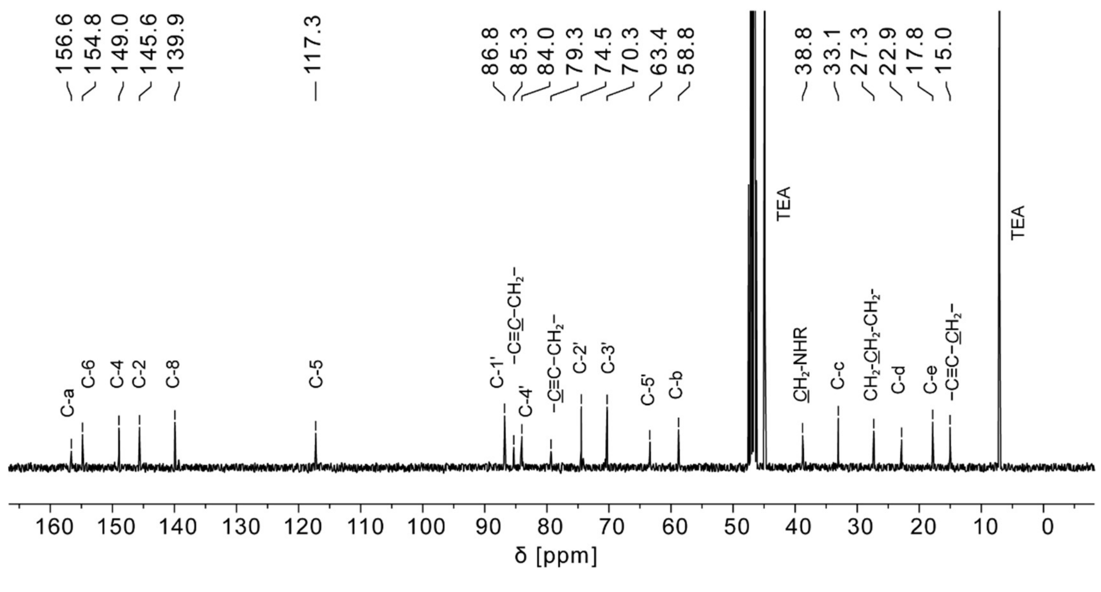

^{31}**P NMR** (162 MHz, MeOD) of **8**

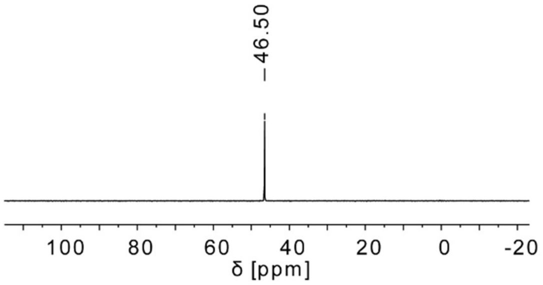

Supplementary Fig. 24: Structural characterization of *C*2-diazirine-adenosine 5'-thiomonophosphate (8) by nuclear magnetic resonance spectroscopy (NMR).

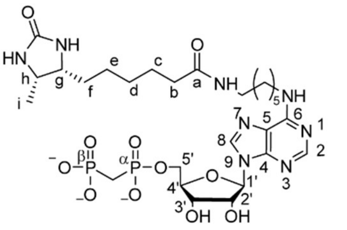

^{1}**H NMR** (400 MHz, MeOD) of **4**

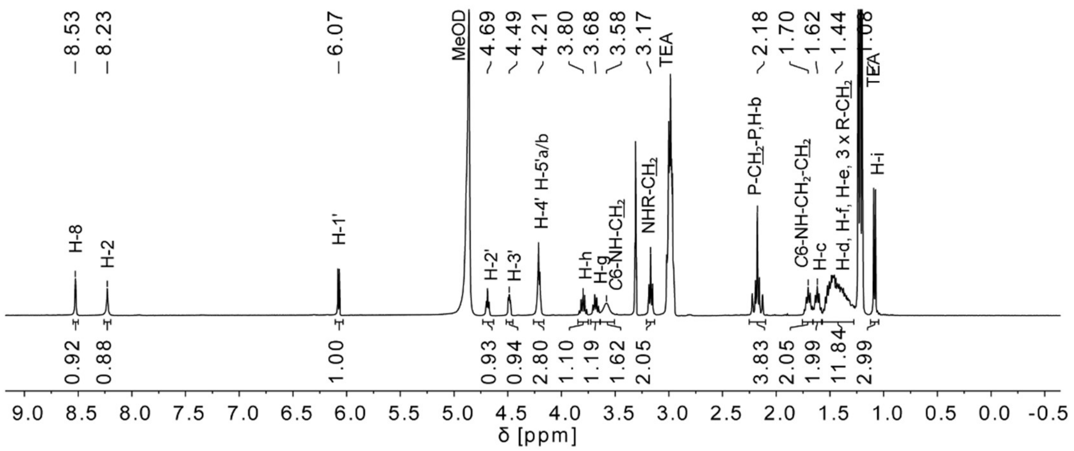

^{13}C **NMR** (126 MHz, MeOD) of **4**

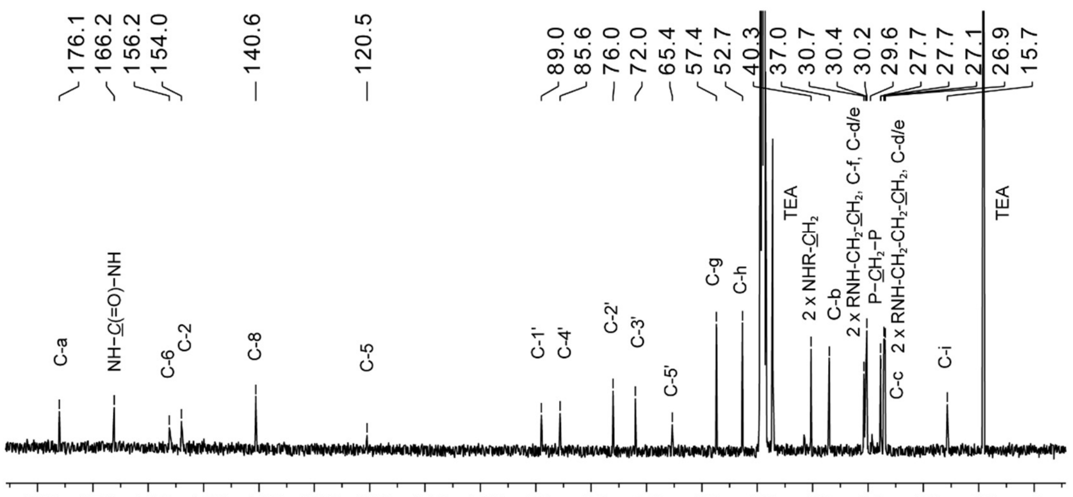

^{31}**P NMR** (162 MHz, MeOD) of **4**

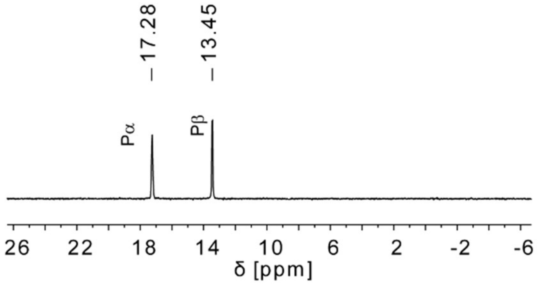

Supplementary Fig. 25: Structural characterization of *N*6-desthiobiotin-adenosine-5'-methylene bisphosphonate (4) by nuclear magnetic resonance spectroscopy (NMR).

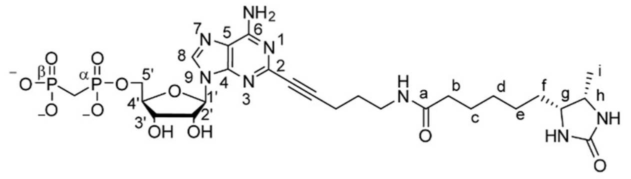

^{1}**H NMR** (400 MHz, MeOD) of **9**

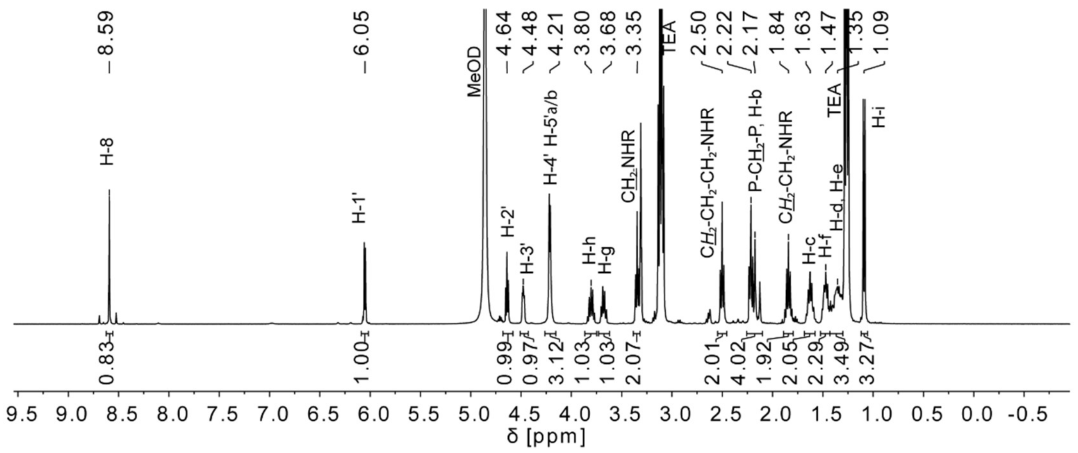

^{13}C **NMR** (101 MHz, MeOD) of **9**

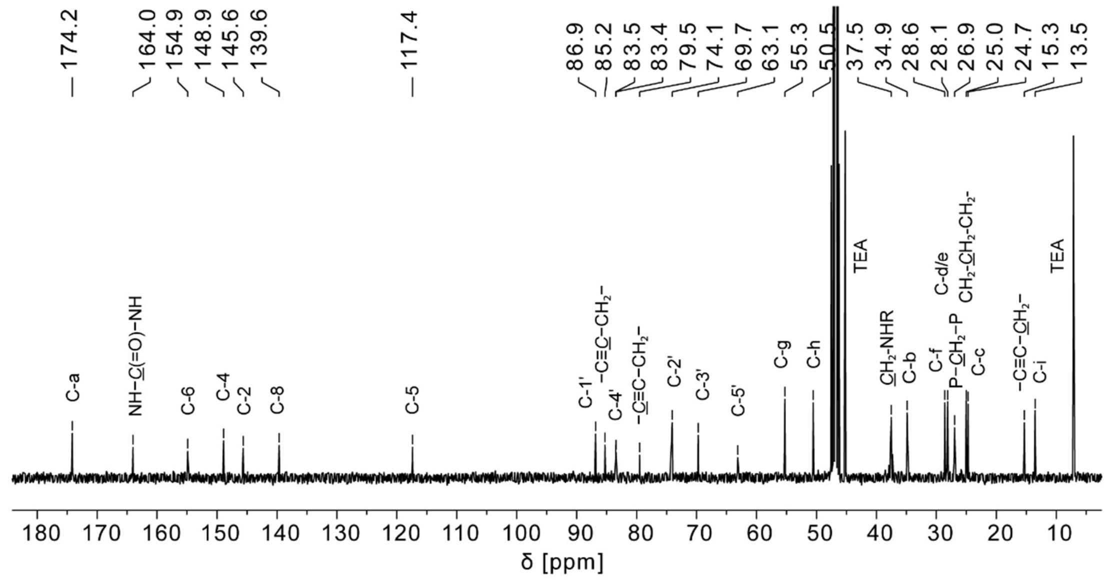

^{31}**P NMR** (162 MHz, MeOD) of **9**

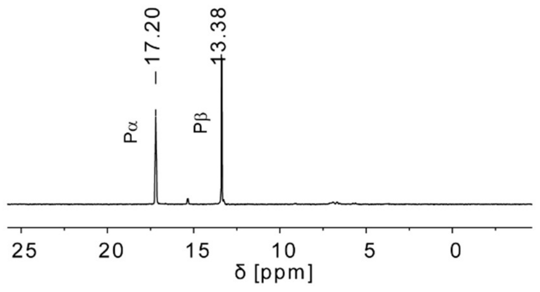

Supplementary Fig. 26: Structural characterization of *C*2-desthiobiotin-adenosine-5'-methylene bisphosphonate (9) by nuclear magnetic resonance spectroscopy (NMR).

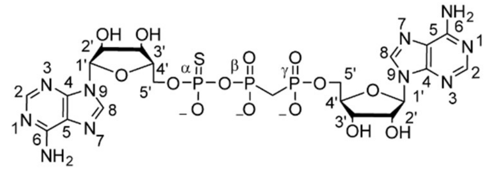

^{1}**H NMR** (400 MHz, MeOD) of comp-1

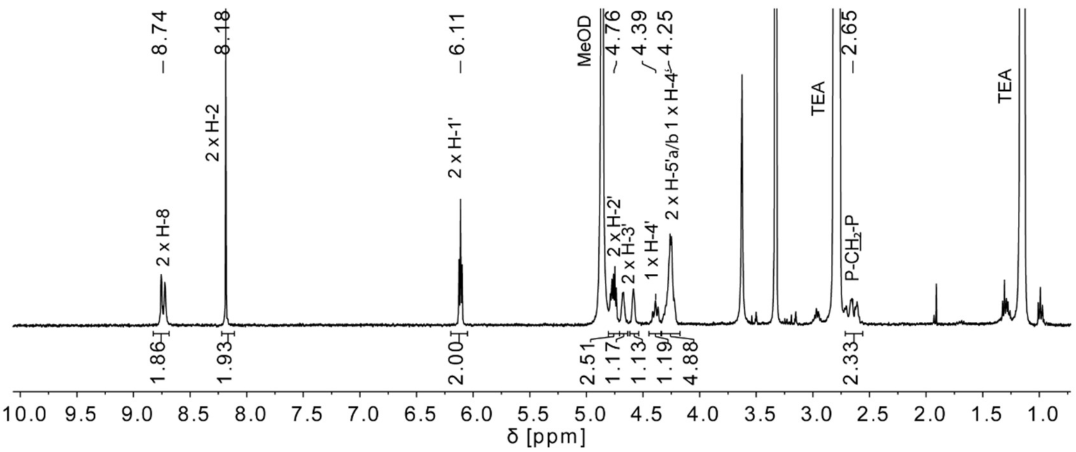

^{31}**P NMR** (162 MHz, MeOD) of comp-1

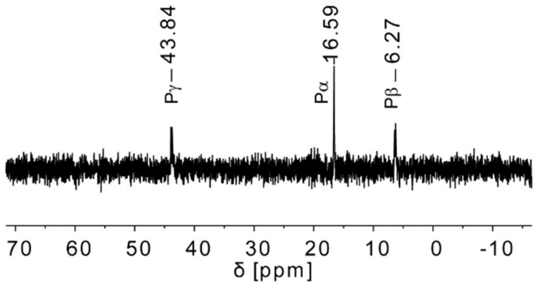

Supplementary Fig. 27: Structural characterization of comp-1 by nuclear magnetic resonance spectroscopy (NMR).

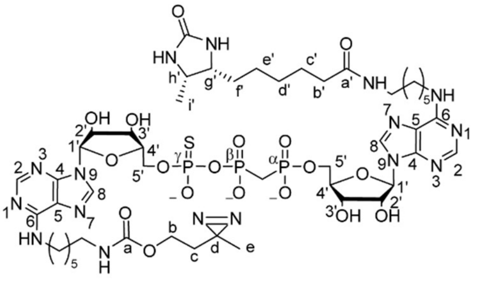

^{1}**H NMR** (400 MHz, MeOD) of PALP-1

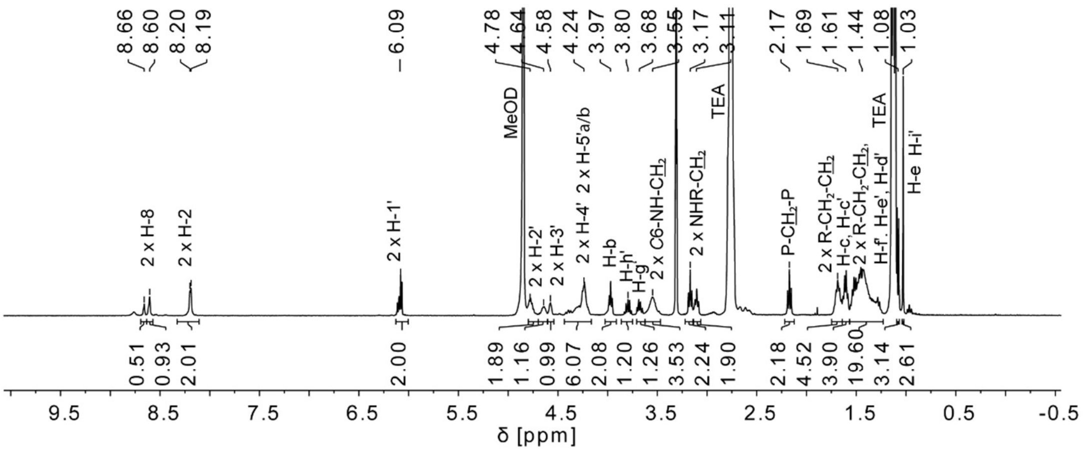

^{13}**C NMR** (201 MHz, MeOD) of PALP-1

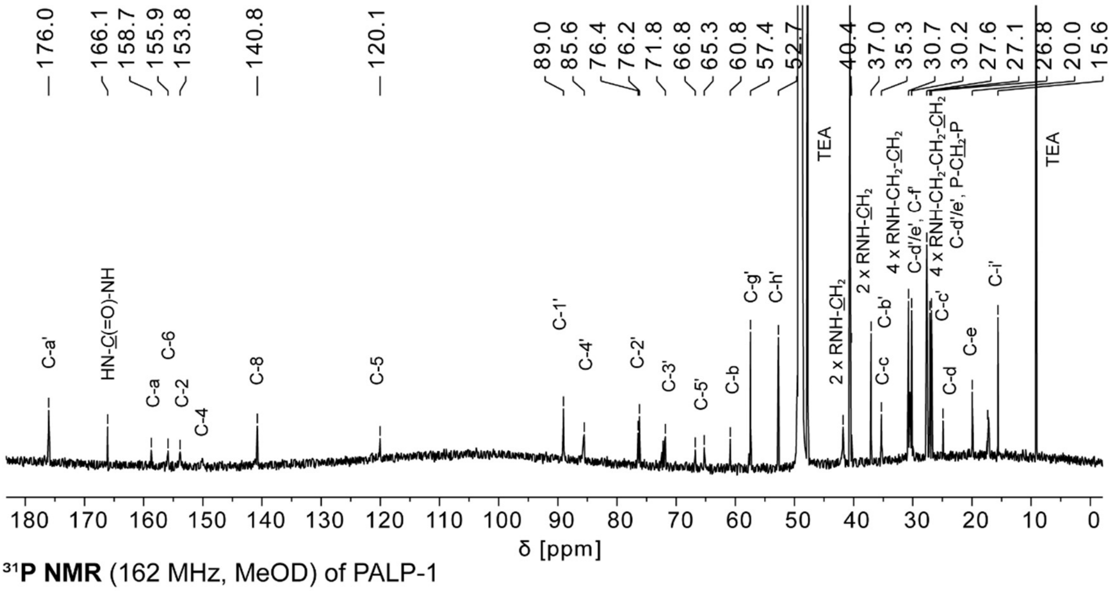

^{31}**P NMR** (162 MHz, MeOD) of PALP-1

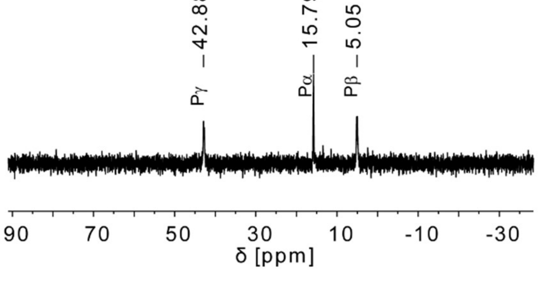

Supplementary Fig. 28: Structural characterization of PALP-1 by nuclear magnetic resonance spectroscopy (NMR).

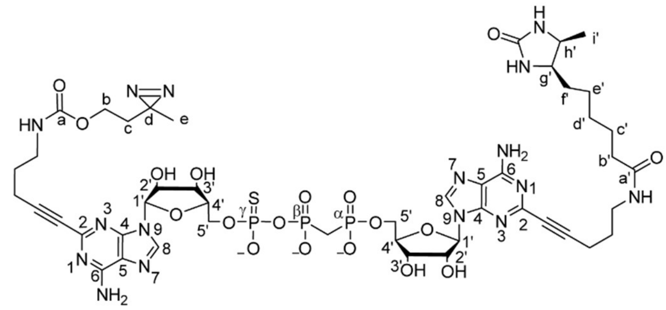

^{1}**H NMR** (800 MHz, MeOD) of PALP-3

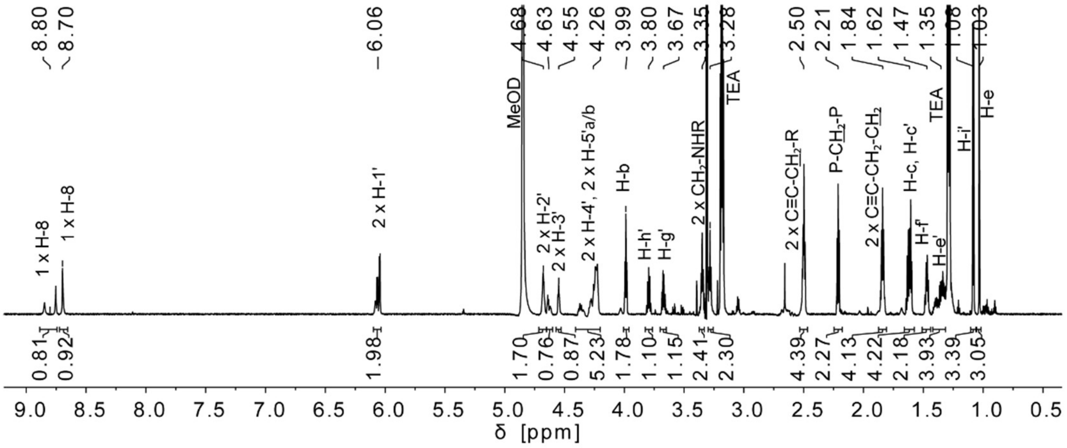

^{31}**P NMR** (324 MHz, MeOD) of PALP-3

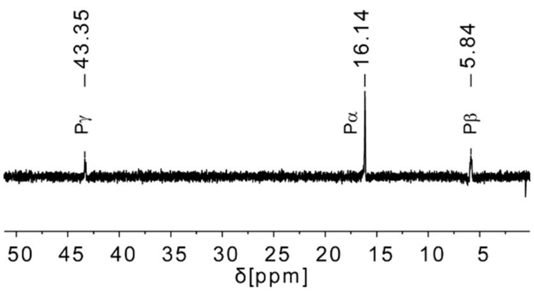

Supplementary Fig. 29: Structural characterization of PALP-3 by nuclear magnetic resonance spectroscopy (NMR).

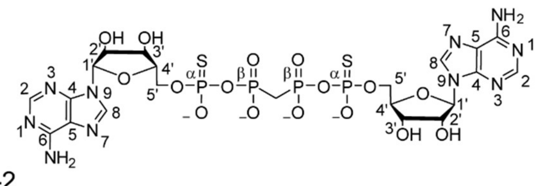

^{1}**H NMR** (400 MHz, D_2O) of comp-2

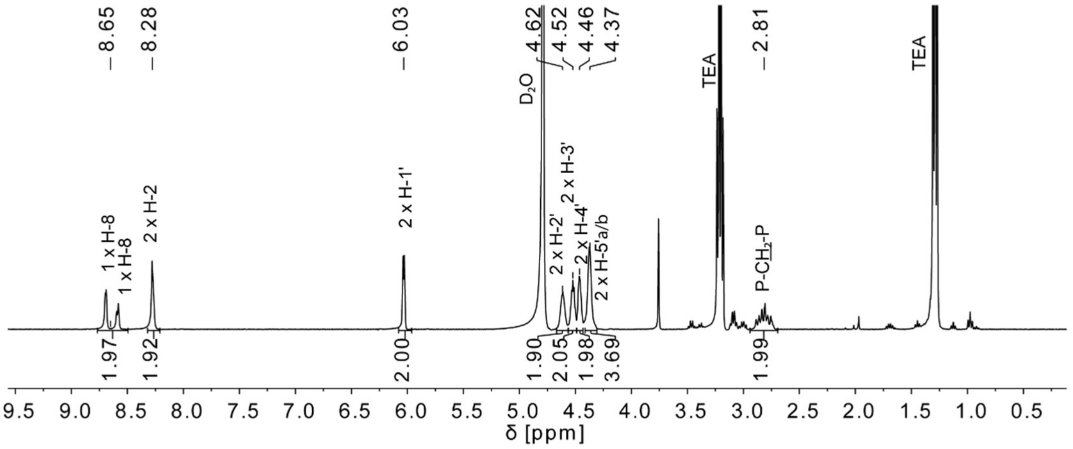

^{13}**C NMR** (151 MHz, D_2O) of comp-2

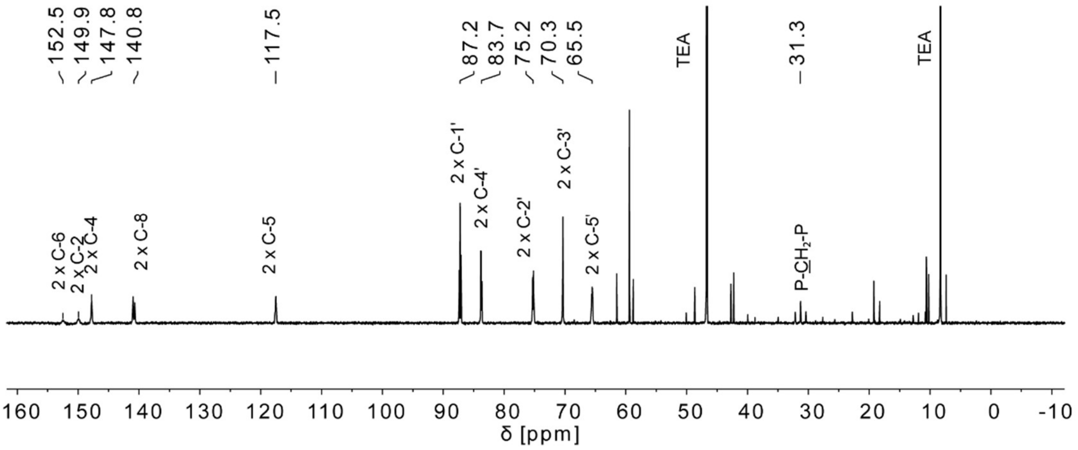

^{31}**P NMR** (162 MHz, D_2O) of comp-2

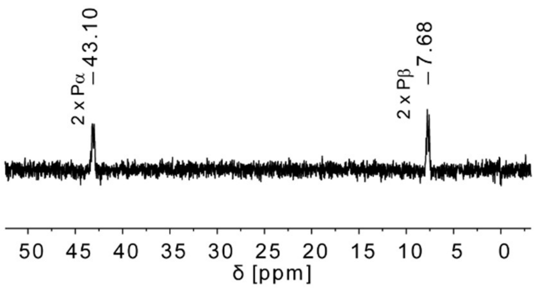

Supplementary Fig. 30: Structural characterization of comp-2 by nuclear magnetic resonance spectroscopy (NMR).

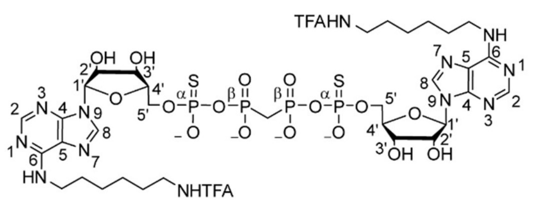

^{1}H NMR (400 MHz, D_2O) of **7**

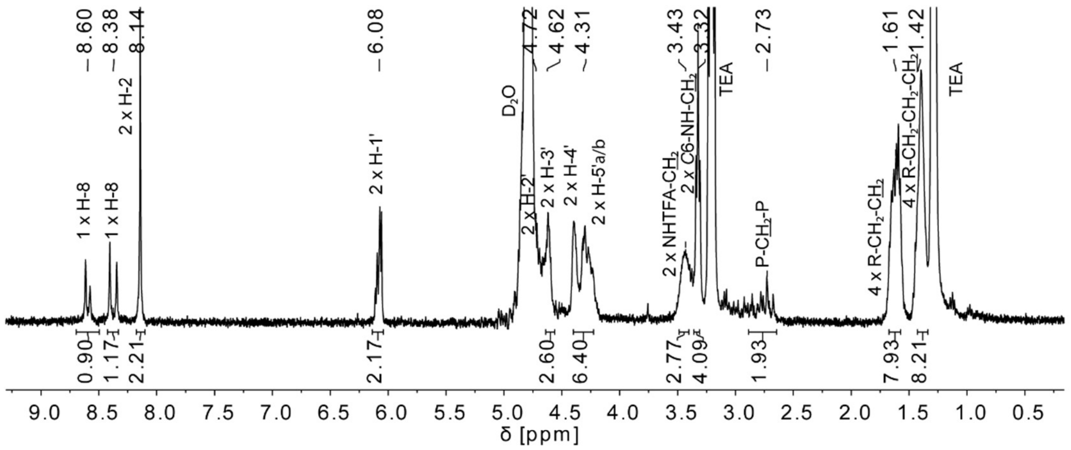

^{13}C NMR (151 MHz, D_2O) of **7**

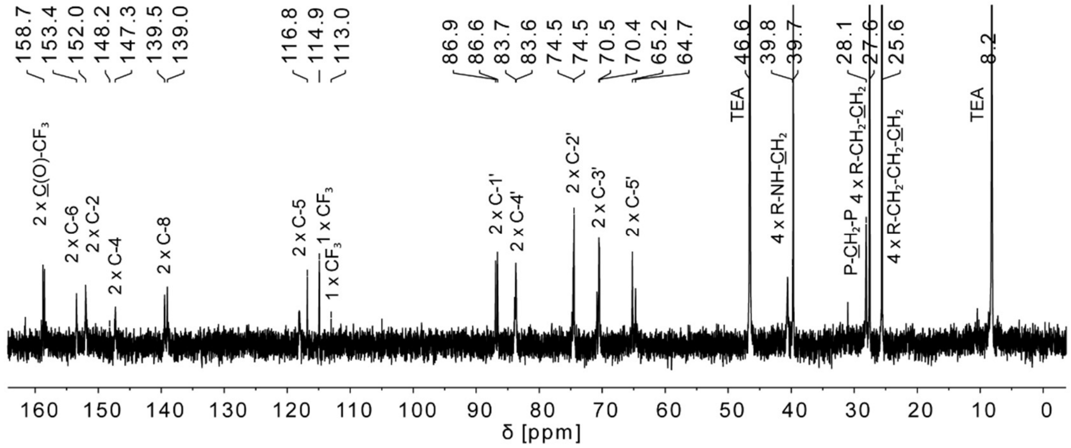

^{31}P NMR (162 MHz, D_2O) of **7**

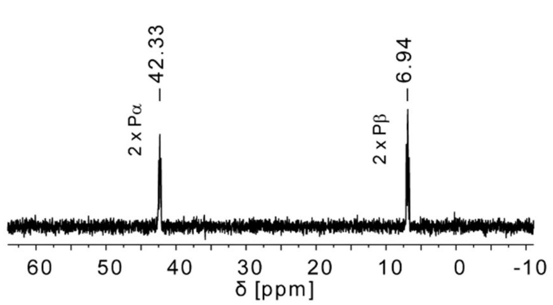

^{19}F NMR (376 MHz, D_2O) of **7**

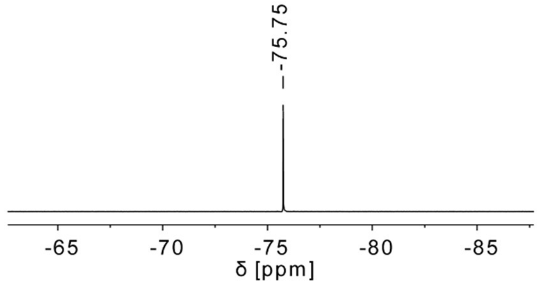

Supplementary Fig. 31: Structural characterization of *N*6-(6-trifluoroacetamidohexyl) adenosine Ap$_s$pCH$_2$pp$_s$A (7) by nuclear magnetic resonance spectroscopy (NMR).

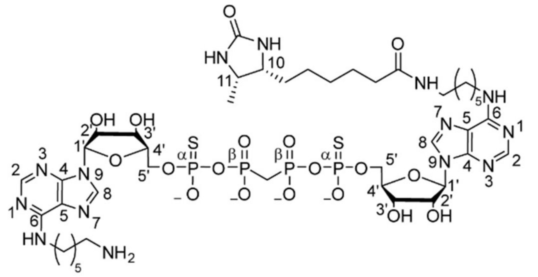

^{1}**H NMR** (400 MHz, D_2O) of **16**

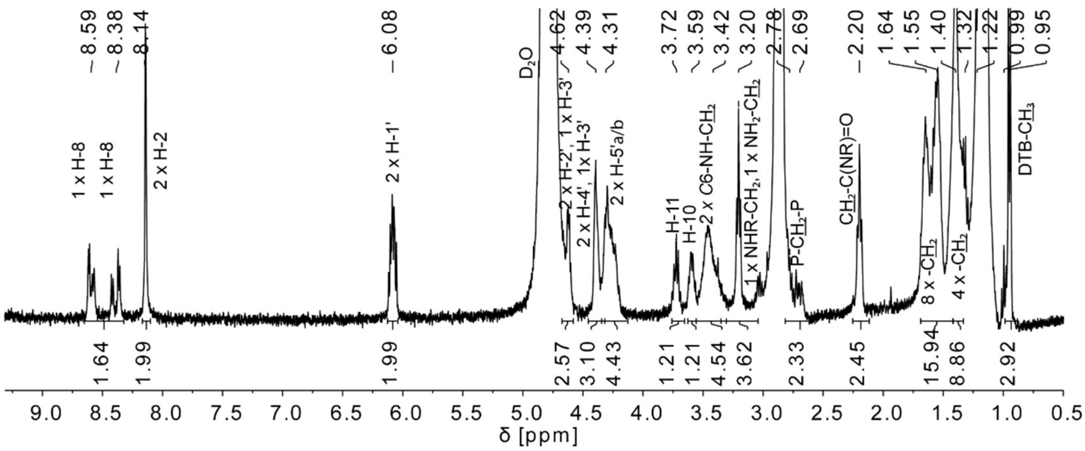

^{31}**P NMR** (162 MHz, D_2O) of **16**

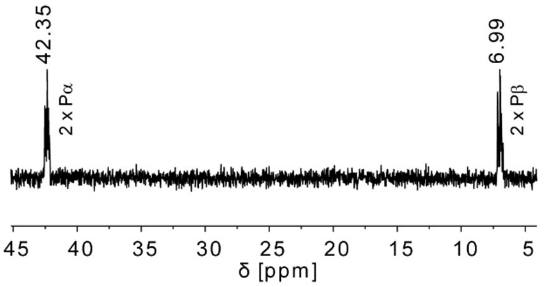

Supplementary Fig. 32: Structural characterization of compound 16 by nuclear magnetic resonance spectroscopy (NMR).

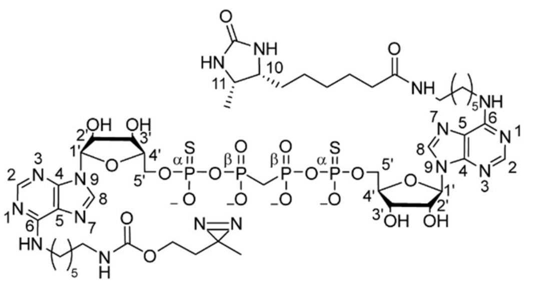

^{1}**H NMR** (400 MHz, D_2O) of PALP-2

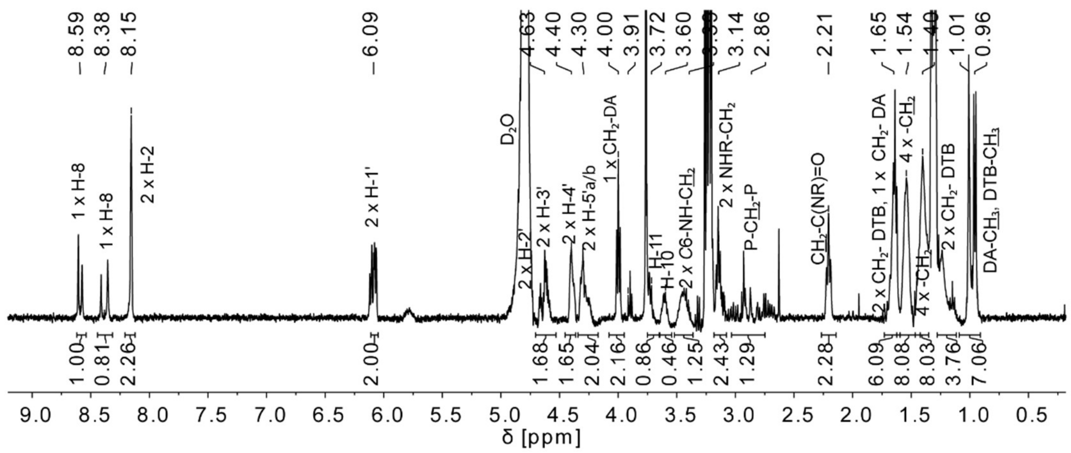

^{13}**C NMR** (151 MHz, D_2O) of PALP-2

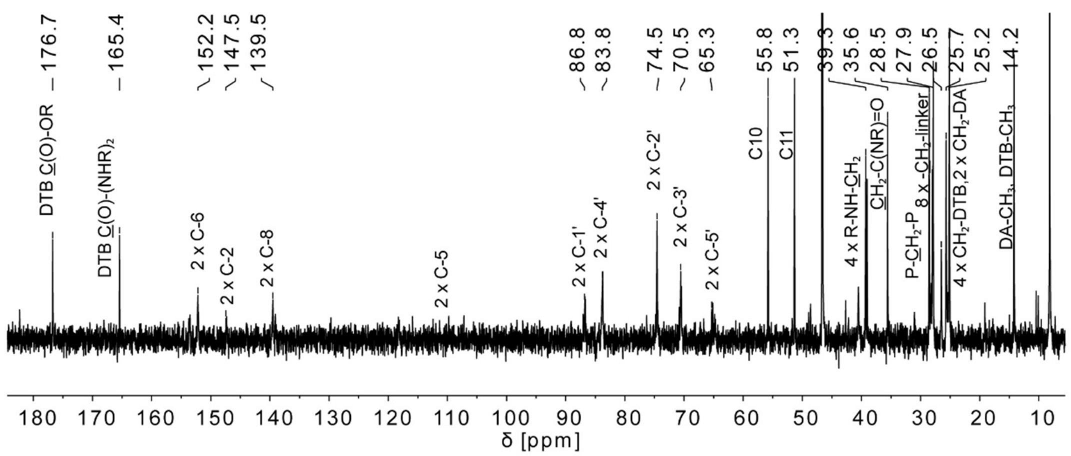

^{31}**P NMR** (162 MHz, D_2O) of PALP-2

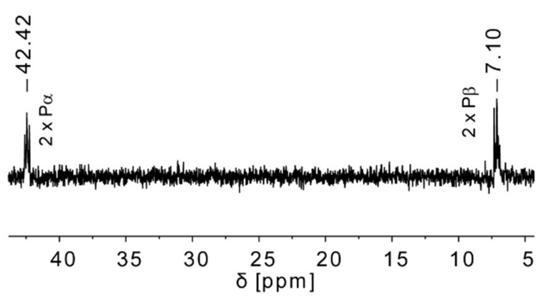

Supplementary Fig. 33: Structural characterization of PALP-2 by nuclear magnetic resonance spectroscopy (NMR).

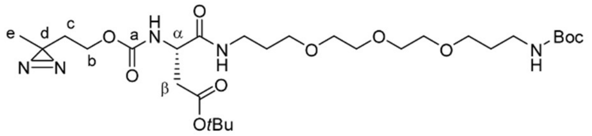

¹H NMR (400 MHz, DMSO) of **17**

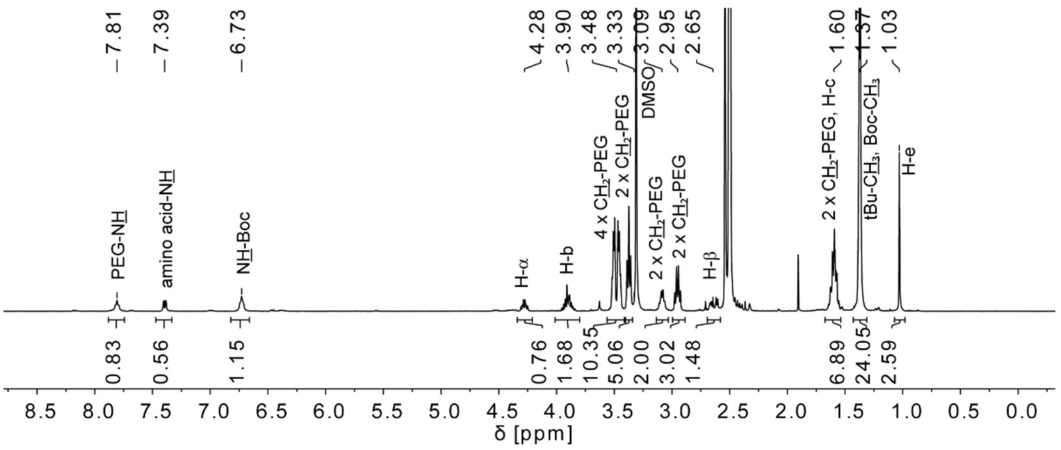

Supplementary Fig. 34: Structural characterization of control compound scaffold-precursor (17) by nuclear magnetic resonance spectroscopy (NMR).

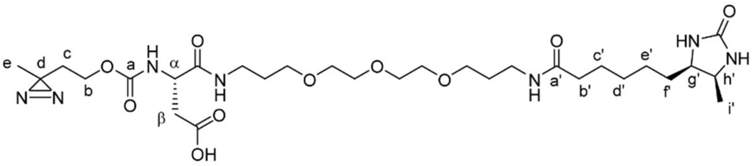

[1]**H NMR** (400 MHz, DMSO) of con-1

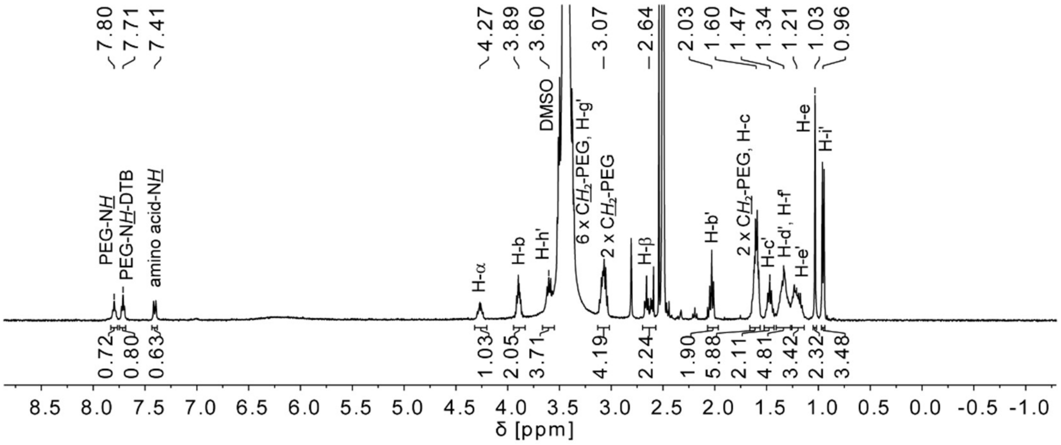

[13]**C NMR** (126 MHz, DMSO) of con-1

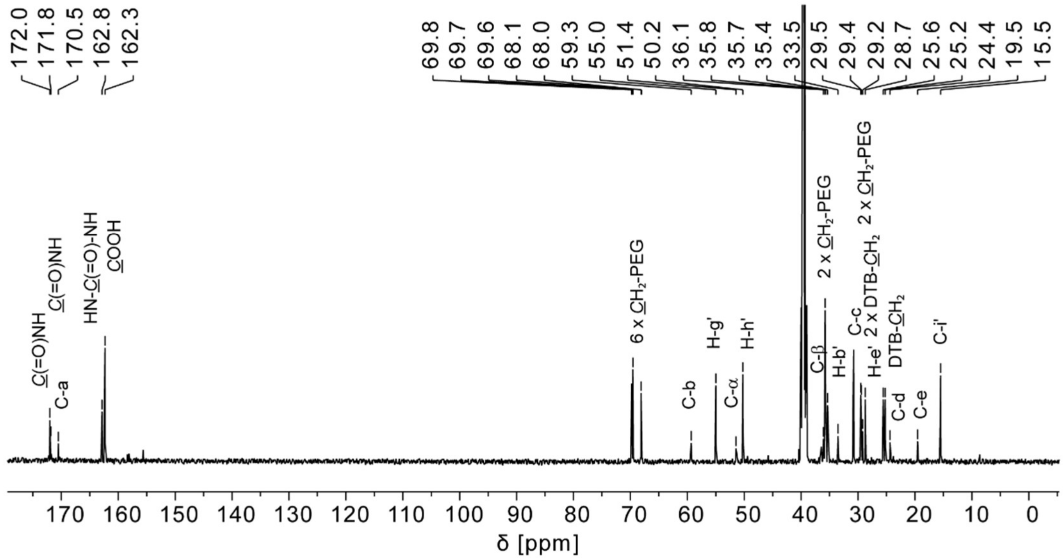

Supplementary Fig. 35: Structural characterization of con-1 by nuclear magnetic resonance spectroscopy (NMR).

Supplementary Tables

protein	K_D [µM]	B_{max} [mP]	R^2	assay window [mP]
**LDHA**	35.27 ± 0.69	323.30 ± 1.79	0.9992	290
**PGK1**	37.39 ± 1.62	220.90 ± 2.41	0.9950	221

Supplementary Table 1: Binding parameters obtained by FP-measurements with F-Ap_3A and LDHA or PGK1.

Time [min]	Solvent A [%]	Solvent B [%]
**0**	95	5
**2**	95	5
**7**	75	25
**22**	65	35
**25**	0	100
**30**	0	100
**35**	95	5
**40**	95	5

Supplementary Table 2: Gradient used for quantification of intracellular Ap_3A and Ap_4A via LC-HR-ESI-MS (solvent A: 10 mM NH_4OAc + 0.1% diethylamine (pH 10); solvent B: MeCN).

Time [min]	Solvent A [%]	Solvent B [%]
**0**	95	5
**5**	95	5
**25**	87.5	12.5
**28**	0	100
**33**	0	100
**37.5**	95	5
**40**	95	5

Supplementary Table 3: Gradient used for monitoring the stability of Ap_3A and Ap_4A in HEK293T cell lysates via analytical RP-HPLC (solvent A: 50 mM triethylammonium acetate buffer, pH 7.5; solvent B: MeCN).

Supplementary methods

General Experimental Procedures:

All reagents are commercially available and were used without further purification. Solvents were dried over molecular sieves as needed and used directly without further purification. Reactions were conducted under exclusion of air and moisture as needed. Purification of nucleotides by anion-exchange chromatography was performed on an Äkta purifier (GE Healthcare) using a DEAE Sephadex™ A-25 (GE Healthcare Bio-SciencesAB) column and a linear gradient of 0.1 M to 1.0 M triethylammonium bicarbonate buffer (TEAB, pH 7.5) or using a Dionex DNAPac PA-100 column and a linear gradient of eluent A (25 mM Tris·HCl and 5 % acetonitrile, pH 8.0) to eluent B (25 mM Tris·HCl, 1.0 M NaCl and 5 % acetonitrile, pH 8). Reversed phase high pressure liquid chromatography (RP-HPLC) was performed using a Shimadzu Prominence system having preparative LC-20AP pumps. For the purification of nucleotides, a VP 250/21 NUCLEODUR C18 HTec, 5 µm (Macherey-Nagel) column and a linear gradient of 5 % to 40 % acetonitrile in 50 mM TEAB were used. NMR spectra were recorded on a Bruker Avance III 400 MHz, Jeol Resonance ECZ 500R 500 MHz, Bruker Avance III 600 MHz or a Bruker AVANCE NEO 800 MHz spectrometer. NMR data was evaluated with MestReNova (version 14.1.2-25024). ^{1}H, ^{13}C, ^{19}F and ^{31}P chemical shifts are reported relative to the residual solvent peak and are given in ppm (δ). s: singlet, d: duplet, t: triplet, q: quartet, bs: broad signal, m: multiplet. HR-ESI-MS was measured on a Bruker Daltonics micrOTOF-Q II ESI-Q-TOF or a 6546 QTOF (Agilent) system and evaluated with Bruker Compass DataAnalysis (version 4.1) or MassHunter Qualitative Analysis (version 10.0.10305.0, Agilent). The reported yield refers to the analytically pure substance and is not optimized. *C*2-(5-Trifluoroacetamido-pent-1-yn-1-yl)-adenosine[3], (*i*Pr_2N)$_2$POFm[4], 1-methyl-3-benzenesulfonyl-imidazolium triflate[5], *N*6-(6-trifluoro-acetamidohexyl)-adenosine[3], diazirine-NHS[6] and *N*-Boc-*N*'-Asp(tBu)-4,7,10-trioxa-1,13-tridecanediamine[7] were synthesized according to corresponding literature.

Preparation of tetrabutylammonium salts of nucleotides

A column filled with CHELEX 100 ion-exchange resin (sodium form, 20 mL) was washed with water (500 mL) and loaded with tetrabutylammonium bromide (5.0 g, 15.5 mmol) dissolved in water (100 mL). The washing step was repeated once. The respective nucleotide as its triethylammonium salt was dissolved in water (10 mL) and applied to the column. After elution with water, the fractions containing the product were combined and the solvent was removed. The resulting nucleotide as tetrabutylammonium salt was freeze-dried and stored under nitrogen atmosphere at −20°C until use.

General procedure for adenosine 5'-monophosphates[3]

The adenosine derivative (1.0 eq) was dried *in vacuo* for 30 min and dissolved in OP(OMe)$_3$. The solution was cooled to 0 °C and $POCl_3$ (1.2–2.1 eq) was added. After stirring for 1.5 h at 0 °C, the reaction mixture was quenched by the addition of TEAB buffer (0.2 M) and stirred at room temperature for 30 min. The solution was washed thrice with ethyl acetate and the combined aqueous phases were evaporated *in vacuo*. The residue was purified by anion-exchange chromatography (DEAE Sephadex™ A-25) and RP-HPLC. Fractions containing the product were pooled, concentrated under reduced pressure and repeatedly freeze-dried to give the desired AMP derivative as triethylammonium salt.

$^{13}C_5$-Adenosine-5'-monophosphate (10)

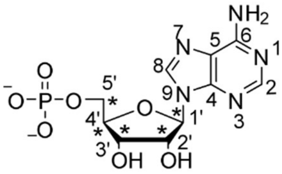

10

$^{13}C_5$-Adenosine (200 mg, 0.74 mmol, 1.0 eq) was reacted with $POCl_3$ (0.40 mg, 0.14 mL, 1.54 mmol, 2.1 eq) in $OP(OMe)_3$ (6 mL) following the general procedure (0.24 mmol, 33 %).

^{1}H NMR (600 MHz, D_2O) δ [ppm] = 8.43 (s, 1H, H-8), 8.07 (s, 1H, H-2), 6.02 (d, *J* = 5.6 Hz, 1H, H-1'), 4.70 (t, *J* = 5.3 Hz, 1H, H-2'), 4.45 (t, *J* = 4.0 Hz, 1H, H-3'), 4.34–4.28 (m, 1H, H-4'), 4.06–3.98 (m, 2H, H-5').

^{13}C NMR (151 MHz, D_2O) δ [ppm] = 155.2 (C-6), 152.5 (C-2), 148.8 (C-4), 139.9 (C-8), 118.3 (C-5), 86.9 (dd, *J* = 42.7, 3.6 Hz, C-1'), 84.3 (ddd, *J* = 42.7, 38.4, 8.7 Hz, C-4'), 74.4 (dd, *J* = 42.7, 37.7 Hz, C-2'), 70.5 (td, *J* = 38.1, 3.5 Hz, C-3'), 64.0 (dd, *J* = 42.8, 4.8 Hz, C-5').

^{31}P NMR (162 MHz, D_2O) δ [ppm] = 1.83 (s, 1P, P_α).

*C*2-(5-Trifluoroacetamidopent-1-yn-1-yl)-adenosine 5'-monophosphate (11)

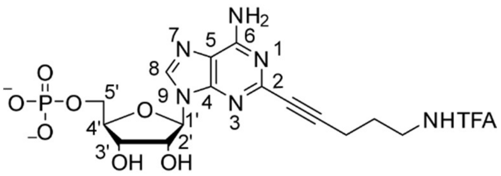

11

*C*2-(5-Trifluoroacetamido-pent-1-yn-1-yl)-adenosine[3] (400 mg, 0.90 mmol, 1.0 eq) was reacted with $POCl_3$ (0.17 mg, 0.10 mL, 1.08 mmol, 1.2 eq.) in $OP(OMe)_3$ (10 mL) following the general procedure (0.33 mmol, 37 %).

^{1}H NMR (400 MHz, D_2O) δ [ppm] = 8.43 (s, 1H, H-8), 5.86 (d, *J* = 5.4 Hz, 1H, H-1'), 4.59 (t, *J* = 5.3 Hz, 1H, H-2'), 4.39–4.30 (m, 1H, H-3'), 4.24–4.14 (m, 1H, H-4'), 3.92–3.79 (m, 2H, H-5'$_{a/b}$), 3.31 (t, *J* = 6.8 Hz, 2H, C$\underline{H_2}$-NHTFA), 2.36 (t, *J* = 7.0 Hz, 2H, C≡C−C$\underline{H_2}$), 1.74 (p, *J* = 6.9 Hz, 2H, C$\underline{H_2}$-CH_2-NHTFA).

^{13}C NMR (126 MHz, MeOD-d_4): δ [ppm] = 158.4 (only in 2D spectra, $\underline{C}$(O)-CF_3), 155.2 (C-6), 149.1 (C-4), 146.0 (C-2), 140.3 (C-8), 118.0 (C-5), 88.5 (C-1'), 87.1 (−C≡$\underline{C}$−CH_2−), 84.1 (C-4'), 79.2 (−$\underline{C}$≡C−CH_2−), 74.7 (C-2'), 70.4 (C-3'), 64.3 (C-5'), 39.0 (−$\underline{C}H_2$−NHTFA), 26.3 (CH_2−$\underline{C}H_2$−CH_2), 16.0 (−C≡C−$\underline{C}H_2$−).

^{31}P NMR (162 MHz, D_2O) δ [ppm] = 3.70 (s, 1P, P_α).

^{19}F NMR (376 MHz, D_2O) δ [ppm] = −75.78 (s, 3F, C$\underline{F_3}$).

HR-ESI-MS: *m/z* calculated: 523.0949 $[C_{17}H_{19}F_3N_6O_8P]^-$; *m/z* measured: 523.0969 $[C_{17}H_{19}F_3N_6O_8P]^-$. Deviation: 3.8 ppm.

$^{13}C_{10}$-*P*[1],*P*[3]-Diadenosine-5'-triphosphate ($^{13}C_{10}$-Ap$_3$A)

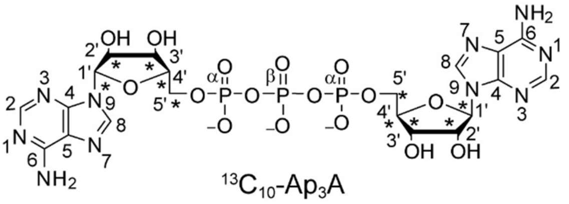

The synthesis of ^{13}C-labelled Ap$_3$A was performed by following a procedure published by Hofer *et al.*[4] $^{13}C_5$-Adenosine-5'-monophosphate (**10**) as its triethylammonium salt (107 µmol, 1.0 eq) was dissolved in dry dimethylformamide (DMF) (2 mL) and a solution of (iPr$_2$N)$_2$POFm[4] (36 mg, 75 µmol, 0.7 eq) in dry DMF (3 mL) was added. The reaction was started by addition of 5-phenyltetrazole (44 mg, 300 µmol, 2.8 eq) and the reaction mixture was stirred for 1 h. The reaction progress was monitored by ^{31}P and ^{1}H NMR. After completion, *m*CPBA (32 mg, 182 µmol, 1.7 eq) was added and the solution was stirred for 45 min. Et_2O/hexane (5:1, 36 mL) was added to the reaction mixture resulting in the formation of a white precipitate. The suspension was centrifuged (5 min, 4000 rpm) and the precipitate was dried *in vacuo* and dissolved in dry DMF (6 mL). To this solution piperidine (315 µL) was added and the mixture was stirred for 1 h. Afterwards, Et_2O (30 mL) was added, resulting in a white precipitate. After the subsequent centrifugation (5 min, 4000 rpm), the crude product was dried *in vacuo*. Purification by RP-HPLC and repeated freeze-drying gave the desired product as triethylammonium salt (22.9 µmol, 43%).

^{1}H NMR (600 MHz, D_2O) δ [ppm] = 8.26 (s, 2H, H-8), 8.03 (s, 2H, H-2), 5.94 (d, J = 4.4 Hz, 2H, H-1'), 4.55 (t, J = 4.6 Hz, 2H, H-2'), 4.44 (t, J = 4.5 Hz, 2H, H-3'), 4.34–4.20 (m, 6H, H-4', H-5').

^{13}C NMR (151 MHz, D_2O) δ [ppm] = 154.3 (C-6), 151.8 (C-2), 148.1 (C-4), 139.4 (C-8), 117.7 (C-5), 87.2 (dd, J = 42.7, 3.6 Hz, C-1'), 83.1 (ddd, J = 42.7, 38.4, 8.7 Hz, C-4'), 74.8 (dd, J = 42.7, 37.7 Hz, C-2'), 69.6 (td, J = 38.1, 3.5 Hz, C-3'), 64.7 (dd, J = 42.8, 4.8 Hz, C-5').

^{31}P NMR (162 MHz, D_2O) δ [ppm] = −11.57 (d, J = 16.2 Hz, 2P, 2 × P_α), −23.10 (t, J = 19.7 Hz, 1P, P_β).

HR-ESI-MS *m/z* calculated: 765.1082 [$C_{10}{}^{13}C_{10}H_{26}N_{10}O_{16}P_3$]$^-$; *m/z* measured: 765.1085 [$C_{10}{}^{13}C_{10}H_{26}N_{10}O_{16}P_3$]$^-$. Deviation: 0.39 ppm.

$^{13}C_{10}$-*P*[1],*P*[4]-Diadenosine-5'-tetraphosphate ($^{13}C_{10}$-Ap$_4$A)

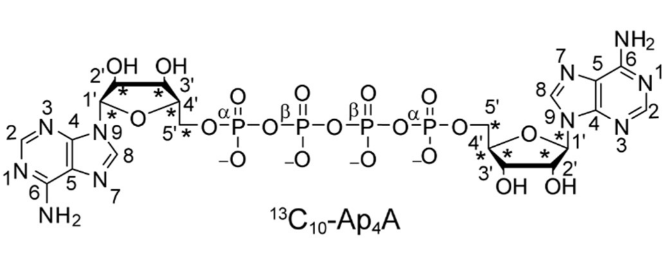

The synthesis of ^{13}C labelled Ap$_4$A was performed by following a procedure published by Yanachkov *et al.*[2] Pyrophosphate as tributylammonium salt (18 mg, 32.5 µmol, 0.5 eq) was coevaporated with dry DMF (3 x 1.5 mL) and dissolved in dry DMF (1 mL). Carbonyldiimidazole (CDI) (16 mg, 97.5 µmol, 1.5 eq) and dry Et_3N (4.5 µL, 32.5 µmol, 0.5 eq) were added and the mixture stirred for 5 h. $^{13}C_5$-Adenosine-5'-monophosphate (**10**) as tetrabutylammonium salt (65 µmol, 1.0 eq) and dry zinc chloride (44 mg, 325 µmol, 5.0 eq) were coevaporated separately with dry DMF (3 x 1.5 mL) and dissolved in dry DMF (1 mL each). All solutions were combined and the mixture concentrated under reduced pressure to a total volume of approximately 1 mL. Tetrazole (0.45 M in MeCN, 133 µL, 60 µmol, 0.9 eq) was

added and the resulting white suspension was stirred for 48 h. The reaction was quenched with ethylenediaminetetraacetic acid (EDTA) (0.5 M in water, 15 mL) and the resulting clear solution was evaporated under reduced pressure. The crude product was successively purified by anion-exchange chromatography (Dionex DNAPac PA-100) and RP-HPLC (pyramid column) resulting in the desired product as its triethyl ammonium salt (8.7 µmol, 27%).

^{1}H NMR (600 MHz, D_2O) δ [ppm] = 8.43 (s, 2H, H-8), 8.16 (s, 2H, H-2), 5.98 (d, *J* = 5.4 Hz, 2H, H-1'), 4.64 (t, *J* = 5.2 Hz, 2H, H-2'), 4.55–4.47 (m, 2H, H-3'), 4.38–4.32 (m, 2H, H-4'), 4.30–4.20 (m, 4H, H-5').

^{13}C NMR (151 MHz, D_2O) δ [ppm] = 153.7 (C-6), 151.0 (C-2), 148.3 (C-4), 140.2 (C-8), 117.9 (C-5), 86.9 (d, *J* = 42.7, C-1'), 83.8 (ddd, *J* = 42.4, 38.4, 9.3 Hz, C-4'), 74.7 (dd, *J* = 42.8, 37.6 Hz, C-2'), 70.2 (t, *J* = 38.1, C-3'), 65.2 (dd, *J* = 42.5, 5.4 Hz, C-5').

^{31}P NMR (162 MHz, D_2O) δ [ppm] = −11.26 (m, 2P, 2 × P_α), −22.99 (m, 2P, 2 × P_β).

HR-ESI-MS *m/z* calculated: 845.0745 $[C_{10}{}^{13}C_{10}H_{27}N_{10}O_{19}P_4]^-$; *m/z* measured: 845.0739 $[C_{10}{}^{13}C_{10}H_{27}N_{10}O_{19}P_4]^-$. Deviation: 0.71 ppm.

*C*2-(5-Trifluoroacetamido-pent-1-yn-1-yl)-adenosine-5'-(adenosine-5')-triphosphate (12)

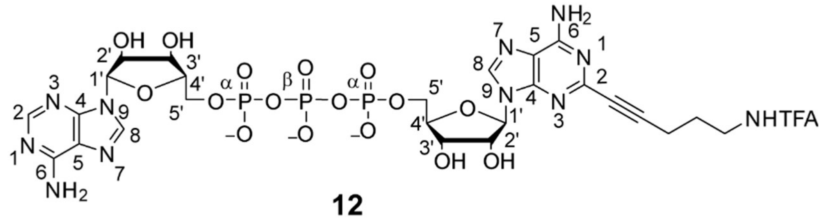

12

The synthesis of Ap_3A analogue **12** was performed based on a procedure published by Mohamady *et al.*[5] Compound **11** (100 µmol, 1.0 eq) was dissolved in dry DMF (2.4 mL) and diisopropylethylamine (50 µL, 300 µmol, 3.0 eq) was added. 1-Methyl-3-benzenesulfonyl-imidazolium triflate[5] (48 mg, 130 µmol, 1.3 eq) was added and the reaction mixture was stirred at room temperature for 3 min. This solution was added dropwise to a solution of the tetrabutylammonium salt of ADP (135 mg, 150 µmol, 1.50 eq) and $ZnCl_2$ (14 mg, 100 µmol, 1.0 eq) in dry DMF at 0 °C. The reaction mixture was stirred at 0 °C for 1.5 h and quenched with 50 m M TEAB (5 mL, pH 7.5) and EDTA (0.25 m M) at room temperature. The mixture was washed with EtOAc (3 × 5 mL) and the combined organic phases were again washed with water (5 mL). The combined aqueous phases were evaporated under reduced pressure. The residue was purified via anion-exchange chromatography (DEAE Sephadex™ A-25) and RP-HPLC. Fractions containing the desired product were pooled, concentrated under reduced pressure and repeatedly freeze-dried. The product (5 µmol, 5 %) was obtained as its triethylammonium salt.

^{1}H NMR (400 MHz, D_2O) δ [ppm] = 8.35 (s, 1H, 1 × H-8), 8.28 (s, 1H, 1 × H-8), 8.14 (s, 1H, H-2), 5.99 (dd, *J* = 8.4, 4.7 Hz, 2H, 2 × H-1'), 4.62 (t, *J* = 4.8 Hz, 2H, 2 × H-2'), 4.51 (td, *J* = 4.8, 1.7 Hz, 2H, 2 × H-3'), 4.41–4.25 (m, 6H, 2 × H-4', 2 × $H\text{-}5'_{a/b}$), 3.57 (t, *J* = 6.7 Hz, 2H, $C\underline{H_2}$-NHTFA), 2.60 (t, *J* = 6.9 Hz, 2H, C≡C−$C\underline{H_2}$), 1.99 (dd, *J* = 8.6, 5.1 Hz, 2H, $C\underline{H_2}$-CH_2-NHTFA).

^{31}P NMR (162 MHz, D_2O) δ [ppm] = −11.58 (d, *J* = 19.6 Hz, 2P, 2 × P_α), −23.07 (t, *J* = 19.3 Hz, 1P, P_β).

^{19}F NMR (376 MHz, D_2O) δ [ppm] = −75.72 (s, 3F, $C\underline{F_3}$).

HR-ESI-MS: *m/z* calculated: 932.1137 $[C_{27}H_{31}F_3N_{11}O_{17}P_3]^-$; *m/z* measured: 932.1136 $[C_{27}H_{31}F_3N_{11}O_{17}P_3]^-$. Deviation: 0.1 ppm.

Deprotection

*C*2-modified Ap_3A analog **12** (4.9 µmol, 1.0 eq) was deprotected by dissolving the compound in aqueous NH_3 (3 %, 5 mL) and stirring the solution at 0 °C for 3 h. The solvents were removed under reduced pressure and the residue was purified via RP-HPLC. After repeated freeze-drying the deprotected compound (2.0 µmol, 40 %) was obtained as triethylammonium salt.

*C*2-(5-FAM)-Ap_3A (F-Ap_3A)

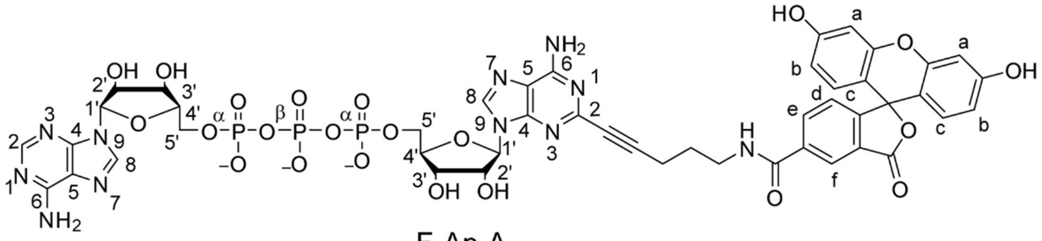

F-Ap_3A

Deprotected *C*2-(5-amino-pent-1-yn-1-yl)-adenosine-5')-adenosine-5')-triphosphate (2.40 µmol, 1.0 eq) was dissolved in 0.1 M aqueous $NaHCO_3$ (1 mL). 5-Carboxyfluorescein-NHS-ester (5.50 µmol, 2.0 eq) dissolved in DMF (0.20 mL) was added. The pH value was adjusted to 8.7 and reaction mixture was stirred at room temperature overnight. The crude product was concentrated under reduced pressure and purified via flash column chromatography (silica gel, *i*PrOH/H_2O/NH_3 6/1/1–3/1/1) and RP-HPLC. Fractions containing the desired product were pooled and concentrated under reduced pressure. After repeated freeze-drying, the triethylammonium salt of the product (0.15 µmol, 6 %) was obtained.

^{1}H NMR (500 MHz, D_2O) δ [ppm] = 8.22 (t, *J* = 2.6 Hz, 3H, 2 × H-8, H-2), 8.08 (d, *J* = 2.0 Hz, 1H, H-f), 8.04–7.95 (m, 1H, H-e), 7.30 (d, *J* = 7.9 Hz, 1H, H-d), 7.09 (d, *J* = 9.0 Hz, 1H, H-c), 7.00 (d, *J* = 9.0 Hz, 1H, H-c), 6.74 (s, 2H, H-b, H-b), 6.66 (dd, *J* = 24.1, 9.4 Hz, 2H, H-a, H-a), 5.97–5.88 (m, 2H, 2 × H-1'), 4.56 (t, *J* = 4.3 Hz, 1H, 1 × H-2'), 4.52 (t, *J* = 4.9 Hz, 1H, 1 × H-2'), 4.50–4.36 (m, 2H, 2 × H-3'), 4.35–4.14 (m, 6H, 2 × H-4', 2 × H-5'$_{a/b}$), 3.74–3.65 (m, 2H, C*H₂*−NHR), 2.70 (t, *J* = 6.6 Hz, 2H, C≡C−C*H₂*), 2.13–2.02 (m, 2H, CH_2−C*H₂*−CH_2).

^{31}P NMR (162 MHz, D_2O) δ [ppm] = −10.87–−11.15 (m, 2P, P_α), −22.44 (t, *J* = 19.6 Hz, 1P, P_β).

HR-ESI-MS: *m/z* calculated: 1194.1791 $[C_{46}H_{43}N_{11}O_{22}P_3]^-$; *m/z* measured: 1194.1796 $[C_{46}H_{43}N_{11}O_{22}P_3]^-$. Deviation: 0.4 ppm.

General procedure for adenosine 5'-thiomonophosphates[8]

The adenosine derivative (1.0 eq) was dried *in vacuo* for 30 min and dissolved in dry pyridine. The solution was cooled to 0 °C and $PSCl_3$ (1.2–2.0 eq) was added. After stirring for 2 h at 0 °C, the reaction mixture was quenched by the addition of TEAB buffer (0.2 M) and stirred at room temperature for 30 min. The solvents were removed under reduced pressure and the residue was purified by anion-exchange chromatography (DEAE Sephadex™ A-25) and RP-HPLC. Fractions containing the product were pooled, concentrated under reduced pressure and repeatedly freeze-dried to give the desired product as triethylammonium salt.

Adenosine 5'-thiomonophosphate (1)

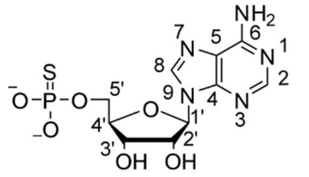

1

Adenosine (0.20 g, 0.75 mmol, 1.0 eq) was reacted with $PSCl_3$ (0.15 g, 0.09 mL, 0.90 mmol, 1.2 eq) in dry pyridine (15 mL) following the general procedure (0.20 mmol, 26 %).

^{1}H NMR (400 MHz, D_2O): δ [ppm] = 8.65 (s, 1H, H-8), 8.18 (s, 1H, H-2), 6.11 (d, J = 6.0 Hz, 1H, H-1'), 4.83–4.80 (m, 1H, H-2'), 4.54 (dd, J = 5.1, 3.3 Hz, 1H, H-3'), 4.41–4.39 (m, 1H, H-4'), 4.10 (dd, J = 5.9, 3.2 Hz, 2H, H-5'$_{a/b}$).

^{13}C NMR (101 MHz, D_2O): δ [ppm] = 155.3 (C-6), 152.5 (C-2), 149.0 (C-4), 140.4 (C-8), 118.4 (C-5), 86.9 (C-1'), 84.7 (C-4'), 74.6 (C-2'), 70.5 (C-3'), 64.0 (C-5').

^{31}P NMR (162 MHz, D_2O): δ [ppm] = 43.629 (s, 1P, P_α).

HR-ESI-MS: *m/z* calculated: 362.0330 $[C_{10}H_{13}N_5O_6PS]^-$; *m/z* measured: 362.0327 $[C_{10}H_{13}N_5O_6PS]^-$. Deviation: 0.9 ppm.

*N*6-(6-Trifluoroacetamidohexyl)-adenosine 5'-thiomonophosphate (5)

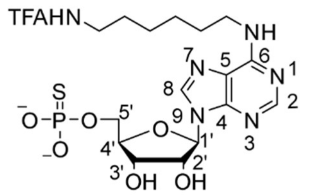

5

*N*6-(6-Trifluoroacetamidohexyl)-adenosine[3] (0.40 g, 0.87 mmol, 1.0 eq) was reacted with $PSCl_3$ (0.17 g, 0.10 mL, 1.04 mmol, 1.2 eq) in dry pyridine (15 mL) following the general procedure (0.25 mmol, 29 %).

^{1}H NMR (400 MHz, D_2O): δ [ppm] = 8.60 (s, 1H, H-8), 8.17 (s, 1H, H-2), 6.09 (d, J = 6.0 Hz, 1H, H-1'), 4.82–4.79 (m, 1H, H-2'), 4.54 (dd, J = 5.1, 3.3 Hz, 1H, H3'), 4.39 (dt, J = 4.4, 2.1 Hz, 1H, H-4'), 4.18–4.08 (m, 2H, H-5'$_{a/b}$), 3.48 (bs, 2H, C6-NH-C$\underline{H_2}$), 3.27 (t, J = 7.0 Hz, 2H, NHTFA-C$\underline{H_2}$), 1.63 (p, J = 7.0 Hz, 2H, C6-NH-CH_2-C$\underline{H_2}$), 1.53 (p, J = 7.1 Hz, 2H, NHTFA-CH_2-C$\underline{H_2}$), 1.46–1.33 (m, 4H, 2 × CH_2 linker).

^{13}C NMR (101 MHz, D_2O): δ [ppm] = 158.6 ($\underline{C}$(O)-CF_3), 154.4 (C-6), 152.7(C-2), 148.0 (only in 2D spectra, C-4), 139.6 (C-8), 117.4 (C-5), 114.6 (CF_3), 86.8 (C-1'), 84.6 (C-4'), 74.6 (C-2'), 71.0 (C-3'), 64.2 (C-5'), 39.8 (2 × RNH-$\underline{C}H_2$), 28.5 (C6-NH-CH_2-$\underline{C}H_2$), 27.7 (NHTFA-CH_2-$\underline{C}H_2$), 25.7(C6-NH-CH_2-CH_2-$\underline{C}H_2$), 25.7(NHTFA-CH_2-CH_2-$\underline{C}H_2$).

^{31}P NMR (162 MHz, D_2O): δ [ppm] = 45.12 (s, 1P, P_α).

^{19}F NMR (376 MHz, D_2O): δ [ppm] = −75.71 (s, 3F, C$\underline{F_3}$).

HR-ESI-MS: *m/z* calculated: 557.1201 $[C_{18}H_{25}F_3N_6O_7PS]^-$; *m/z* measured: 557.1203 $[C_{18}H_{25}F_3N_6O_7PS]^-$. Deviation: 0.4 ppm.

*C*2-(5-Trifluoroacetamidopent-1-yn-1-yl)-adenosine 5'-thiomonophosphate (13)

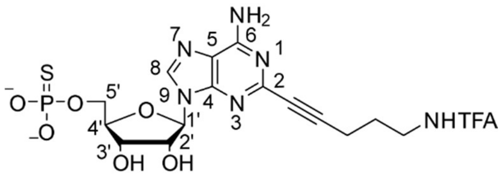

13

*C*2-(5-Trifluoroacetamidopent-1-yn-1-yl)-adenosine[3] (0.30 g, 0.68 mmol, 1.0 eq) was reacted with $PSCl_3$ (0.23 g, 0.15 µL, 1.35 mmol, 2.0 eq) in dry pyridine (17 mL) following the general procedure (0.26 mmol, 38 %).

^{1}H NMR (400 MHz, MeOD-d_4): δ [ppm] = 8.73 (s, 1H, 8−H), 6.07 (d, *J* = 5.8 Hz, 1H, 1'−H), 4.67 (t, *J* = 5.4 Hz, 1H, 2'−H), 4.45 (dd, *J* = 5.0, 3.2 Hz, 1H, 3'−H), 4.25 (dt, *J* = 4.5, 2.2 Hz, 1H, 4'−H), 4.20–4.07 (m, 2H, 5'−H), 3.47 (t, *J* = 7.0 Hz, 2H, C$\underline{H_2}$−NHTFA), 2.51 (t, *J* = 7.1 Hz, 2H, C≡C−C$\underline{H_2}$), 1.91 (p, *J* = 7.1 Hz, 2H, C$\underline{H_2}$-CH_2-NHTFA).

^{13}C NMR (101 MHz, MeOD-d_4): δ [ppm] = 156.7 (only in 2D spectra, $\underline{C}$(O)-CF_3), 154.8 (C-6), 149.0 (C-4), 145.5 (C-2), 140.0 (C-8), 117.3 (C-5), 86.8 (C-1'), 84.7 (−C≡$\underline{C}$−CH_2−), 84.2 (C-4'), 79.6 (−$\underline{C}$≡C−CH_2−), 74.4 (C-2'), 70.3 (C-3'), 63.3 (C-5'), 37.8 (−$\underline{C}H_2$−NHTFA), 26.3 (CH_2−$\underline{C}H_2$−CH_2), 15.1 (−C≡C−$\underline{C}H_2$−).

^{31}P NMR (162 MHz, MeOD-d_4): δ [ppm] = 47.59 (s, 1P, P_α).

^{19}F NMR (376 MHz, MeOD-d_4): δ [ppm] = −78.09 (s, 3F, C$\underline{F_3}$).

HR-ESI-MS: *m/z* calculated: 539.0731 $[C_{17}H_{19}F_3N_6O_7PS]^-$; *m/z* measured: 539.0728 $[C_{17}H_{19}F_3N_6O_7PS]^-$. Deviation: 0.4 ppm.

General procedure for adenosine 5'-methylene bisphosphonate derivatives[9]

The adenosine derivative (1.0 eq) was dried *in vacuo* for 30 min and suspended in dry $OP(OMe)_3$. The suspension was cooled to 0 °C and a solution of predried methylene bis(phosphonic dichloride) (2.0–3.0 eq) in $OP(OMe)_3$ was added slowly. After stirring for 4 h at 0 °C, the reaction mixture was quenched by the addition of TEAB buffer (0.2 M) and stirred at room temperature for 30 min. The solution was washed five times with ethyl acetate and the combined aqueous phases were evaporated *in vacuo*. The residue was purified by anion-exchange chromatography (DEAE Sephadex™ A-25) and RP-HPLC. Fractions containing the product were pooled, concentrated under reduced pressure and repeatedly freeze-dried to give the desired product as triethylammonium salt.

Adenosine 5'-methylene bisphosphonate (3)

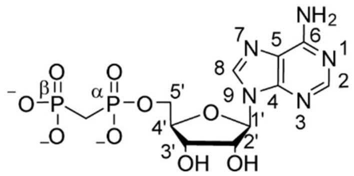

3

Adenosine (0.20 g, 0.75 mmol, 1.0 eq) and methylene bis(phosphonic dichloride) (0.56 g, 2.24 mmol, 3.0 eq) were reacted in dry $OP(OMe)_3$ (20 mL) following the general procedure. (0.26 mmol, 35 %).

^{1}H NMR (400 MHz, D_2O): δ [ppm] = 8.48 (s, 1H, H-8), 8.13 (s, 1H, H-2), 6.05 (d, *J* = 5.5 Hz, 1H, H-1'), 4.74 (t, *J* = 5.5 Hz, 1H, H-2'), 4.52 (dd, *J* = 5.2, 3.9 Hz, 1H, H-3'), 4.39–4.32 (m, 1H, H-4'), 4.16 (dd, *J* = 5.5, 3.3 Hz, 2H, H-5'$_{a/b}$), 2.18 (t, *J* = 19.7 Hz, 2H, C*H*$_2$).

^{13}C NMR (101 MHz, D_2O): δ [ppm] = 154.9 (C-6), 152.1 (C-2), 148.7 (C-4), 140.0 (C-8), 118.3 (C-5), 86.5 (C-1'), 84.0 (C-4'), 74.2 (C-2'), 70.3 (C-3'), 63.5 (C-5'), 27.5 (*C*H$_2$).

^{31}P NMR (162 MHz, D_2O): δ [ppm] = 18.30 (d, *J* = 10.2 Hz, 1P, P_α), 14.72 (d, *J* = 10.2 Hz, 1P, P_β).

HR-ESI-MS: *m*/*z* calculated: 424.0418 $[C_{11}H_{16}N_5O_9P_2]^-$; *m*/*z* measured: 424.0236 $[C_{11}H_{16}N_5O_9P_2]^-$. Deviation: 4.3 ppm.

*N*6-(6-Trifluoroacetamidohexyl)-adenosine 5'-methylene bisphosphonate (14)

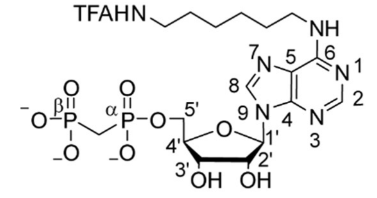

14

*N*6-(6-trifluoroacetamidohexyl)-adenosine[3] (0.30 g, 0.65 mmol, 1.0 eq) was reacted with methylene bis(phosphonic dichloride) (0.38 g, 1.30 mmol 2.0 eq) in dry $OP(OMe)_3$ (15 mL) according to the general procedure (0.23 mmol, 43 %).

^{1}H NMR (400 MHz, D_2O): δ [ppm] = 8.40 (s, 1H, H-8), 8.06 (s, 1H, H-2), 5.99 (d, *J* = 5.7 Hz, 1H, H-1'), 4.70 (t, *J* = 5.4 Hz, 1H, H-2'), 4.48 (t, *J* = 4.4 Hz, 1H, H-3'), 4.30 (q, *J* = 3.8 Hz, 1H, H-4'), 4.11 (t, *J* = 4.5 Hz, 2H, H-5'$_{a/b}$), 3.50–3.25 (m, 2H, *C*6-NH-C$\underline{H_2}$), 3.17 (t, *J* = 7.0 Hz, 2H, NHTFA-C*H*$_2$), 2.15 (t, *J* = 19.8 Hz, 2H, P-C*H*$_2$-P), 1.51 (p, *J* = 7.1 Hz, 2H, C6-NH-CH$_2$-C*H*$_2$), 1.47–1.34 (m, 2H, NHTFA-CH$_2$-C*H*$_2$), 1.28–1.21 (m, 4H, NHTFA-CH$_2$-CH$_2$-C*H*$_2$, C6-NH-CH$_2$-CH$_2$-C*H*$_2$).

^{13}C NMR (101 MHz, D_2O): δ [ppm] = 158.5 (q, *J* = 37.0 Hz, *C*(O)-CF_3), 154.2 (C-6), 152.6 (C-2), 147.7 (C-4), 139.2 (C-8), 117.4 (C-5), 114.5 (CF_3), 86.8 (C-1'), 84.0 (C-4'), 74.2 (C-2'), 70.4 (C-3'), 63.7 (C-5'), 40.6 (C6-NH-*C*H$_2$), 39.6 (NHTFA-CH$_2$), 28.4 (P-*C*H$_2$-P), 27.7(linker-CH$_2$), 26.7(linker-CH$_2$), 26.4(linker-CH$_2$), 25.7 (linker-CH$_2$).

^{31}P NMR (162 MHz, D_2O): δ [ppm] = 18.21 (d, *J* = 9.2 Hz, P_α), 14.66 (d, *J* = 9.2 Hz, P_β).

^{19}F NMR (376 MHz, D_2O): δ [ppm] = −75.74 (3F, C*F*$_3$).

*C*2-(5-Trifluoroacetamidopent-1-yn-1-yl)-adenosine 5'-methylene bisphosphonate (15)

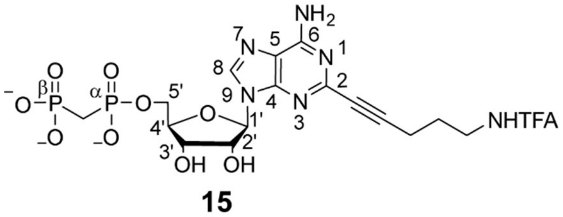

15

*C*2-(5-Trifluoroacetamidopent-1-yn-1-yl)-adenosine[3] (0.50 g, 1.13 mmol, 1.0 eq) was reacted with methylene bis(phosphonic dichloride) (0.56 g, 2.25 mmol, 2.0 eq) in dry $OP(OMe)_3$ (20 mL) according to the general procedure (0.47 mmol, 42 %).

^{1}H NMR (400 MHz, D_2O): δ [ppm] = 8.45 (s, 1H, H-8), 5.98 (d, J = 5.5 Hz, 1H, H-1'), 4.67 (d, J = 5.3 Hz, 1H, H-2'), 4.49 (d, J = 4.7 Hz, 1H, H-3'), 4.31 (d, J = 4.1 Hz, 1H, H-4'), 4.21 – 4.03 (m, 2H, H-5'$_{a/b}$), 3.44 (t, J = 6.9 Hz, 2H, C$\underline{H_2}$-NHTFA), 2.48 (t, J = 7.0 Hz, 2H, C≡C−C$\underline{H_2}$), 2.16 (t, J = 19.7 Hz, 2H, P-C$\underline{H_2}$-P), 1.86 (p, J = 7.0 Hz, 2H, C$\underline{H_2}$-CH_2-NHTFA).

^{13}C NMR (101 MHz, D_2O): δ [ppm] = 158.8 (q, J = 37.4 Hz, $\underline{C}$(O)-CF_3), 154.9 (C-6), 148.9 (C-2), 145.7 (C-4), 140.3 (C-8), 117.5 (C-5), 114.5 (*CF_3*), 88.3 (C≡$\underline{C}$-CH_2-), 87.0 (C-1'), 83.8 (C-4'), 79.1 ($\underline{C}$≡C-CH_2-), 74.5 (C-2'), 70.2 (C-3'), 63.6 (C-5'), 38.8 ($\underline{C}H_2$-NHTFA), 27.7 (P-$\underline{C}H_2$-P), 26.4 ($\underline{C}H_2$-CH_2-NHTFA), 15.9 (C≡C-$\underline{C}H_2$-).

^{31}P NMR (162 MHz, D_2O): δ [ppm] = 18.36 (d, J = 8.6 Hz, 1P, P_α), 14.58 (d, J = 8.6 Hz, 1P, P_β).

^{19}F NMR (376 MHz, D_2O): δ [ppm] = −75.70 (s, 3F, C$\underline{F}_3$).

HR-ESI-MS: *m/z* calculated: 601.0830 $[C_{18}H_{22}F_3N_6O_{10}P_2]^-$; *m/z* measured: 601.0829 $[C_{18}H_{22}F_3N_6O_{10}P_2]^-$. Deviation: 0.2 ppm.

Modification of nucleotide building blocks[3]

Deprotection of *N*6-modified TFA protected nucleotides

The protected nucleotide (**5** or **14**, 1.0 eq) was dissolved in H_2O. Aqueous NH_3 solution (25 %, final concentration 10 %) was added and the reaction mixture stirred for 3 h at room temperature. After concentration *in vacuo*, the residue was purified with RP-HPLC. Repeated freeze drying yielded the deprotected nucleotide as its triethylammonium salt.

Deprotection of *C*2-modified TFA protected nucleotides

The protected nucleotide (**13** or **15**, 1.0 eq) was dissolved in H_2O and cooled to 0 °C. Aqueous NH_3 solution (25 %, final concentration 3 %) was added and the reaction mixture stirred for 3 h at 0 °C. The ammonia was removed under reduced pressure at 0 °C. After concentration *in vacuo*, the residue was purified with RP-HPLC. Repeated freeze drying yielded the deprotected nucleotide as its triethylammonium salt.

Coupling with diazirine

The deprotected nucleotide (1.0 eq) was dissolved in aqueous 0.1 M $NaHCO_3$ solution (pH 8.7) and reacted with diazirine-NHS[6] (2.0 eq) dissolved in DMF overnight at room temperature. The solvents were removed under reduced pressure und the residue purified by RP-HPLC. After repeated freeze drying, the product was obtained as its triethylammonium salt.

*N*6-Diazirine-adenosine 5'-thiomonophosphate (2)

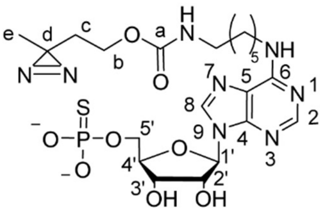

2

*N*6-(6-Aminohexyl)-adenosine 5'-thiomonophosphate (0.15 mmol, 1.0 eq) was coupled with diazirine-NHS[6] (0.07 g, 0.30 mmol, 2.0 eq) in aqueous $NaHCO_3$ solution (0.1 M, 10 mL, pH 8.7), following the general procedure. The product was obtained as a white powder as its triethylammonium salt (98 µmol, 66 %).

^{1}H NMR (400 MHz, MeOD-d_4): δ [ppm] = 8.64 (s, 1H, H-8), 8.23 (s, 1H, H-2), 6.09 (d, J = 5.9 Hz, 1H, H-1'), 4.72 (t, J = 5.5 Hz, 1H, H-2'), 4.46 (t, J = 3.9 Hz, 1H, H-3'), 4.24 (d, J = 3.5 Hz, 1H, H-4'), 4.19–4.07 (m, 2H, H-5'$_{a/b}$), 3.97 (t, J = 6.3 Hz, 2H, H-b), 3.67–3.49 (m, 2H, *C*6-NH-C*H₂*), 3.33–3.28 (2H, NHR-C*H₂*, behind solvent peak), 1.70 (p, J = 7.2 Hz, 2H, *C*6-NH-CH_2-C*H₂*), 1.61 (t, J = 6.3 Hz, 2H, H-c), 1.57–1.35 (m, 6H, NHR-CH_2-C*H₂*-C*H₂*-C*H₂*), 1.03 (s, 3H, H-e).

^{13}C NMR (126 MHz, MeOD-d_4): δ [ppm] = 158.7 (C-a), 156.1 (C-6), 153.9 (C-2), 148.0 (only in 2D spectra, C-4), 140.8 (C-8), 120.3 (C-5), 88.9 (C-1'), 86.1 (C-4'), 76.4 (C-2'), 72.6 (C-3'), 65.8 (C-5'), 60.8 (C-b), 41.7 (2 × RNH-*C*H₂), 35.2 (C-c), 30.8 (*C*6-NH-CH_2-*C*H₂), 30.5 (DA-NH-CH_2-*C*H₂), 27.6 (RNH-CH_2-CH_2-*C*H₂), 27.5 (RNH-CH_2-CH_2-*C*H₂), 25.0 (C-d), 20.0 (C-e).

^{31}P NMR (162 MHz, MeOD-d_4): δ [ppm] = 46.89 (s, 1P, P_α).

HR-ESI-MS: *m/z* calculated: 587.1807 $[C_{21}H_{32}N_8O_8PS]^-$; *m/z* measured: 587.1805 $[C_{21}H_{32}N_8O_8PS]^-$. Deviation: 0.3 ppm.

*C*2-Diazirine-adenosine 5'-thiomonophosphate (8)

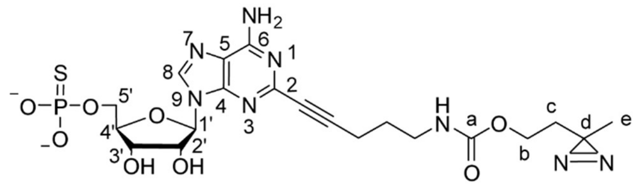

8

*C*2-(5-Aminopent-1-yn-1-yl)-adenosine 5'-thiomonophosphate (0.09 mmol, 1.0 eq) was coupled with diazirine-NHS[6] (0.05 g, 0.19 mmol, 2.0 eq) in aqueous $NaHCO_3$ solution (0.1 M, 10 mL, pH 8.7), following the general procedure. The product was obtained as a white powder as its triethylammonium salt (68 µmol, 72 %).

^{1}H NMR (400 MHz, MeOD-d_4): δ [ppm] = 8.73 (s, 1H, H-8), 6.07 (d, J = 5.6 Hz, 1H, H-1'), 4.66 (t, J = 5.3 Hz, 1H, H-2'), 4.45 (dd, J = 5.1, 3.4 Hz, 1H, H-3'), 4.25 (dt, J = 3.9, 2.0 Hz, 1H, H-4'), 4.19–4.07 (m, 2H, H-5'$_{a/b}$), 3.98 (t, J = 6.3 Hz, 2H, H-b), 3.28 (t, J = 6.5 Hz, 2H, C*H₂*−NHR, partially behind solvent peak), 2.51 (t, J = 7.1 Hz, 2H, C≡C−C*H₂*), 1.84 (p, J = 6.9, 2H, CH_2−C*H₂*−CH_2), 1.60 (t, J = 6.3 Hz, 2H, H-c), 1.03 (s, 3H, H-e).

^{13}C NMR (101 MHz, MeOD-d_4): δ [ppm] = 156.6 (C-a), 154.8 (C-6), 149.0 (C-4), 145.6 (C-2), 139.9 (C-8), 117.3 (C-5), 86.8 (C-1'), 85.3 (−C≡*C*−CH_2−), 84.0 (C-4'), 79.3 (−*C*≡C−CH_2−), 74.5 (C-2'), 70.3 (C-3'), 63.4 (C-5'), 58.8 (C-b), 38.8 (−*C*H₂−NHR), 33.1 (C-c), 27.3 (CH_2−*C*H₂−CH_2), 22.9 (C-d), 17.8 (C-e), 15.0 (−C≡C−*C*H₂−).

^{31}P NMR (162 MHz, MeOD-d_4): δ [ppm] = 46.51 (s, 1P, P_α).

HR-ESI-MS: *m/z* calculated: 569.1337 $[C_{20}H_{26}N_8O_8PS]^-$; *m/z* measured: 569.1336 $[C_{20}H_{26}N_8O_8PS]^-$. Deviation: 0.2 ppm.

Coupling with desthiobiotin

The deprotected nucleotide was dissolved in aqueous 0.1 M $NaHCO_3$ solution (pH 8.7) and reacted with desthiobiotin-NHS (2.0 eq) dissolved in DMF overnight at room temperature. The solvents were removed under reduced pressure und the residue purified by RP-HPLC. After repeated freeze drying, product was obtained as its triethylammonium salt.

*N*6-Desthiobiotin-adenosine-5'-methylene bisphosphonate (4)

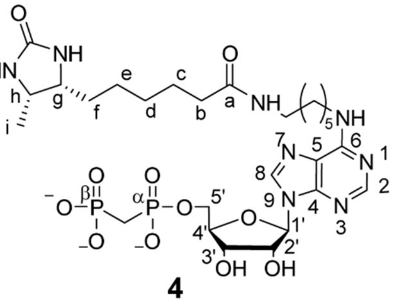

4

*N*6-(6-Aminohexyl)-adenosine 5'-methylene bisphosphonate (0.09 mmol, 1,0 eq) was coupled with desthiobiotin-NHS (0.06 g, 0.19 mmol, 2.0 eq) in aqueous $NaHCO_3$ solution (0.1 M, 6 mL, pH 8.7), following the general procedure. The product was obtained as a white powder as its triethylammonium salt (81 µmol, 86 %).

^{1}H NMR (400 MHz, MeOD-*d*$_4$): δ [ppm] = 8.53 (s, 1H. H-8), 8.23 (s, 1H, H-2), 6.07 (d, *J* = 5.5 Hz, 1H, H-1'), 4.69 (t, *J* = 5.3 Hz, 1H, H-2'), 4.49 (dd, *J* = 5.1, 3.2 Hz, 1H, H-3'), 4.26–4.16 (m, 3H, 4'-H, H-5'$_{a/b}$), 3.84–3.75 (m, 1H, H-h), 3.72–3.64 (m, 1H,H-g), 3.64–3.50 (m, 2H, *C*6-NH-C*H*$_2$), 3.17 (t, *J* = 6.9 Hz, 2H, RNH-C*H*$_2$), 2.25–2.10 (m, 4H, P−C*H*$_2$−P, H-b), 1.70 (p, *J* = 7.2 Hz, 2H, *C*6-NH-CH$_2$-C*H*$_2$), 1.62 (p, *J* = 7.2 Hz, 2H, H-c), 1.57–1.28 (m, 12H, NHR-CH$_2$-C*H*$_2$-C*H*$_2$-C*H*$_2$, H-f, H-d, H-e), 1.08 (d, *J* = 6.4 Hz, 3H, H-i).

^{13}C NMR (126 MHz, MeOD-*d*$_4$): δ [ppm] = 176.1 (C-a), 166.2 (NH−*C*(=O)−NH), 156.2 (C-6), 154.0 (C-2), 148.1 (only in 2D spectra, C-4), 140.6 (C-8), 120.5 (C-5), 89.0 (C-1'), 85.6 C-4'), 76.0 (C-2'), 72.0 (C-3'), 65.4 (C-5'), 57.4 (C-g), 52.7 (C-h), 40.3 (2 × RNH-*C*H$_2$), 37.0 (C-b), 30.7 (C-f), 30.4 and 27.1 (C-d and C-e), 30.2 (2 × RNH-CH$_2$-*C*H$_2$), 29.6 (P−*C*H$_2$−P), 27.7 (2 × RNH-CH$_2$-CH$_2$-*C*H$_2$), 26.9 (C-c), 15.7 (C-i).

^{31}P NMR (162 MHz, MeOD-*d*$_4$): δ [ppm] = 17.28 (s, 1P, P$_\alpha$) 13.45 (d, *J* = 6.2 Hz, 1P, P$_\beta$).

HR-ESI-MS: *m/z* calculated: 719.2689 [$C_{27}H_{45}N_8O_{11}P_2$]$^-$; *m/z* measured: 719.2689 [$C_{27}H_{45}N_8O_{11}P_2$]$^-$. Deviation: 0.1 ppm.

*C*2-Desthiobiotin-adenosine-5'-methylene bisphosphonate (9)

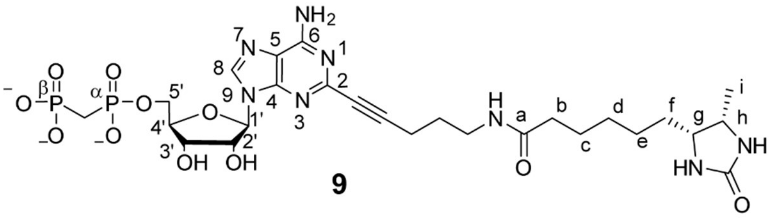

9

*C*2-(5-Aminopent-1-yn-1-yl)-adenosine 5'-methylene bisphosphonate (0.09 mmol, 1.0 eq) was coupled with desthiobiotin-NHS (0.06 g, 0.18 mmol, 2.0 eq) in aqueous $NaHCO_3$ solution (0.1 M, 6 mL, pH 8.7), following the general procedure. The product was obtained as a white powder as its triethylammonium salt (70 µmol, 79 %).

^{1}H NMR (400 MHz, MeOD-*d*$_4$): δ [ppm] = 8.59 (s, 1H, H-8), 6.05 (d, *J* = 5.3 Hz, 1H, H-1'), 4.64 (t, *J* = 5.3 Hz, 1H, H-2'), 4.50–4.45 (m, 2H, H-3'), 4.25 - 4.18 (m, 1H, H-4', H-5'$_{a/b}$), 3.85–3.75 (m, 1H, H-h), 3.73–3.64 (m, 1H, H-g), 3.35 (t, *J* = 6.7 Hz, 2H, C*H*$_2$−NHR), 2.50 (t, *J* = 7.1 Hz, 2H, C≡C−C*H*$_2$), 2.22 (t, *J* = 7.4 Hz, 2H, H-b), 2.17 (t, *J* = 19.2 Hz, 2H, P−C*H*$_2$−P), 1.84 (p, *J* = 6.9 Hz, 2H, CH$_2$−C*H*$_2$−CH$_2$), 1.63 (q, *J* = 7.1 Hz, 2H, H-c), 1.51–1.43 (m, 2H, H-f), 1.43–1.31 (m, 4H, H-d, H-e), 1.09 (d, *J* = 6.5 Hz, 3H, H-i).

^{13}C NMR (101 MHz, MeOD-d_4): δ [ppm] = 174.2 (C-a), 164.0 (NH−C(=O)−NH), 154.9 (C-6), 148.9 (C-4), 145.6 (C-2), 139.6 (C-8), 117.4 (C-5), 86.9 (C-1'), 85.2 (−C≡C−NHR), 83.5 (C-4'), 79.5 (−C≡C−NHR), 74.1 (C-2'), 69.7 (C-3'), 63.1 (C-5'), 55.3 (C-g), 50.5 (C-h), 37.5 (−CH_2−NHR), 34.9 (C-b), 28.6 (C-f), 28.1 and 25.0 (C-d and C-e), 26.9 (P−CH_2−P, CH_2−CH_2−CH_2), 24.7 (C-c), 15.3 (−C≡C−CH_2−), 13.5 (C-i) ppm.

^{31}P NMR (162 MHz, MeOD-d_4): δ [ppm] = 17.20 (d, J = 6.6 Hz, P_α), 13.38 (d, J = 6.3 Hz, P_β).

HR-ESI-MS: *m/z* calculated: 701.2219 $[C_{26}H_{39}N_8O_{11}P_2]^-$; *m/z* measured: 701.2216 $[C_{26}H_{39}N_8O_{11}P_2]^-$. Deviation: 0.4 ppm.

General procedure for the synthesis of nhAp$_3$A (Ap$_s$pCH$_2$pA)[1]

All of the nhAp$_3$A analogs were synthesized based on a procedure published by Blackburn *et al.*[1] with several adjustments. First, the triethylammonium salt of the 5'-nucleoside thiophosphate (1.0 eq) was shaken with trioctylamine (1.2 eq) in dry MeOH. After removing the solvent under reduced pressure, the residue was dissolved in dry dioxane. To the solution diphenyl phosphoryl chloride (1.3 eq) and tributylamine (2.4 eq) were added and the mixture was stirred for 40 min at room temperature. The solvents were removed under reduced pressure. The tetrabutylammonium salt of the respective 5'-nucleoside bisphosphonate (1.4–2.3 eq) was dissolved in dry pyridine and added to the activated thiophosphate. The reaction mixture was stirred at room temperature overnight. After removing the solvents under reduced pressure, water was added to the residue. The mixture was washed with DCM (3 × 10 mL) and then concentrated. The resulting crude product was purified with anion-exchange chromatography (Dionex DNAPac PA-100) and RP-HPLC. After repeated freeze drying, the product was obtained as diastereomeric mixture as its triethylammonium salt.

Comp-1

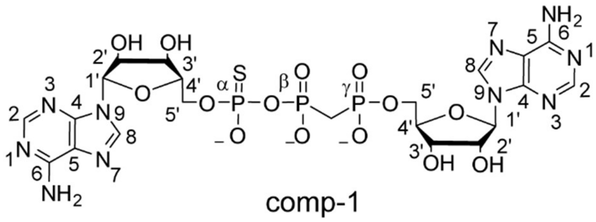

comp-1

Comp-1 was prepared following the general procedure starting from adenosine 5'-thiomonophosphate (**1**, 65 µmol, 1.0 eq) and adenosine-5'-methylene bisphosphonate (**3**, 149 µmol, 2.3 eq). The product was isolated as a white powder as its triethylammonium salt (4.5 µmol, 7 %).

^{1}H NMR (400 MHz, MeOD-d_4): δ [ppm] = 8.74 (s, 1H, 1 x H-8), 8.70 (s, 1H, 1 x H-8), 8.17 (s, 2H, 2 × H-2), 6.09 (t, 2H, 2 × H-1'), 4.79–4-69 (m, 2H, 2 × H-2'), 4.69–4.62 (m, 1H, 1 × H-3'), 4.59–4.53 (m, 1H, 1 × H-3'), 4.37 (t, J = 9.4 Hz, 1H, 1 × H-4'), 4.31–4.18 (m, 5H, 1 × H-4', 2 × $5'_{a/b}$), 2.72–2.54 (m, 2H, P-CH_2-P).

^{31}P NMR (162 MHz, MeOD-d_4): δ [ppm] = 43.82 (d, J = 33.1 Hz, P_γ), 16.58 (m, 1P, P_α), 6.25 (m, 1P, P_β) ppm.

HR-ESI-MS *m/z* calculated: 769.0725 $[C_{21}H_{28}N_{10}O_{14}P_3S]^-$; *m/z* measured: 769.0711 $[C_{21}H_{28}N_{10}O_{14}P_3S]^-$. Deviation: 1.82 ppm.

PALP-1

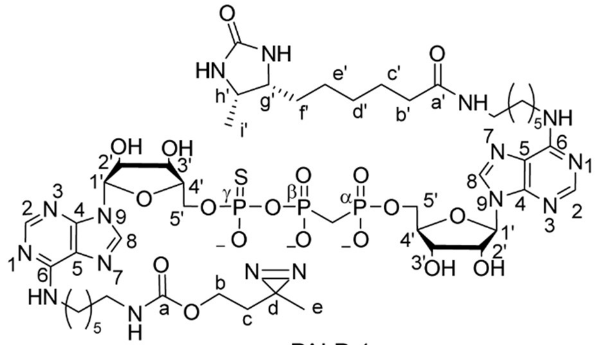

PALP-1

PALP-1 was prepared following the general procedure starting from *N*6-diazirine-adenosine 5'-thiomonophosphate (**2**, 49 µmol, 1.0 eq) and *N*6-desthiobiotin-adenosine-5'-methylene bisphosphonate (**4**, 90 µmol, 1.8 eq). The product was isolated as a white powder in its triethylammonium salt (13.1 µmol, 27 %).

^{1}H NMR (400 MHz, MeOD-d_4): δ [ppm] = 8.81–8.64 (m, 1H, two diastereoisomers, H-8), 8.60 (s, 1H, H-8), 8.20 (s, 1H, H-2), 8.19 (s, 1H, H-2), 6.12–6.05 (m, 2H, 2 × H-1'), 4.82–4.71 (m, 2H, 2 × H-2'), 4.68–4.60 (m, 1H, 1 × H-3'), 4.60–4.54 (m, 1H, 1 × H-3'), 4.44–4.16 (m, 6H, 2 × H-4', 2 × H-5'$_{a/b}$), 3.97 (t, *J* = 6.3 Hz, 2H, H-b), 3.84–3.75 (m, 1H, H-h'), 3.68 (q, *J* = 7.0 Hz, 1H, H-g'), 3.64–3.45 (m, 4H, 2 × *C*6-NH-C*H̲₂*), 3.17 (t, *J* = 6.9 Hz, 2H, RNH-C*H̲₂*), 3.11 (t, *J* = 6.9 Hz, 2H, RNH-C*H̲₂*), 2.17 (t, *J* = 7.4 Hz, 2H, P-C*H̲₂*-P), 1.69 (p, *J* = 7.2 Hz, 4H, 2 × C6-NH-CH$_2$-C*H̲₂*), 1.61 (q, *J* = 6.4 Hz, 4H, H-c, H-c') 1.56–1.23 (m, 18H, 2 × HR-CH$_2$-C*H̲₂*-C*H̲₂*-C*H̲₂*, H-f', H-d', H-e'), 1.08 (d, *J* = 6.5 Hz, 3H, H-i'), 1.03 (s, 3H, H-e).

^{13}C NMR (201 MHz, MeOD-d_4): δ [ppm] = 176.0 (C-a'), 166.1 (NH−*C̲*(=O)−NH), 158.7 (C-a), 155.9 (C-6), 153.8 (C-2), 150.1 (C-4), 140.8 (C-8), 120.1 (C-5), 89.0 (C-1'), 85.6 (C-4'), 76.4 (C-2'), 71.8 (C-3'), 66.8 (C-5'), 65.3 (C-5'), 60.8 (C-b), 57.4 (C-g'), 52.7 (C-h'), 41.8 (2 × RNH-*C̲*H$_2$), 40.4 (2 × RNH-*C̲*H$_2$), 37.0 (C-b'), 35.3 (C-c), 30.8–30.1 (C-d'/e', C-f', 4 × RNH-CH$_2$-*C̲*H$_2$), 27.7–27.1 (P-CH$_2$-P, C-d'/e', 4 × RNH-CH$_2$-CH$_2$-*C̲*H$_2$), 26.8 (C-c'), 24.9 (C-d), 20.0 (C-e), 15.6 (C-i').

^{31}P NMR (162 MHz, MeOD-d_4): δ [ppm] = 42.88 (m, 1P, P_γ), 15.79 (m, 1P, P_α), 5.05 (m, 1P, P_β).

HR-ESI-MS *m/z* calculated: 644.2195 $[C_{48}H_{75}N_{16}O_{18}P_3S]^{2-}$; *m/z* measured: 644.2191 $[C_{48}H_{75}N_{16}O_{18}P_3S]^{2-}$. Deviation: 0.62 ppm.

PALP-3

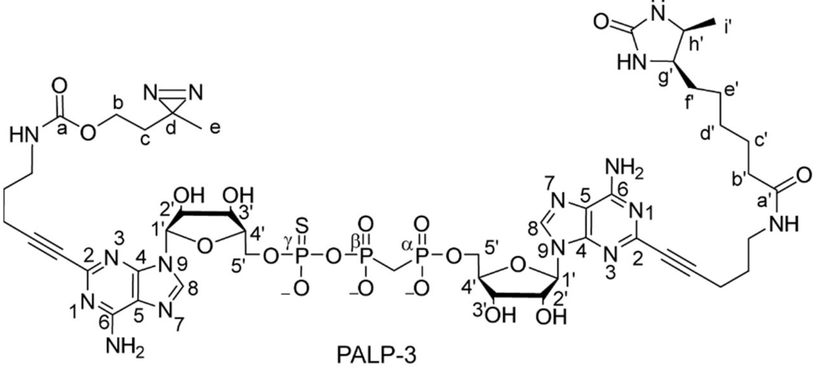

PALP-3

PALP-3 was prepared following the general procedure starting from *C*2-diazirine-adenosine 5'-thiomonophosphate **8** (24 µmol, 1.0 eq) and *C*2-desthiobiotin-adenosine-5'-methylene bisphosphonate **9** (34 µmol, 1.4 eq). The product was isolated as a white powder in its triethylammonium salt (0.43 µmol, 1.8 %).

^{1}H NMR (800 MHz, MeOD-d_4): δ [ppm] = 8.87–8.74 (m, 1H, two diastereoisomers, H-8), 8.70 (s, 1H, H-8), 6.11–6.01 (m, 2H, 2 × H-1'), 4.70–4.65 (m, 2H, 2 × H-2'), 4.65–4.59 (m, 1H, 1 × H-3'), 4.55 (t, J = 4.4 Hz , 1H, 1 × H-3'), 4.39–4.20 (m, 6H, 2 × H-4', 2 × H-5'$_{a/b}$), 3.99 (t, J = 6.3 Hz, 2H, H-b), 3.81–3.77 (m, 1H, H-h'), 3.67 (q, J = 7.3 Hz, 1H, H-g'), 3.35 (dt, J = 6.3, 1.7 Hz, 2H, C*H₂*−NHR), 3.28 (t, J = 6.7 Hz, 2H, C*H₂*−NHR), 2.52–2.48 (m, 4H,2 × C≡C−C*H₂*), 2.21 (t, J = 7.5, 2H, P-C*H₂*-P), 1.84 (p, J = 6.7 Hz, 4H, 2 × CH_2−C*H₂*−CH_2), 1.65–1.59 (m, 4H, H-c, H-c') 1.47 (q, J = 7.7 Hz, 2H, H-f'), 1.42–1.30 (m, 4H, H-d', H-e'), 1.08 (d, J = 6.5 Hz, 3H, H-i'), 1.03 (s, 3H, H-e).

^{31}P NMR (324 MHz, MeOD-d_4): δ [ppm] = 43.35 (m, 1P, P_γ), 16.14 (m, 1P, P_α), 5.84 (m, 1P, P_β).

HR-ESI-MS *m*/*z* calculated: 626.1714 $[C_{46}H_{63}N_{16}O_{18}P_3S]^{2-}$; *m*/*z* measured: 626.1715 $[C_{46}H_{63}N_{16}O_{18}P_3S]^{2-}$. Deviation: 0.16 ppm.

General procedure for the synthesis of nhAp$_4$A (Ap$_s$pCH$_2$pp$_s$A)[2]

All of the nhAp$_4$A analogs were synthesized following a procedure published by Yanachkov *et al.*[2] As a first step methylene bisphosphonic acid (1.0 eq) was coevaporated with tributylamine (2.2 eq) in dry DMF (3 × 5 mL) and activated with carbonyl diimidazole (5.0 eq) in dry DMF. The reaction was stirred at room temperature for 15 min. The excess of CDI was decomposed by the addition of water (100 µL) and the mixture was evaporated. The crude product (**6**) was used in the next steps without further purification (93 % conversion, determined by ^{31}P NMR shifts). The triethylammonium salts of nucleoside 5'-thiomonophosphates (4.0 eq) were coevaporated with dry DMF (2 × 5 mL). The diimidazolide (**6**) was suspended in dry DMF (1.0 mL) and added to the respective adenosine 5'-thiomonophosphate derivative. To catalyze the coupling reaction tetrazole in acetonitrile (0.45 M, 1.4 eq) was added. After stirring for 4 to 12 h at room temperature, the reaction was quenched with TEAB buffer (1.0 M, 2 mL) and purified via RP-HPLC. A diastereomeric mixture of the triethylammonium salt was obtained.

Ap$_s$pCH$_2$pp$_s$A (comp-2)

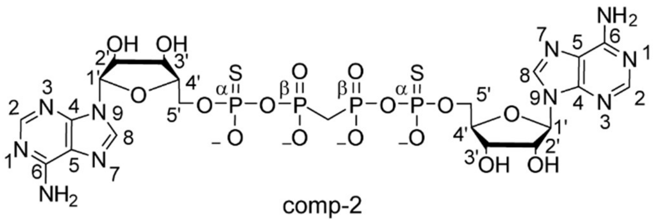

comp-2

The triethylammonium salt of adenosine 5'-thiophosphate (**1**, 0.04 g, 0.11 mmol, 4.0 eq) was reacted with activated methylene bisphosphonic acid (**6**, 0.03 mmol, 1.0 eq) according to the general procedure and the product was isolated in 5 % yield (5.5 µmol).

^{1}H NMR (400 MHz, D_2O): δ [ppm] = 8.64 (dd, J = 42.2, 5.3 Hz, 2H, 2 × H-8), 8.27 (d, J = 3.5 Hz, 2H, 2 × H-2), 6.03 (d, J = 5.0 Hz, 2H, 2 × H-1'), 4.65–4.57 (m, 2H, 2 × H-2'), 4.52 (p, J = 4.5 Hz, 2H, 2 × H-3'), 4.46 (s, 2H, 2 × H-4'), 4.37 (d, J = 5.0 Hz, 4H, 2 × H-5'$_{a/b}$), 2.82 (td, J = 21.0, 11.0 Hz, 2H, P-C*H₂*-P).

^{31}P NMR (162 MHz, D_2O): δ [ppm] = 43.10 (d, J = 31.4 Hz, 2P, P_α), 7.68 (d, J = 31.5 Hz, 2P, P_β).

^{13}C NMR (151 MHz, D_2O): δ [ppm] = 152.5 (2 × C-6), 149.9 (2 × C-2), 147.8 (2 × C-4), 140.8 (2 × C-8), 117.5 (2 × C-5), 87.2 (2 × C-1'), 83.7 (2 × C-4'), 75.2 (2 × C-2'), 70.4 (2 × C-3'), 65.5 (2 × C-5'), 31.3 (t, J = 130.3 Hz, P-*C*H$_2$-P).

HR-ESI-MS: *m/z* calculated: 865.0195 $[C_{21}H_{29}N_{10}O_{16}P_4S_2]^-$; *m/z* measured: 865.0160 $[C_{21}H_{29}N_{10}O_{16}P_4S_2]^-$. Deviation: 4.0 ppm.

*N*6-(6-Trifluoroacetamidohexyl) adenosine Ap$_S$pCH$_2$pp$_S$A (7)

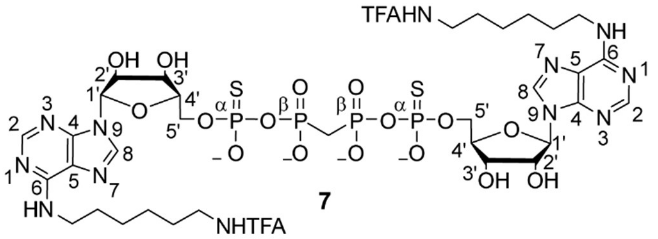

7

The triethylammonium salt of *N*6-(6-trifluoroacetamidohexyl)-adenosine 5'-thiophosphate (**5**, 0.16 g, 0.29 mmol, 4.0 eq) was reacted with activated methylene bisphosphonic acid (**6**, 0.02 g, 0.07 mmol, 1.0 eq) according to the general procedure and the product was isolated in 15 % yield (0.01 mmol).

^{1}H NMR (400 MHz, D_2O): δ [ppm] = 8.60 (d, *J* = 15.2 Hz, 1H, 1 x H-8), 8.38 (d, *J* = 23.7 Hz, 1H, 1 x H-8), 8.14 (s, 2H, 2 x H-2), 6.08 (dd, *J* = 15.1, 6.3 Hz, 2H, 2 x H-1'), 4.93–4.83 (m, 2H, 2 x H-2'), 4.67 (d, *J* = 37.6 Hz, 2H, 2 x H-3'), 4.39 (s, 2H, 2 x H-4'), 4.35–4.19 (m, 4H, 2 x H-5'$_{a/b}$), 3.43 (bs, 4H, 2 x C6-NH-C*H$_2$*), 3.32 (t, *J* = 7.0 Hz, 4H, 2 x NHTFA-C*H$_2$*), 2.80–2.61 (m, 2H, P-CH$_2$-P), 1.82–1.46 (m, 8H, 2 x C6-NH-CH$_2$-CH$_2$, 2 x NHTFA-CH$_2$-C*H$_2$*)1.51–1.33 (m, 8H, 2 x C6-NH-CH$_2$-CH$_2$-C*H$_2$*, 2 x NHTFA- CH$_2$-CH$_2$-C*H$_2$*).

^{31}P NMR (162 MHz, D_2O): δ [ppm] = 42.85–41.29 (m, 2P, P_α), 8.24–6.05 (m, 2P, P_β).

^{19}F NMR (376 MHz, D_2O): δ [ppm] = −75.75 (s, 6F, 2 x C*F$_3$*).

^{13}C NMR (151 MHz, D_2O): δ [ppm] =158.7 (2 × *C*(O)-CF$_3$), 153.4 (2 × C-6), 152.0 (2 × C-2), 148.2 (1 × C-4), 147.3 (1 × C-4), 139.5 (1 × C-8), 139.0 (1 × C-8), 116.8 (2 × C-5), 114.9 (1 × CF$_3$) , 113.0 (1 × CF$_3$), 86.9 (1 × C-1'), 86.6 (1 × C-1'), 83.7 (1 × C-4'), 83.6 (1 × C-4'), 74.5 (2 × C-2'), 70.5 (2 × C-3'), 65.2 (1 × C-5'), 64.7 (1 × C-5'), 39.8 (2 × RNH-*C*H$_2$), 39.7, (2 × RNH-*C*H$_2$), 28.1 (P-*C*H$_2$-P), 27.6 (2 × C6-NH-CH$_2$-*C*H$_2$, 2 × NHTFA-CH$_2$-*C*H$_2$), 25.6 (4 × CH$_2$).

HR-ESI-MS: *m/z* calculated: 627.0904 $[C_{37}H_{52}F_6N_{12}O_{18}P_4S_2]^{2-}$; *m/z* measured: 627.0969 $[C_{37}H_{52}F_6N_{12}O_{18}P_4S_2]^{2-}$. Deviation: 1.02 ppm.

Modification of nhAp$_4$A[3]

Deprotection

For the deprotection of the non-hydrolysable Ap$_4$A, derivative **7** (4.37 µmol) was dissolved in aqueous NH_3 (10 %, 400 µL) and stirred at room temperature for 2 h. The progress of the reaction was monitored by ^{19}F NMR. The product was isolated by RP-HPLC in 63 % yield (2.75 µmol) as its triethylammonium salt.

Coupling with DTB

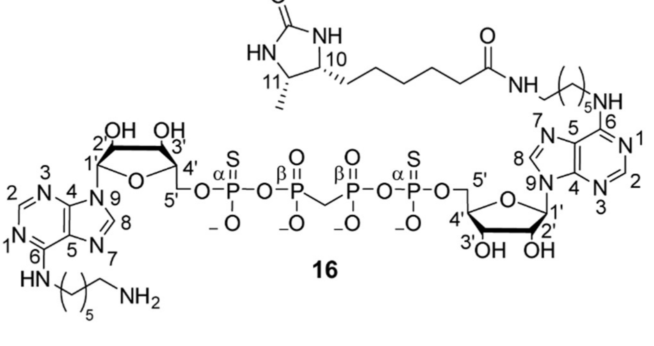

16

Deprotected nhAp_4A (2.75 µmol, 1.0 eq) was dissolved in $NaHCO_3$ (0.1 M, pH 8.7, 0.5 mL) and reacted with desthiobiotin-NHS (1.82 µmol, 0.7 eq) in DMF (0.5 mL). The product was obtained after RP-HPLC purification in 33 % yield (0.91 µmol), while the starting material was recovered (25 %, 0.69 µmol).

^{1}H NMR (400 MHz, D_2O): δ [ppm] = 8.59 (dd, *J* = 14.4, 5.2 Hz, 1H, 1 × H-8), 8.39 (dd, *J* = 22.6, 5.9 Hz, 1H, 1 × H-8), 8.14 (d, *J* = 2.2 Hz, 2H, 2 × H-2), 6.09 (dt, *J* = 14.3, 6.7 Hz, 2H, 2 × H-1'), 4.66–4.58 (m, 4H, 2 × H-2', 2 × H-3'), 4.43–4.35 (m, 2H, 2 × H-4'), 4.35–4.19 (m, 4H, 2 × H-5'$_{a/b}$) 3.72 (p, *J* = 6.8 Hz, 1H, H-11), 3.64–3.55 (m, 1H, H-10), 3.54–3.29 (bs, 2H, 2 × C6-NH-CH_2), 3.20 (t, *J* = 6.3 Hz, 4H, 2 × NHR-C$\underline{H_2}$), 2.73 (ddd, *J* = 19.1, 12.7, 7.7 Hz, 2H, P-C$\underline{H_2}$-P), 2.20 (t, *J* = 6.7 Hz, 2H, CH_2-C(NR)=O), 1.69–1.60 (m, 8H, 2 × C6-NH-CH_2-C$\underline{H_2}$, 2 × NHR-CH_2-C$\underline{H_2}$), 1.59–1.49 (m, 8H, 2 × C6-NH-CH_2-CH_2-C$\underline{H_2}$, 2 × NHR-CH_2-CH_2-C$\underline{H_2}$), 1.46–1.28 (m, 8H, 4 × CH_2-DTB), 0.95 (d, *J* = 6.5 Hz, 3H, CH_3).

^{31}P NMR (162 MHz, D_2O): δ [ppm] = 42.54–41.97 (m, 2P, P_α), 6.95 (ddd, *J* = 34.8, 20.2, 15.3 Hz, 2P, P_β).

ESI-MS *m/z* calculated: 1259.3468 $[C_{43}H_{71}N_{14}O_{18}P_4S_2]^-$; *m/z* measured: 1259.3526 $[C_{43}H_{71}N_{14}O_{18}P_4S_2]^-$. Deviation: 4.6 ppm.

Coupling with diazirine (PALP-2)

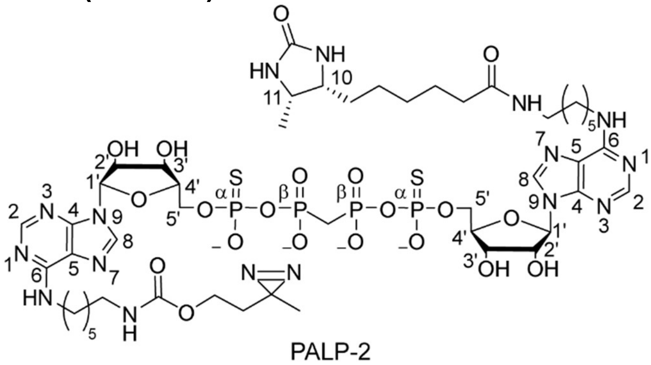

PALP-2

Compound **16** (3.55 µmol, 1.0 eq) was dissolved in $NaHCO_3$ (0.1 M, pH 8.7, 0.5 mL) and reacted with diazirine-NHS[6] (17.75 µmol, 5.0 eq) in DMF (0.5 mL). The product was isolated by RP-HPLC in 27 % yield (0.95 µmol).

^{1}H NMR (400 MHz, D_2O): δ [ppm] = 8.59 (d, *J* = 12.8 Hz, 1H, 1 × H-8), 8.38 (d, *J* = 22.8 Hz, 1H, 1 × H-8), 8.15 (s, 2H, 2 × H-2), 6.08 (dd, *J* = 9.4, 6.1 Hz, 2H, 2 × H-1'),4.75–4.70 (m, 2H, 2 × H-2'), 4.70–4.53 (m, 2H, 2 × H-3'), 4.43–4.35 (m, 2H, 2 × H-4'), 4.33–4.19 (m, 4H, 2 × H-5'$_{a/b}$), 4.00 (t, *J* = 5.9 Hz, 2H, NH-COO-C$\underline{H_2}$), 3.75–3.70 (m, 1H, H-11), 3.65–3.55 (m, 1H, H-10), 3.51–3.31 (m, 2H, 2 × C6-NH-C$\underline{H_2}$), 3.17–3.10 (m, 4H, 2 × NHR-C$\underline{H_2}$), 2.99–2.86 (m, 2H, P-C$\underline{H_2}$-P) 2.21 (t, *J* = 7.2 Hz, 2H, CH_2-C(NR)=O), 1.69–1.61 (m, 6H, 2 × CH_2-DTB, 1 × CH_2-DA), 1.58–1.51 (m, 8H, 4 × CH_2-linker), 1.50–1.36 (m, 8H, 4 × CH_2-linker), 1.29–1.17 (m, 4H, 2 × CH_2-linker), 1.04–0.91 (m, 6H, 1 × CH_3-DTB, 1 × CH_3-DA).

^{31}P NMR (162 MHz, D_2O): δ [ppm] = 43.11–41.70 (m, 2P, P_α), 7.51–6.65 (m, 2P, P_β).

^{13}C NMR (201 MHz, D_2O): δ [ppm] = 176.7 (DTB-$\underline{C}$-(O)-OR), 165.4 (DTB-$\underline{C}$-(O)-$(NHR)_2$), 152.2 (C-6), 147.5 (C-2), 139.5 (C-8), 86.8 (C-1'), 83.8(C-4'), 74.6 (C-2'), 70.5 (C-3'), 65.3 (C-5'), 55.8 (C-10), 51.3 (C-11), 39.3 (4 × RNH-CH_2), 35.6 ($\underline{C}H_2$-C(NR)=O), 28.5 (P-CH_2-P), 27.9 (4 ×CH_2-linker), 26.5 (4 × CH_2-linker), 25.7 (2 × CH_2-DTB), 25.2 (2 × CH_2-DTB, 2 × CH_2-DA), 14.2 (1 × CH_3-DTB, 1 × CH_3-DA).

HR-ESI-MS: *m/z* calculated: 1385.3886 $[C_{48}H_{77}N_{16}O_{20}P_4S_2]^-$; *m/z* measured: 1385.3928 $[C_{48}H_{77}N_{16}O_{20}P_4S_2]^-$. Deviation: 3.0 ppm.

m/z calculated: 692.1901 $[C_{48}H_{76}N_{16}O_{20}P_4S_2]^{2-}$; *m/z* measured: 692.1879 $[C_{48}H_{76}N_{16}O_{20}P_4S_2]^{2-}$. Deviation: 3.3 ppm.

Synthesis of con-1

Control compound scaffold-precursor (17)

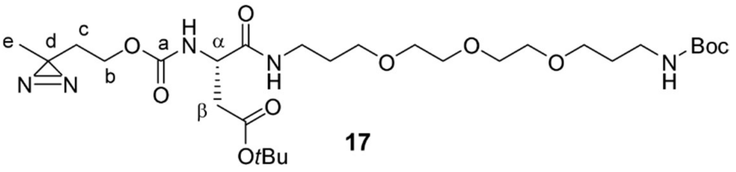

17

N-Boc-*N'*-Asp(tBu)-4,7,10-trioxa-1,13-tridecanediamine was synthesized according to the procedure published by Dalhoff *et al.* starting from commercially available *N*-Boc-4,7,10-trioxa-1,13-tridecanediamine and Z-Asp(*t*Bu)OH.[7]

To obtain control compound scaffold-precursor **17** *N*-Boc-*N'*-Asp(tBu)-4,7,10-trioxa-1,13-tridecanediamine (1.00 g, 2.03 mmol, 1.0 eq) was dissolved in DCM (10 mL). Et_3N (0.56 mL, 0.41 g, 4.06 mmol, 2.0 eq) and diazirine-NHS[6] (0.54 g, 2.24 mmol, 1.1 eq) were added and the reaction mixture was stirred overnight at room temperature under protection from light. The solvent was removed under reduced pressure. Acetic acid (10 mL) was added to the residue and the mixture was stirred for 10 min. After evaporation of the solvent under reduced pressure, the resulting crude product was purified via RP-MPLC (acetonitrile/H_2O, 5 % → 40 % → 65 %), yielding product **17** (0.72 g, 1.17 mmol, 58 %) as a yellow oil.

^{1}H NMR (400 MHz, DMSO-*d*$_6$): δ [ppm] = 7.81 (t, *J* = 6.4 Hz, 1H, PEG-N*H*), 7.39 (d, *J* = 8.5 Hz, 1H, amino acid-N*H*), 6.73 (t, *J* = 5.7 Hz, 1H, N*H*-Boc), 4.28 (td, *J* = 8.6, 5.5 Hz, 1H, H-α), 3.99–3.80 (m, 2H, H-b), 3.55–3.42 (m, 8H, PEG-linker-C*H*$_2$), 3.37 (t, *J* = 6.3 Hz, 4H, PEG-linker-C*H*$_2$), 3.09 (q, *J* = 6.9 Hz, 2H, PEG-linker-C*H*$_2$), 2.95 (q, *J* = 6.6 Hz, 2H, PEG-linker-C*H*$_2$), 2.68–2.39 (m, 2H, H-β), 1.66–1.53 (m, 6H, PEG-linker-C*H*$_2$, H-c), 1.44–1.31 (m, 18H, tBu-C*H*$_3$, Boc-C*H*$_3$), 1.03 (s, 3H, H-e).

HR-ESI-MS: *m*/*z* calculated: 618.3709 $[C_{28}H_{52}N_5O_{10}]^+$; *m*/*z* measured: 618.3678 $[C_{28}H_{52}N_5O_{10}]^+$. Deviation: 5.01 ppm.

Control compound scaffold (18)

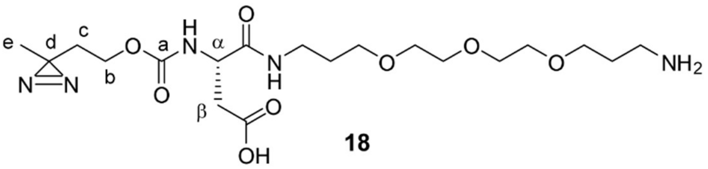

18

Control compound scaffold-precursor **17** (0.32 g, 0.52 mmol, 1.0 eq) was dissolved in dry DCM (1.0 mL). TFA (0.50 mL) and $HSiEt_3$ (0.20 mL, 1.51 mmol, 2.5 eq) were added and the reaction mixture was stirred overnight at room temperature under protection from light. Precooled Et_3N (1 mL) was added and the mixture quenched with water (0.3 mL). Removing the solvents under reduced pressure yielded the crude product as a yellow oil that was directly used for the next step without further purification.

Con-1

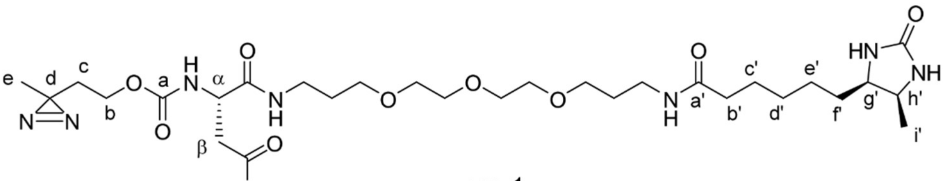

con-1

Control compound scaffold **18** (0.12 g, 0.26 mmol, 1.0 eq) was dissolved in THF (4 mL). Desthiobiotin-NHS (0.10 g, 0.33 mmol, 1.27 eq) and Et$_3$N (0.11 mL, 0.07 g, 0.73 mmol, 2.3 eq) were added and the reaction mixture stirred overnight at room temperature under protection from light. The solvents were evaporated *in vacuo* and the crude product was purified by RP-MPLC (acetonitrile/0.1 % TFA in water, 5 % → 40 % → 65 %), yielding con-1 (0.11 g, 0.17 mmol, 66 % over two steps) as a yellow oil.

^{1}H NMR (400 MHz, DMSO-d_6): δ [ppm] = 7.80 (t, J = 5.7 Hz, 1H, PEG-N*H*), 7.71 (t, J = 5.6 Hz, 1H, PEG-N*H*-desthiobiotin), 7.41 (d, J = 8.3 Hz, 1H, amino acid-N*H*), 4.31–4.22 (m, 1H, H-α), 3.94–3.85 (m, 2H, H-b), 3.65–3.55 (m, 1H, H-h'), 3.56–3.28 (m, 13H, H-g', 6 × PEG-linker-C*H*$_2$, behind solvent peak), 3.14–3.01 (m, 4H, 2 × PEG-linker-C*H*$_2$), 2.69–2.58 (m, 2H, H-β), 2.03 (t, J = 7.4 Hz, 2H, H-b'), 1.67–1.52 (m, 6H, c-H, 2 × PEG-linker-C*H*$_2$), 1.47 (p, J = 7.1 Hz, 2H, H-c'), 1.41–1.27 (m, 4H, H-d', H-f'), 1.27–1.13 (m, 2H, H-e'), 1.03 (s, 3H, H-e), 0.96 (d, J = 6.5 Hz, 3H, i' -H).

^{13}C NMR (126 MHz, DMSO-d_6): δ [ppm] = 172.0 (*C*(=O)NH), 171.8 (*C*(=O)NH), 170.5 (C-a), 162.8 (HN-*C*(=O)-NH), 162.3 (*C*OOH), 69.8 (PEG-linker-*C*H$_2$), 69.7 (PEG-linker-*C*H$_2$), 69.6 (2 × PEG-linker-*C*H$_2$), 68.1 (PEG-linker-*C*H$_2$), 68.0 (PEG-linker-*C*H$_2$), 59.3 (C-b), 55.0 (C-g'), 51.4 (C-α), 50.2 (C-h'), 36.1 (C-β), 35.8 (PEG-linker-*C*H$_2$), 35.7 (PEG-linker-*C*H$_2$), 35.4 (C-b'), 33.5 (C-c), 29.5 (PEG-linker-*C*H$_2$), 29.4 (PEG-linker-*C*H$_2$), 29.2 (DTB-*C*H$_2$), 28.7 (DTB-*C*H$_2$), 25.6 (C-e'), 25.2 (DTB-*C*H$_2$), 24.4 (C-d), 19.5 (C-e), 15.5 (C-i').

HR-ESI-MS: *m*/*z* calculated: 658.3770 $[C_{29}H_{52}N_7O_{10}]^+$; *m*/*z* measured: 658.3773 $[C_{29}H_{52}N_7O_{10}]^+$. Deviation: 0.46 ppm.

Supplementary references

1 Blackburn, G. M., Liu, X., Rösler, A. & Brenner, C. Two Hydrolase Resistant Analogues of Diadenosine 5′, 5 ‴′-P, P-Triphosphate for Studies with FHIT, The Human Fragile Histidine Triad Protein. *Nucleosides & nucleotides* **17**, 301–308 (1998).

2 Yanachkov, I. B., Dix, E. J., Yanachkova, M. I. & Wright, G. E. P 1,P2-Diimidazolyl derivatives of pyrophosphate and bis-phosphonates - synthesis, properties, and use in preparation of dinucleoside tetraphosphates and analogs. *Org. Biomol. Chem.* **9**, 730–738 (2011).

3 Hacker, S. M. *et al.* Fingerprinting differential active site constraints of ATPases. *Chem. Sci.* **4**, 1588–1596 (2013).

4 Hofer, A. *et al.* Chemoselective Dimerization of Phosphates. *Org. Lett.* **18**, 3222–3225 (2016).

5 Mohamady, S., Desoky, A., Taylor, S. D. Sulfonyl Imidazolium Salts as Reagents for the Rapid and Efficient Synthesis of Nucleoside Polyphosphates and Their Conjugates. *Org. Lett*. **14**, 402–405 (2012).

6 Chou, C. *et al*. Genetically encoding an aliphatic diazirine for protein photocrosslinking. *Chem. Sci.* **2**, 480–483 (2011).

7 Dalhoff, C. *et al.* Synthesis of S-Adenosyl-L-homocysteine Capture Compounds for Selective Photoinduced Isolation of Methyltransferases. *ChemBioChem* **11**, 256–265 (2010).

8 Fischer, B. *et al.* 2-thioether 5'-O-(1-thiotriphosphate) adenosine derivatives as new insulin secretagogues acting through P2Y-Receptors. J. Med. Chem. **42**, 3636–3646 (1999).

9 Kalek, M. *et al*. A direct method for the synthesis of nucleoside 5′-methylenebis(phosphonate)s from nucleosides. *Tetrahedron Letters* **46**, 2417–2421 (2005).
