# Supplementary material for: Chemical proteomic profiling reveals protein interactors of the alarmones diadenosine triphosphate and tetraphosphate
Source: Nat Commun. 2021 Oct 4;12:5808. doi: 10.1038/s41467-021-26075-4 (PMC8490401; doi:10.1038/s41467-021-26075-4)
Supplement: Supplementary file 2 — Description of Additional Supplementary Files [file 41467_2021_26075_MOESM2_ESM.docx]

**Description of Additional Supplementary Files**

Supplementary Data 1:

Lists of proteins identified from HEK293T lysates using ApnA-based photoaffinity-labeling probes.

Supplementary Data 2:

GO term analysis of proteins significantly enriched in the performed PAL experiments with HEK293T lysates.

Supplementary Data 3:

Lists of proteins identified from E. coli K12 lysates using ApnA-based photoaffinity-labeling probes.

Supplementary Data 4:

GO term analysis of proteins significantly enriched in the performed PAL experiments with E. coli K12 lysates.
